# Supplementary material for: Prevalence of insufficient weight loss 5 years after Roux-en-Y gastric bypass: metabolic consequences and prediction estimates: a prospective registry study
Source: BMJ Open. 2021 Mar 2;11(3):e046407. doi: 10.1136/bmjopen-2020-046407 (PMC7929824; doi:10.1136/bmjopen-2020-046407)
Supplement: Supplementary data [file bmjopen-2020-046407supp001.pdf]

Online-only Supplements

eAppendix 1. Cut-offs used to define cardiometabolic disease

eAppendix 2. Sensitivity analysis

eAppendix 3. Examples of risk calculation

eFigure 1 a-g. Unadjusted mean participants with pharmacological treatment of dyslipidemia and/or type 2 diabetes or hypertension at baseline, stratified on surgical treatment failure

eFigure 2 a-g. Unadjusted mean participants without pharmacological treatment of dyslipidemia and/or type 2 diabetes or hypertension at baseline, stratified on surgical treatment failure

eFigure 3.a-c. Predicted probability of type 2 diabetes at year five after surgery plotted over a, percentage weight loss from baseline to year five, b, excess body mass index loss from baseline to year five, c, body mass index at year five

eFigure 4.a-c. Predicted probability of dyslipidemia at year five after surgery plotted over a, percentage weight loss from baseline to year five, b, excess body mass index loss from baseline to year five, c, body mass index at year five.

eFigure 5.a-c. Predicted probability of Hypertension at year five after surgery plotted over a, percentage weight loss from baseline to year five, b, excess body mass index loss from baseline to year five, c, body mass index at year five.

eTable 1. Modality of the one-, two-, and five year follow-ups

eTable 2. Comparison of baseline characteristics between included participants and those lost to follow-up at year five

eTable 3. Percentage of available data on pharmacological treatment, biochemistry and blood pressure.

eTable 4. Comparison of baseline characteristics between surgical treatment failure and non-surgical treatment failure

eTable 5. Odds of cardiometabolic disease at year five, separate models for each definitions of surgical treatment failure

eTable 6. Odds of Cardiometabolic disease at year five for subjects reaching one, two or three of the three definitions

Brissman M, *et al. BMJ Open* 2022; 11:e046407. doi: 10.1136/bmjopen-2020-046407

eAppendix 1. Cut-offs used to define cardiometabolic disease

LDL>3.0mmol/L
HDL <1.0mmol/L for males and <1.3mmol/L for females
Triglycerides >2.0mmol/L
Fasting glucose >7.0mmol/L
HbA1c >48mmol/mol
Systolic- >140mmHg or Diastolic blood pressure >90mmHg

Conversion to mg/dL is done by a multiplying factor of 38.67 for LDL and HDL, 88.57 for TG, 18 for fasting glucose and using the formula (0.09148*IFCC) + 2.152 for HbA1c(%).

Brissman M, *et al. BMJ Open* 2022; 11:e046407. doi: 10.1136/bmjopen-2020-046407

eAppendix 2. Sensitivity analysis

We compared participants lost to follow-up with those not lost and found that the former had a higher prevalence of surgical treatment failure at year one (12.3% vs. 10.3%, p=0.015) and a similar prevalence (13.3% vs. 11.6%) at year two (p=0.081). Within the sample, there was a strong carryover effect, as 73.5% who met the definition of surgical treatment failure in year one and 79.5% in year two also met the definition at year five.

Additionally, participants who visited the clinic for follow-up had a higher prevalence of surgical treatment failure (26.7%) compared with other modes of follow-up (20.2%, p<0.001).

No statistical differences in prevalence of surgical treatment failure at year five were evident between retention groups 60-70%, 70-80% and >80%, (23.8% vs. 23.4% vs. 21.4%) (p=0.235). We found no statistical difference according to the year of surgery (p=0.280), or surgical volume (<50 [23.7%] vs. ≥50 [23.0%] LRYGB per year, p=0.695).

There was a crude difference in the prevalence of surgical treatment failure between males and females (males 31.6% vs. females 20.9%, p<0.001): OR=1.46 (95%CI 1.26-1.69) after baseline adjustments for age, BMI, T2D, hypertension and dyslipidemia. Males experienced a lower %TWL from baseline to all follow-up periods (data not shown).

At baseline, T2D (25.1% vs. 12.4%) and hypertension (44.4% vs. 24.1%) were more common among males (both p<0.001), whereas dyslipidemia was more common among females (61.7% vs. 56.9%, p=0.002).

We also found an association between those who had cardiometabolic disease present at baseline and surgical treatment failure at year five. The presence of T2D at baseline was associated with surgical treatment failure (OR=1.70; 95%CI 1.44-2.00), as was dyslipidemia at baseline (OR=1.30; 95%CI 1.15-1.48) and hypertension at baseline (OR=1.16; 95%CI 1.01-1.34), all adjusted for sex, age and BMI.

In terms of the magnitude of prediction factors, four additional models were assessed, one omitting change in weight between year one and two (AUC = 0.8260); another omitting %TWL at year one (AUC = 0.7214). Third, we assessed a model adding cardiometabolic disease (T2D, dyslipidemia, hypertension) at baseline as additional predictors. With this latter model, we found that only dyslipidemia remained significant and improved the model minimally (AUC = 0.8749). We also assessed a model restricted to variables available at baseline that included sex, age, BMI and cardiometabolic disease (AUC = 0.638).

Brissman M, *et al. BMJ Open* 2022; 11:e046407. doi: 10.1136/bmjopen-2020-046407

eAppendix 3. Examples of calculation of predicted probability meeting one of the definitions of surgical treatment failure at year five.

Fictitious example 1: male=0, age 43 years, BMI = 45, -23%TWL during year one, +4 kg between year one and two

a = -1.1 + -0.00545*0 + 0.00299*43 + 0.14949*45 + 0.22310*-23 + 0.15982*4
exp(a)/(1+(a)) = 0.78

Hence, predicted probability of surgical treatment failure at year five is 78%.

Fictitious example 2: male=0, age 43 years, BMI = 49, -33%TWL during year one, -1 kg between year one and two,

a = -1.1 + -0.00545*0 + 0.00299*43 + 0.14949*49 + 0.22310*-33 + 0.15982*-1

This yields a predicted probability of 23.8% for surgical treatment failure at year five.

eFigure 1 a-g. Unadjusted mean values (bars) with error bars (95% confidence intervals) for participants <u>with</u> pharmacological treatment of dyslipidemia and/or type 2 diabetes or hypertension at baseline, stratified on surgical treatment failure. a) low density lipoprotein, b) high density lipoprotein, c) triglycerides, d) fasting glucose, e) HbA1c, f) systolic blood pressure, g) diastolic blood pressure. Tables below figures show number available at each timepoint and prevalence above cut-off. Bold font with * indicates a difference p<0.05).

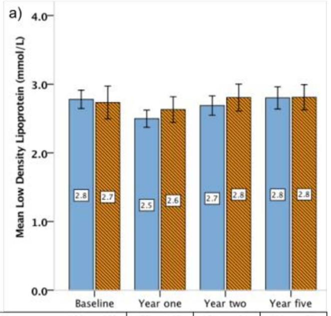

n	202	102	177	87	151	81	144	78
Above 4.1mmol/L (%)	8.9%	13.7%	4.5%	5.7%	3.3%	8.6%	8.3%	3.3%

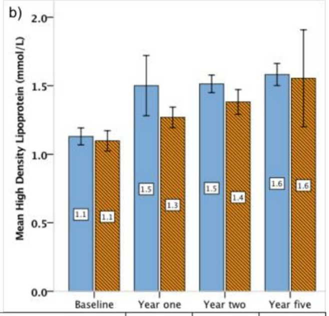

n	214	106	180	88	154	81	146	79
<1.0 | <1.3 mmol/L (%)	69.2%	61.3%	30.0%	42.0%	24.0%	29.6%	19.9%	**35.4%***

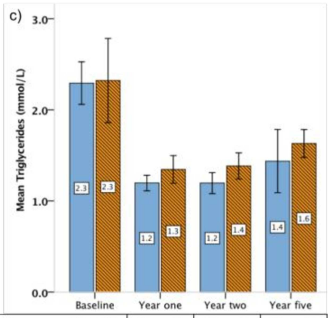

n	223	108	181	92	151	81	141	76
Above 2.0 mmol/L (%)	53.8%	49.1%	12.2%	16.3%	10.6%	**21.0%***	20.6%	**38.2%***

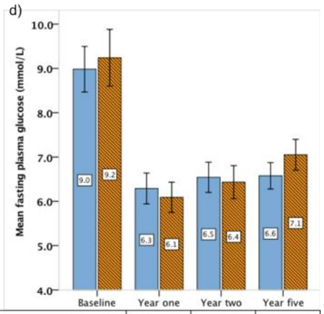

n	197	120	191	130	223	131	237	154
Above 7.0mmol/L (%)	65.0%	71.7%	21.5%	14.6%	25.6%	20.6%	27.0%	**39.6%***

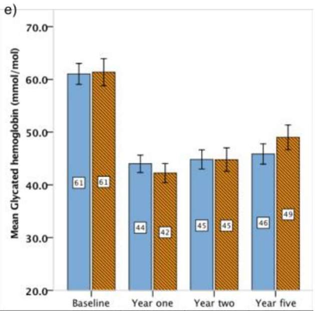

n	300	185	283	173	268	163	263	159
Above 48.0 mmol/mol (%)	75.7%	71.4%	21.9%	17.3%	26.5%	25.2%	27.8%	**40.9%***

Non surgical failure
Surgical failure

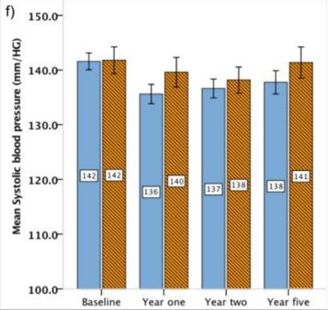

n	464	182	351	163	320	160	296	154
>140mm/HG	43.3%	43.4%	27.4%	**39.3%***	30.0%	33.8%	33.1%	41.6%

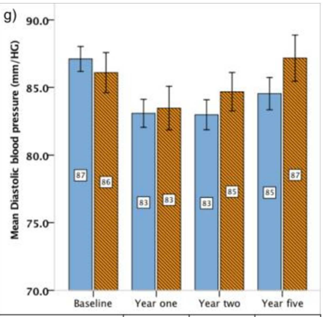

n	464	182	351	163	318	160	296	154
>90mm/HG	27.8%	26.4%	18.5%	20.9%	18.9%	19.4%	23.0%	30.5%

eFigure 2 a-g. Unadjusted mean values (bars) with error bars (95% confidence intervals) for participants <u>without</u> pharmacological treatment of dyslipidemia and/or type 2 diabetes or hypertension at baseline, stratified on surgical treatment failure. a) low density lipoprotein, b) high density lipoprotein, c) triglycerides, d) fasting glucose, e) HbA1c, f) systolic blood pressure, g) diastolic blood pressure. Tables below figures show number available at each timepoint and prevalence above cut-off. Bold font with * indicates a difference (p<0.05).

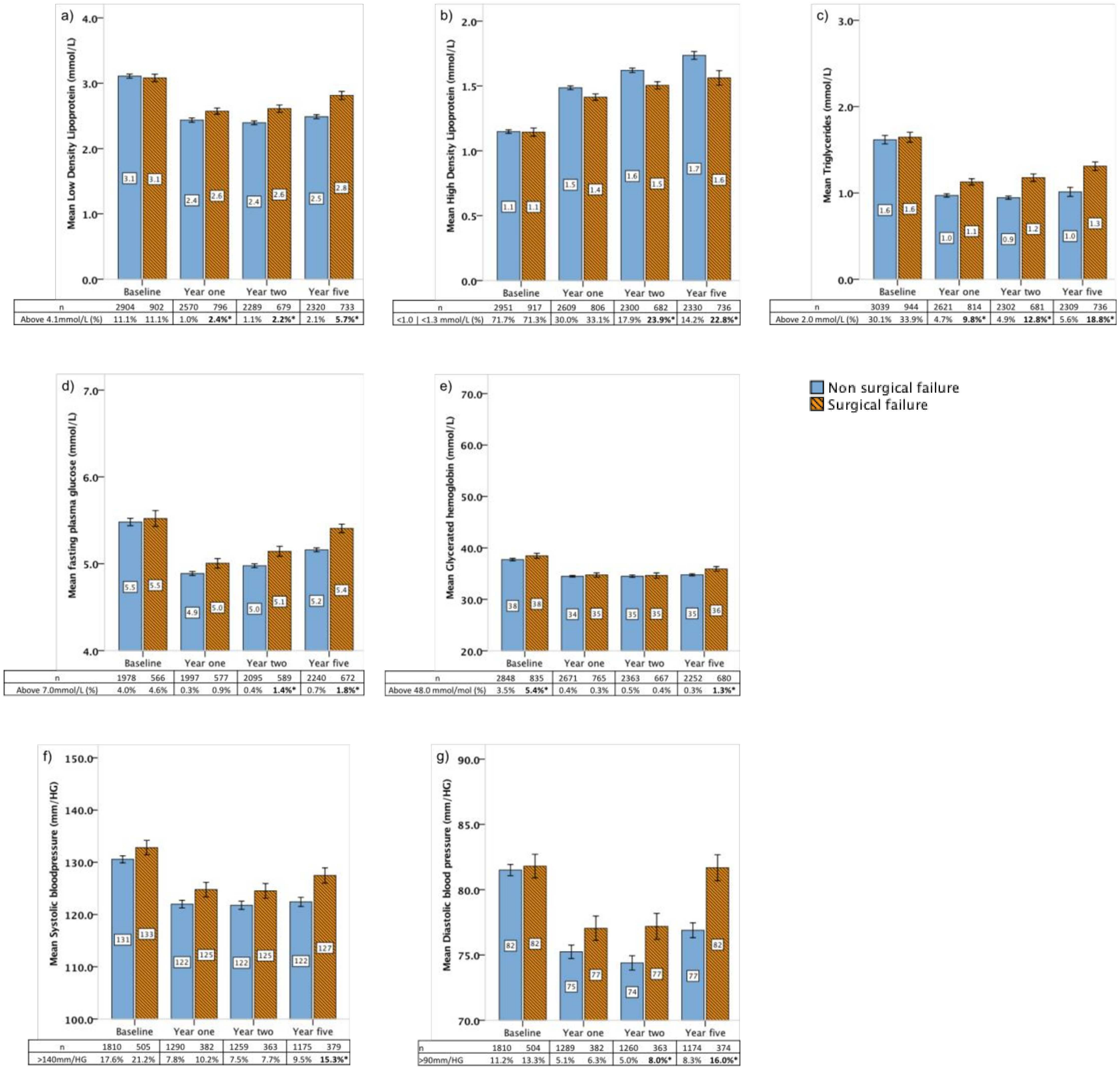

a)

	Baseline		Year one		Year two		Year five	
n	2904	902	2570	796	2289	679	2320	733
Above 4.1mmol/L (%)	11.1%	11.1%	1.0%	**2.4%***	1.1%	**2.2%***	2.1%	**5.7%***

b)

	Baseline		Year one		Year two		Year five	
n	2951	917	2609	806	2300	682	2330	736
<1.0 | <1.3 mmol/L (%)	71.7%	71.3%	30.0%	33.1%	17.9%	**23.9%***	14.2%	**22.8%***

c)

	Baseline		Year one		Year two		Year five	
n	3039	944	2621	814	2302	681	2309	736
Above 2.0 mmol/L (%)	30.1%	33.9%	4.7%	**9.8%***	4.9%	**12.8%***	5.6%	**18.8%***

d)

	Baseline		Year one		Year two		Year five	
n	1978	566	1997	577	2095	589	2240	672
Above 7.0mmol/L (%)	4.0%	4.6%	0.3%	0.9%	0.4%	**1.4%***	0.7%	**1.8%***

e)

	Baseline		Year one		Year two		Year five	
n	2848	835	2671	765	2363	667	2252	680
Above 48.0 mmol/mol (%)	3.5%	**5.4%***	0.4%	0.3%	0.5%	0.4%	0.3%	**1.3%***

f)

	Baseline		Year one		Year two		Year five	
n	1810	505	1290	382	1259	363	1175	379
>140mm/HG	17.6%	21.2%	7.8%	10.2%	7.5%	7.7%	9.5%	**15.3%***

g)

	Baseline		Year one		Year two		Year five	
n	1810	504	1289	382	1260	363	1174	374
>90mm/HG	11.2%	13.3%	5.1%	6.3%	5.0%	**8.0%***	8.3%	**16.0%***

Brissman M, *et al. BMJ Open* 2022; 11:e046407. doi: 10.1136/bmjopen-2020-046407

eFigure 3.a-c. Predicted probability of type 2 diabetes at year five after surgery plotted over a, percentage weight loss from baseline to year five, b, excess body mass index loss from baseline to year five, c, body mass index at year five. Groups based on presence on presence of type 2 diabetes at baseline. Adjusted for age, sex, body mass index and type 2 diabetes at baseline.

a,

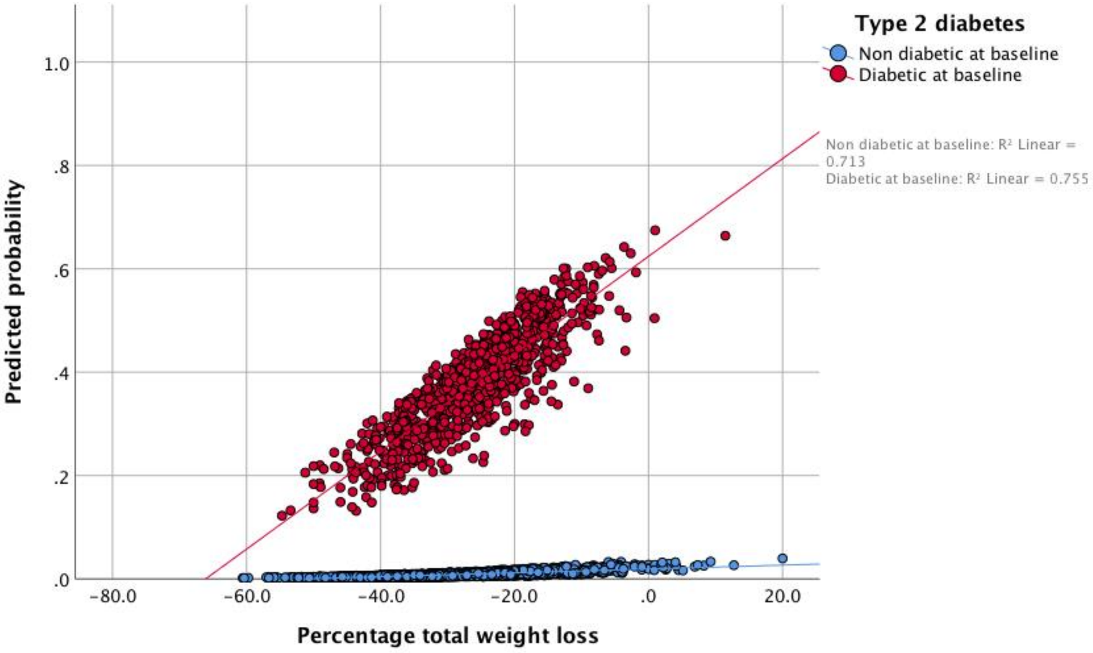

Brissman M, *et al. BMJ Open* 2022; 11:e046407. doi: 10.1136/bmjopen-2020-046407

b,

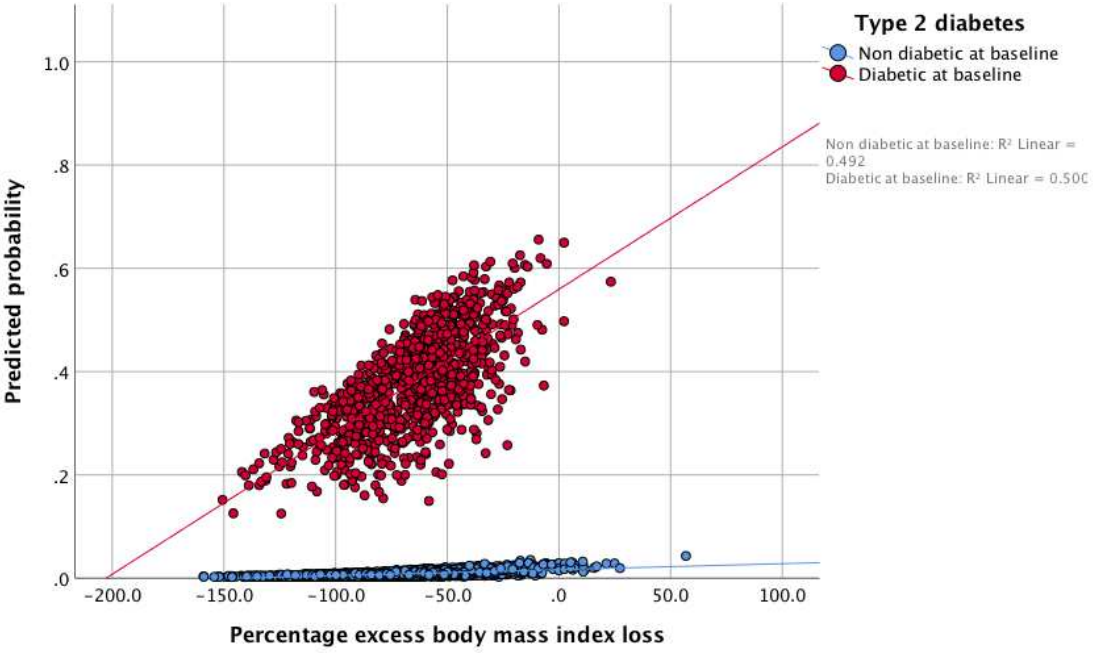
Type 2 diabetes
Non diabetic at baseline
Diabetic at baseline
Non diabetic at baseline: R² Linear = 0.492
Diabetic at baseline: R² Linear = 0.500
Predicted probability
Percentage excess body mass index loss
1.0
.8
.6
.4
.2
.0
-200.0
-150.0
-100.0
-50.0
.0
50.0
100.0

c,

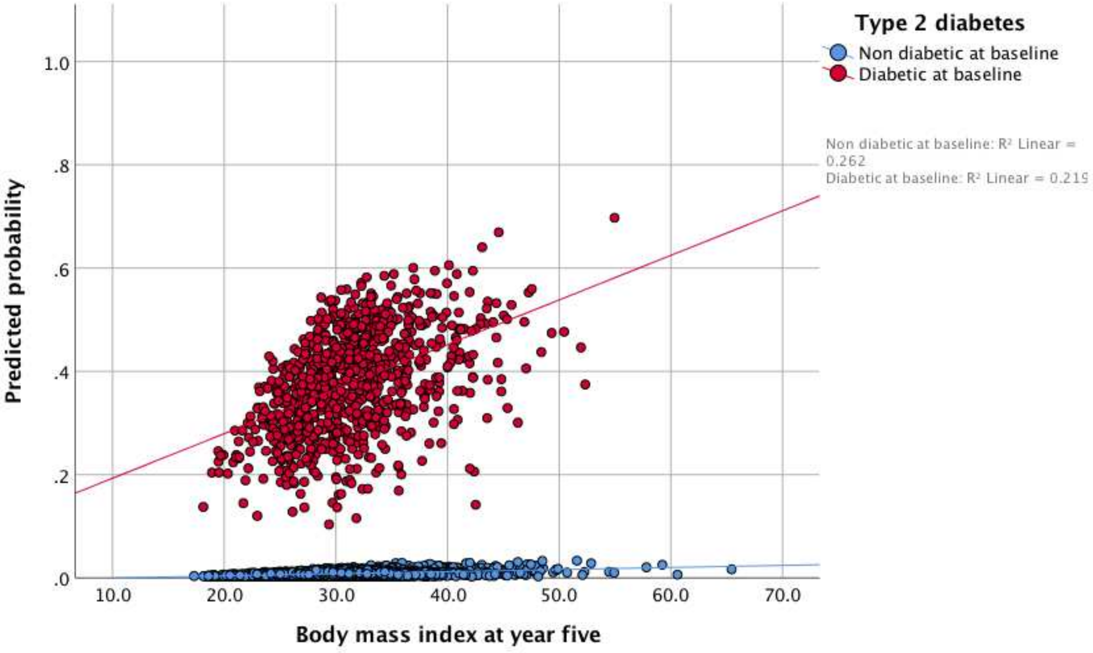
Type 2 diabetes
Non diabetic at baseline
Diabetic at baseline
Non diabetic at baseline: R² Linear = 0.262
Diabetic at baseline: R² Linear = 0.219
Predicted probability
Body mass index at year five
1.0
.8
.6
.4
.2
.0
10.0
20.0
30.0
40.0
50.0
60.0
70.0

eFigure 4.a-c .Predicted probability of dyslipidemia at year five after surgery plotted over a, percentage weight loss from baseline to year five, b, excess body mass index loss from baseline to year five, c, body mass index at year five. Groups based on presence of dyslipidemia at baseline. Adjusted for age, sex, baseline body mass index and dyslipidemia at baseline.

a,

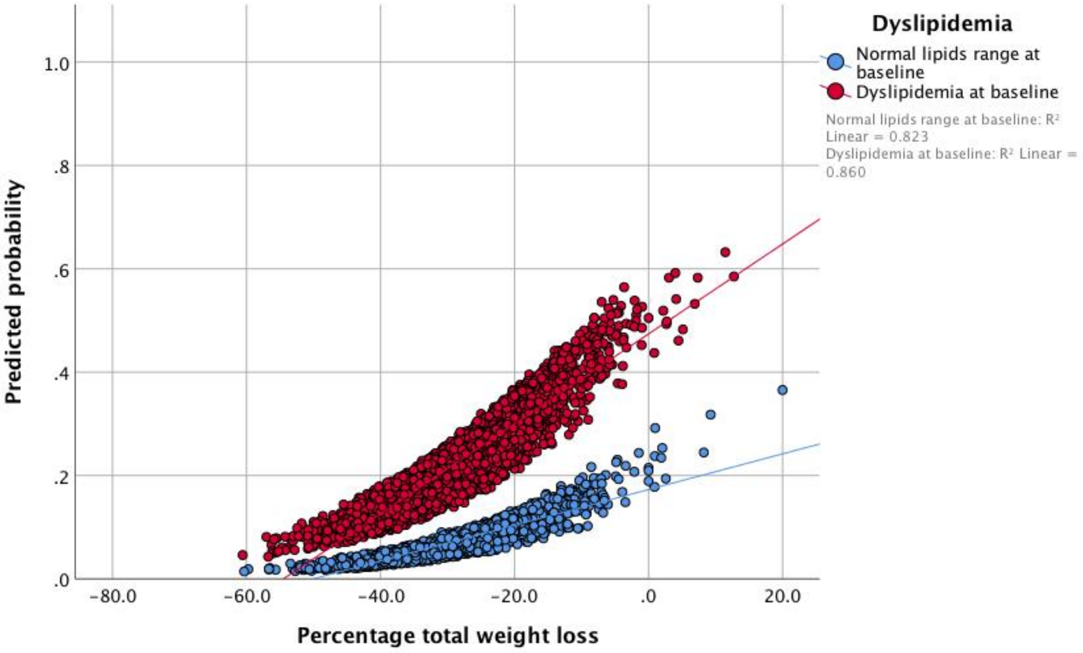

Brissman M, *et al. BMJ Open* 2022; 11:e046407. doi: 10.1136/bmjopen-2020-046407

b,

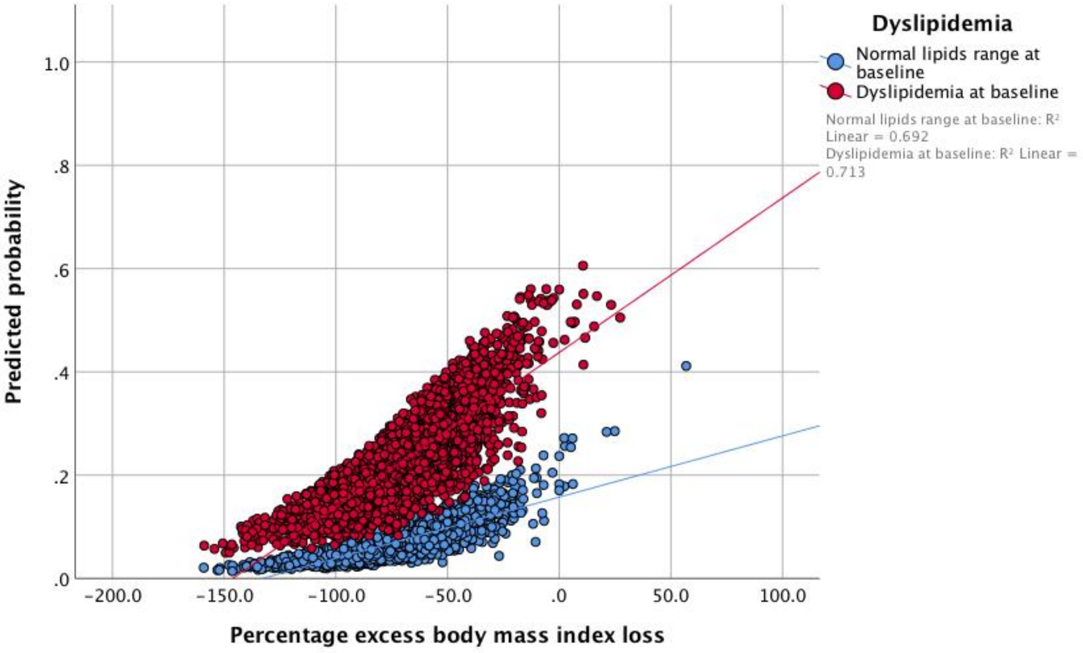
Dyslipidemia
Normal lipids range at baseline
Dyslipidemia at baseline
Normal lipids range at baseline: R² Linear = 0.692
Dyslipidemia at baseline: R² Linear = 0.713
Predicted probability
Percentage excess body mass index loss
1.0
.8
.6
.4
.2
.0
−200.0
−150.0
−100.0
−50.0
.0
50.0
100.0

c,

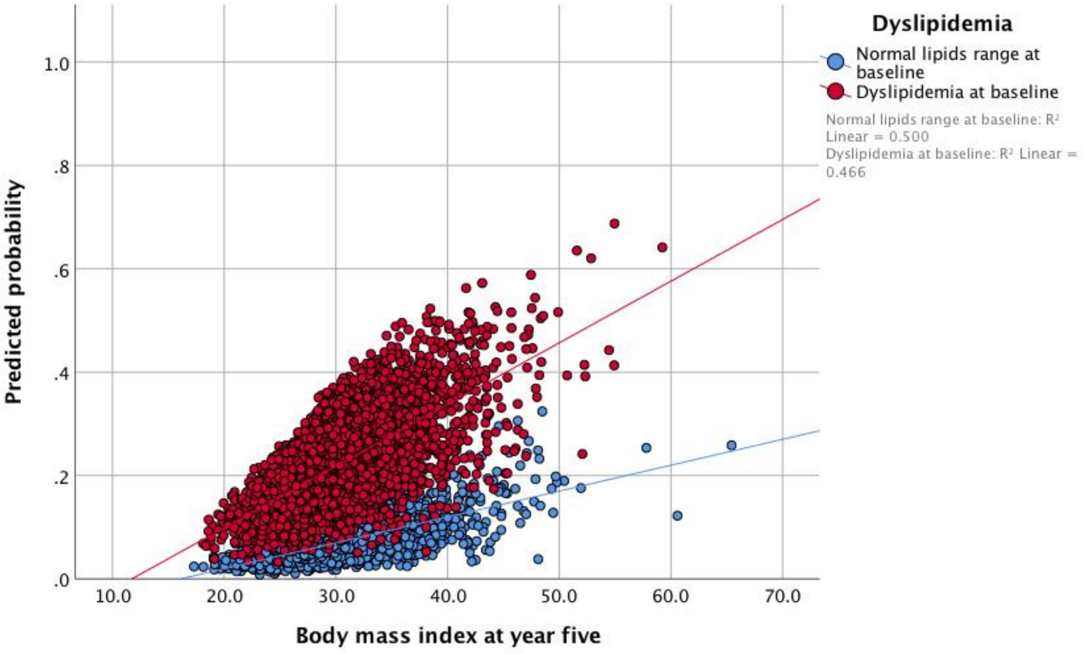
Dyslipidemia
Normal lipids range at baseline
Dyslipidemia at baseline
Normal lipids range at baseline: R² Linear = 0.500
Dyslipidemia at baseline: R² Linear = 0.466
Predicted probability
Body mass index at year five
1.0
.8
.6
.4
.2
.0
10.0
20.0
30.0
40.0
50.0
60.0
70.0

eFigure 5.a-c. Predicted probability of Hypertension at year five after surgery plotted over a, percentage weight loss from baseline to year five, b, excess body mass index loss from baseline to year five, c, body mass index at year five. Groups based on presence of hypertension at baseline. Adjusted for age, sex, body mass index and hypertension at baseline.

a,

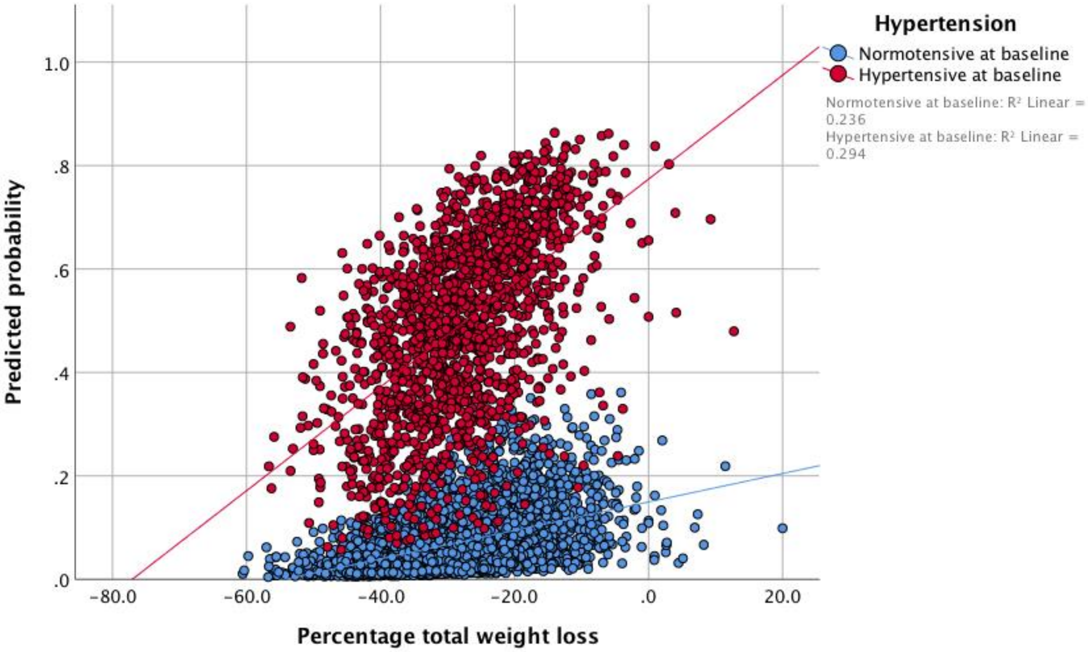

Brissman M, *et al. BMJ Open* 2022; 11:e046407. doi: 10.1136/bmjopen-2020-046407

b,

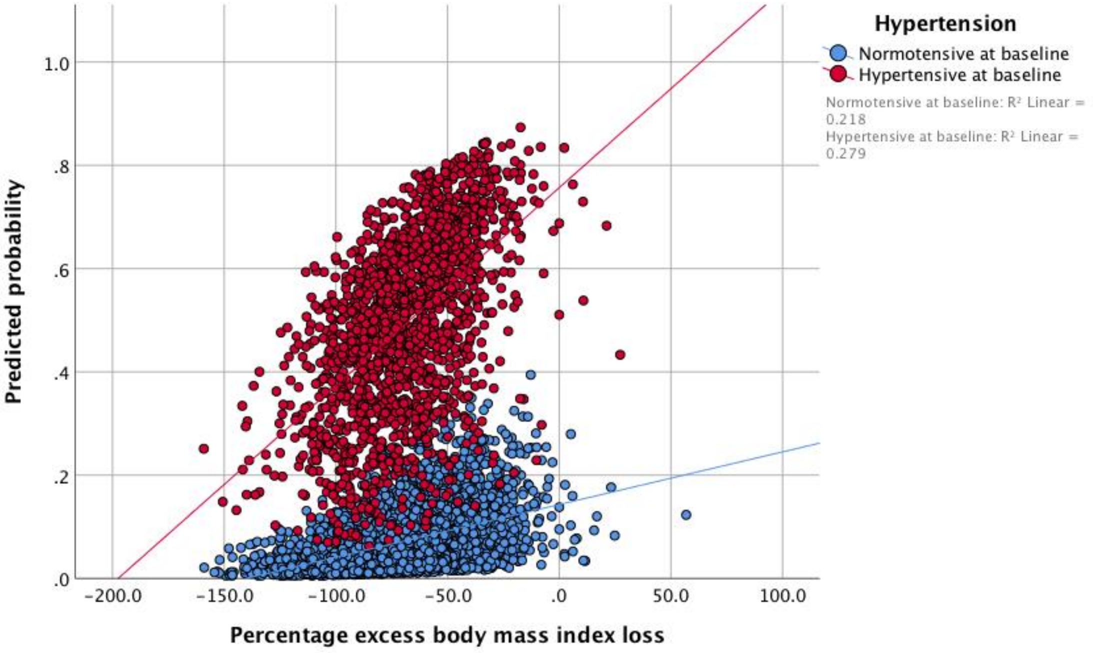

c,

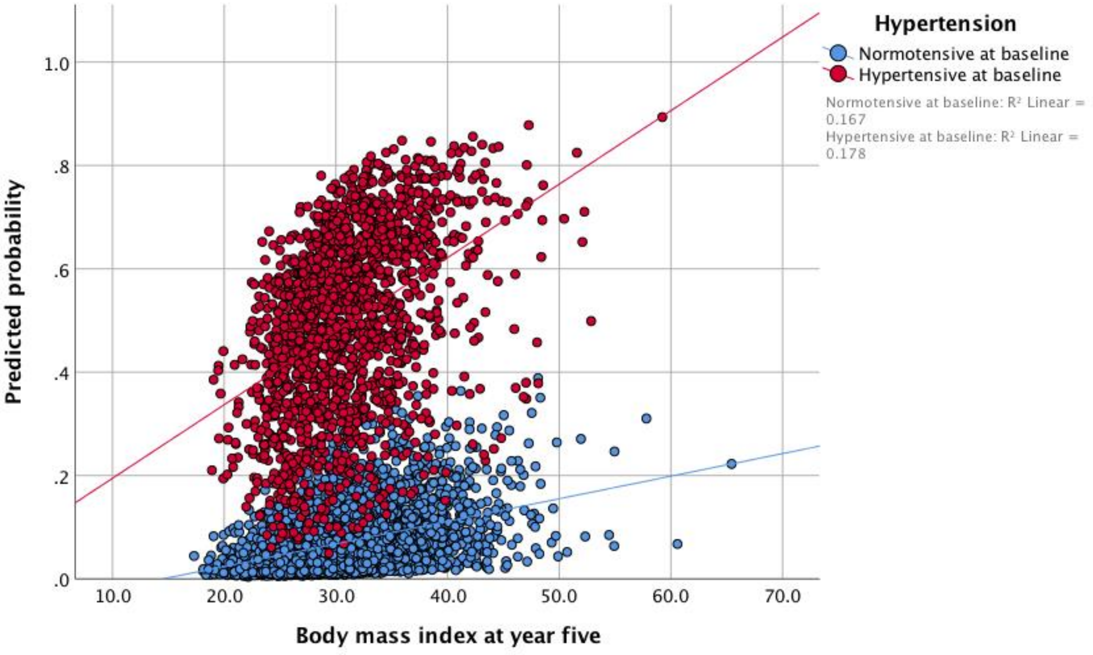

Brissman M, *et al. BMJ Open* 2022; 11:e046407. doi: 10.1136/bmjopen-2020-046407

eTable 1. Modality of the one-, two-, and five year follow-ups

	**Year one** n (%)	**Year two** n (%)	**Year five** n (%)
Clinical visit	4636 (78·1)	3543 (60·0)	2651 (44·7)
Telephone consultation	729 (12·3)	1172 (19·7)	2170 (36·6)
E-mail/letter	217 (3·7%)	458 (7·7)	1087 (18·3)
Unspecified	14 (0·2)	23 (0·4)	28 (0·5)
Missing	340 (5·7)	740 (12·5)	0 (0·0)

Brissman M, *et al. BMJ Open* 2022; 11:e046407. doi: 10.1136/bmjopen-2020-046407

eTable 2. Comparison of baseline characteristics between included participants and those lost to follow-up at year five

	Included		Lost to follow-up		
	n	mean (sd)	n	mean (sd)	P
Age at surgery	5936	39.4 (9.0)	2375	37.6 (9.1)	<0.0001
Sex, % female	5936	79.10%	2375	73.6%	<0.0001
Height, cm	5936	168.8 (8.9)	2375	169.8 (9.3)	<0.0001
Weight, kg	5936	122.8 (20.0)	2375	126.3 (20.7)	<0.0001
BMI at surgery, kg/m^2	5936	42.9 (5.1)	2375	43.6 (5.2)	<0.0001
Glucose, mmol/l	2861	5.9 (1.9)	1312	5.8 (1.8)	0.223
HbA1c, mmol/mol	4168	40.6 (11.4)	1846	40.8 (11.7)	0.528
Pharmacological diabetes treatment	5936	11.4%	2375	9.6%	0.022
Diabetes type 2[A]	5936	15.1%	2375	14.6%	0.543
High-density lipoprotein, mmol/l	4188	1.2 (0.4)	1845	1.1 (0.5)	0.017
Low-density lipoprotein, mmol/l	4110	3.1 (0.9)	1798	3.1 (1.0)	0.756
Triglycerides, mmol/l	4314	1.7 (1.4)	1883	1.8 (2.2)	0.087
Pharmacological dyslipidemia treatment	5936	7%	2375	6.5%	0.465
Dyslipidemia[B]	5936	67.5%	2375	74.1%	<0.0001
Systolic BP, mmHg	2960	133 (16)	1348	135 (17)	0.015
Diastolic BP, mmHg	2960	83 (10)	1347	83 (10)	0.164
Pharmacological hypertension treatment	5936	19.5%	2375	18.1%	0.142
Hypertension[C]	5936	28.4%	2375	29.3%	0.365

BMI – Body mass index

[A] Pharmacologically treated T2D | fasting glucose >7.0mmol/l | HbA1c >48mmol/mol
[B] Pharmacologically treated dyslipidemia | LDL >4.1 | TG > 2.0 | HDL <1.0mmol/L for males and <1.3mmol/L for females
[C] Pharmacologically treated blood pressure | systolic- >140mm/Hg or diastolic blood pressure >90mm/Hg

Brissman M, *et al. BMJ Open* 2022; 11:e046407. doi: 10.1136/bmjopen-2020-046407

eTable 3. Percentage of the 5936 participants with available data on pharmacological treatment and on biochemistry and blood pressure at baseline, one, two and five years after surgery.

	Baseline	Year one	Year two	Year five
Pharmacological treatment				
Type 2 diabetes	100%	94%	88%	100%
Dyslipidemia	100%	94%	88%	100%
Hypertension	100%	94%	88%	100%
Biochemistry				
Low-density lipoprotein	69%	61%	54%	55%
High-density lipoprotein	71%	62%	54%	55%
Triglycerides	73%	63%	54%	55%
Fasting glucose	48%	49%	51%	56%
HbA1c	70%	66%	58%	57%
Blood pressure	50%	37%	35%	34%

Brissman M, *et al. BMJ Open* 2022; 11:e046407. doi: 10.1136/bmjopen-2020-046407

eTable 4. Comparison of baseline characteristics between surgical treatment failure (STF) and non-STF

	STF		Non-STF		
	n	mean (sd)	n	mean (sd)	P
Age at surgery	1371	40.5 (8.8)	4565	39.1 (9.0)	<0.001
Sex, % female	1371	71.4%	4565	81.4%	<0.001
Height, cm	1371	169.6 (9.4)	4565	168.6 (8.7)	<0.001
Weight, kg	1371	128.2 (22.9)	4565	121.2 (18.7)	<0.001
BMI at surgery, kg/m^2	1371	44.4 (6.1)	4565	42.5 (4.6)	<0.001
**Glucose Metabolism**					
Glucose, mmol/l	686	6.2 (2.3)	2175	5.8 (1.8)	<0.001
HbA1c, mmol/mol	1020	42.6 (13.3)	3148	40.0 (10.7)	<0.001
Pharmacological Diabetes treatment	1371	17.4%	4565	9.6%	<0.001
Diabetes type 2[A]	1371	22.0%	4565	13.0%	<0.001
**Lipids**					
High-density lipoprotein, mmol/l	1023	1.1 (0.5)	3165	1.2 (0.4)	0.605
Low-density lipoprotein, mmol/l	1004	3.1 (0.9)	3106	3.1 (0.9)	0.201
Triglycerides, mmol/l	1052	1.7 (1.2)	3262	1.7 (1.4)	0.285
Pharmacological dyslipidemia treatment	1371	9.6%	4565	6.2%	<0.001
Dyslipidemia[B]	1371	72.3%	4565	66.0%	<0.001
**Physiology**					
Systolic BP, mmHg	687	135 (16)	2274	133 (16)	0.001
Diastolic BP, mmHg	686	83 (11)	2274	83 (10)	0.509
Pharmacological hypertension treatment	1371	24.8%	4565	17.9%	<0.001
Hypertension[C]	1371	34.2%	4565	26.6%	<0.001

[A] Pharmacologically treated T2D | fasting glucose >7.0mmol/l | HbA1c >48mmol/mol
[B] Pharmacologically treated dyslipidemia | LDL >4.1 | TG > 2.0 | HDL <1.0mmol/L for males and <1.3mmol/L for females
[C] Pharmacologically treated blood pressure | systolic- >140mm/Hg or diastolic blood pressure >90mm/Hg

Brissman M, *et al. BMJ Open* 2022; 11:e046407. doi: 10.1136/bmjopen-2020-046407

eTable 5. Odds of cardiometabolic disease at year five, separate models for each definitions of surgical treatment failure. Adjusted for sex and baseline; age, BMI and corresponding cardiometabolic disease.

	Beta	Standard error	Odds ratio	95% Confidence interval		p
				Lower	Upper	
**Type 2 diabetes**						
Non surgical treatment failure (ref)						
Total weight loss <20%	0.818	0.142	2.266	1.715	2.995	<0.001
Excess BMI loss <50%	0.760	0.144	2.138	1.611	2.837	<0.001
BMI >35 or >40*	0.893	0.184	2.441	1.703	3.499	<0.001
Compiled**	0.743	0.137	2.102	1.608	2.749	<0.001
**Dyslipidemia**						
Non surgical treatment failure (ref)						
Total weight loss <20%	0.946	0.084	2.574	2.185	3.033	<0.001
Excess BMI loss <50%	0.935	0.083	2.548	2.164	3.000	<0.001
BMI >35 or >40*	0.863	0.100	2.370	1.949	2.883	<0.001
Compiled**	0.916	0.079	2.500	2.143	2.918	<0.001
**Hypertension**						
Non surgical treatment failure (ref)						
Total weight loss <20%	0.687	0.098	1.988	1.642	2.407	<0.001
Excess BMI loss <50%	0.652	0.095	1.920	1.593	2.315	<0.001
BMI >35 or >40*	0.569	0.114	1.767	1.413	2.210	<0.001
Compiled**	0.616	0.089	1.851	1.554	2.206	<0.001

*For subjects with presurgery BMI of <50 and >50, respectively.
**Defined as meeting any of the definitions, <20%TWL | <50%EBMIL | BMI >35 or >40.

eTable 6. Odds of Cardiometabolic disease at year five for subjects reaching one, two or three of the three definitions (exclusively, subjects may only be in one group). Adjusted for sex and baseline; age, BMI and corresponding cardiometabolic disease.

				95% Confidence Interval		
	Beta	Standard error	Odds ratio	Lower	Upper	p
**Type 2 diabetes**						
Non surgical treatment failure (ref)						
Surgical failure 1/3	0.409	0.258	1.506	0.908	2.496	0.113
Surgical failure 2/3	0.592	0.186	1.808	1.255	2.606	0.001
Surgical failure 3/3	1.119	0.198	3.061	2.078	4.509	<0.001
**Dyslipidemia**						
Non surgical treatment failure (ref)						
Surgical failure 1/3	0.605	0.147	1.832	1.373	2.445	<0.001
Surgical failure 2/3	0.961	0.111	2.614	2.103	3.250	<0.001
Surgical failure 3/3	1.050	0.111	2.857	2.299	3.550	<0.001
**Hypertension**						
Non surgical treatment failure (ref)						
Surgical failure 1/3	0.415	0.165	1.515	1.097	2.091	0.012
Surgical failure 2/3	0.556	0.130	1.744	1.352	2.248	<0.001
Surgical failure 3/3	0.798	0.129	2.221	1.724	2.862	<0.001

Brissman M, *et al. BMJ Open* 2022; 11:e046407. doi: 10.1136/bmjopen-2020-046407
