# Supplementary material for: Prevalence of insufficient weight loss 5 years after Roux-en-Y gastric bypass: metabolic consequences and prediction estimates: a prospective registry study
Source: BMJ Open. 2021 Mar 2;11(3):e046407. doi: 10.1136/bmjopen-2020-046407 (PMC7929824; doi:10.1136/bmjopen-2020-046407)
Supplement: Author's manuscript [file bmjopen-2020-046407.draft_revisions.pdf]

BMJ Open

BMJ Open is committed to open peer review. As part of this commitment we make the peer review history of every article we publish publicly available.

When an article is published we post the peer reviewers' comments and the authors' responses online. We also post the versions of the paper that were used during peer review. These are the versions that the peer review comments apply to.

The versions of the paper that follow are the versions that were submitted during the peer review process. They are not the versions of record or the final published versions. They should not be cited or distributed as the published version of this manuscript.

BMJ Open is an open access journal and the full, final, typeset and author-corrected version of record of the manuscript is available on our site with no access controls, subscription charges or pay-per-view fees (http://bmjopen.bmj.com).

If you have any questions on BMJ Open's open peer review process please email info.bmjopen@bmj.com

BMJ Open

# Insufficient weight loss five years after Roux-en-Y gastric bypass: Prevalence, metabolic consequences and prediction estimates -A prospective registry study

| | |
|---|---|
| Journal: | *BMJ Open* |
| Manuscript ID | bmjopen-2020-046407 |
| Article Type: | Original research |
| Date Submitted by the Author: | 28-Oct-2020 |
| Complete List of Authors: | Brissman, Markus; KI CLINTEC, Department of Clinical Science, Intervention and Technology, Division of Pediatrics; Karolinska University Hospital, Allied Health Professionals Function, Occupational Therapy & Physiotherapy<br>Beamish, Andrew; University of Gothenburg Institute of Clinical Sciences, Department of Surgical Research; Swansea University Medical School<br>Olbers, Torsten; Linkoping University Department of Biomedical and Clinical Sciences, Department of Biomedical and Clinical Sciences and Wallenberg Center for Molecular Medicine; Vrinnevi Hospital in Norrkoping, Department of surgery<br>Marcus, Claude; KI CLINTEC, Department of Clinical Science, Intervention and Technology, Division of Pediatrics, |
| Keywords: | SURGERY, Lipid disorders < DIABETES & ENDOCRINOLOGY, DIABETES & ENDOCRINOLOGY |
| | |

SCHOLARONE™
Manuscripts

*I, the Submitting Author has the right to grant and does grant on behalf of all authors of the Work (as defined in the below author licence), an exclusive licence and/or a non-exclusive licence for contributions from authors who are: i) UK Crown employees; ii) where BMJ has agreed a CC-BY licence shall apply, and/or iii) in accordance with the terms applicable for US Federal Government officers or employees acting as part of their official duties; on a worldwide, perpetual, irrevocable, royalty-free basis to BMJ Publishing Group Ltd ("BMJ") its licensees and where the relevant Journal is co-owned by BMJ to the co-owners of the Journal, to publish the Work in this journal and any other BMJ products and to exploit all rights, as set out in our licence.*

*The Submitting Author accepts and understands that any supply made under these terms is made by BMJ to the Submitting Author unless you are acting as an employee on behalf of your employer or a postgraduate student of an affiliated institution which is paying any applicable article publishing charge ("APC") for Open Access articles. Where the Submitting Author wishes to make the Work available on an Open Access basis (and intends to pay the relevant APC), the terms of reuse of such Open Access shall be governed by a Creative Commons licence – details of these licences and which Creative Commons licence will apply to this Work are set out in our licence referred to above.*

*Other than as permitted in any relevant BMJ Author's Self Archiving Policies, I confirm this Work has not been accepted for publication elsewhere, is not being considered for publication elsewhere and does not duplicate material already published. I confirm all authors consent to publication of this Work and authorise the granting of this licence.*

For peer review only

# Insufficient weight loss five years after Roux-en-Y gastric bypass: Prevalence, metabolic consequences and prediction estimates
# -A prospective registry study

Brissman, M. 1,2, Beamish, A. J. 3,4, Olbers, T. 5,6, Marcus, C. 1

1. Department of Clinical Science, Intervention and Technology, Division of Pediatrics, Karolinska Institutet, Huddinge, Sweden
2. Allied Health Professionals Function, Occupational Therapy & Physiotherapy, Karolinska University Hospital, Stockholm, Sweden
3. Department of Surgical Research, Institute of Clinical Sciences, Gothenburg University, Gothenburg, Sweden.
4. Swansea University Medical School, Swansea, United Kingdom.
5. Department of Biomedical and Clinical Sciences and Wallenberg Center for Molecular Medicine, Linköping University, Linköping, Sweden.
6. Department of Surgery, Vrinnevi hospital, Norrköping, Sweden

Corresponding author: Markus Brissman, Blickagången 6A, 141 57 Huddinge, Sweden.
Markus.brissman@ki.se

Word Count: 3249

Key words: Bariatric surgery, treatment failure, obesity-related comorbidity, prediction algorithms

For peer review only

Strengths and limitations of this study

- A large prospective cohort of nearly 6000 patients from bariatric surgery centers with a minimum of 60% retention rate at year five after bariatric surgery.
- Pre-defined thresholds of surgical treatment failure and cardiometabolic health were applied.
- The prediction model of surgical treatment failure was cross-validated using partial data, however, further validation of an unrelated cohort is preferable.
- Data originates from the whole of Sweden; thus generalizability may be limited to countries with similar ethnic diversity.

For peer review only


For peer review only - http://bmjopen.bmj.com/site/about/guidelines.xhtml

Abstract

**Objective:** The study aimed to investigate the heterogeneity of weight loss five years after RYGB and the association with cardiometabolic health.

**Design:** Retrospective analysis of prospectively collected data from the Scandinavian Obesity Surgery Registry (SOReg).

**Setting:** 29 surgical units from the whole of Sweden contributed data. Inclusion was restricted to surgical units with a retention rate of >60% five years post-surgery.

**Participants:** 10633 patients were extracted from SOReg. In total 5936 participants were included in the final sample, 79·1% females. The mean age of participants before surgery was 39·4±9 years and mean body mass index (BMI) 42·9±5·1. 2322 were excluded (death before the 5-year follow-up (n=148), other types of surgery or reoperations (n=637), age at surgery <18 or >55 years (n=1329), pre-surgery BMI <35kg/m$^2$ (n=208)). In total 2375 (29%) of eligible individuals were lost to the 5-year follow-up.

**Main Outcome:** The occurrence of surgical treatment failure five years after surgery was based on the three previously published definitions: percent excess BMI loss <50%, total weight loss <20%, or BMI >35 where initial BMI was <50, or >40 where initial BMI was >50. In addition, we report the association between surgical treatment failure and biochemical markers of obesity-



For peer review only - http://bmjopen.bmj.com/site/about/guidelines.xhtml

For peer review only

related comorbidity. We also developed predictive models to identify patients with a high risk of surgical treatment failure five years post-surgery.

**Results:** In total, 23·1% met at least one definition of surgical treatment failure at year five which was associated with (adjusted odds ratio [OR] with 95% confidence interval [95%CI]): Type 2 diabetes (T2D, OR=2·1; 95%CI 1·6 to 2·7), dyslipidemia (OR=1·8; 95%CI 1·6 to 2·1), and hypertension (OR=1·9; 95%CI 1·6 to 2·2). Surgical treatment failure at five years was predicted by combined demographic and anthropometric measures from baseline, one and two years post-surgery (area under the curve=0·874).

**Conclusion:** LRYGB leads to a marked and sustained weight loss with improvement of obesity-related comorbidity in most patients. However, 23% met at least one definition of surgical treatment failure, which was associated with a greater risk of relapse and a higher incidence of T2D, dyslipidemia and hypertension five years after surgery. Poor Initial weight loss and early weight regain are strong predictors of long-term treatment failure and may be used for early identification of patients who require additional weight loss support.


For peer review only - http://bmjopen.bmj.com/site/about/guidelines.xhtml

## Introduction

Obesity is a heterogeneous disease [1] associated with several comorbid conditions, which ultimately increases the risk of all-cause mortality [2]. Bariatric surgery is the most effective treatment for severe obesity. Long-term follow-up studies of Roux-en-Y Gastric Bypass (RYGB) show excellent results at the group level in reductions in weight, morbidity, and mortality compared with non-surgical treatment [3-5]. In Sweden, approximately 5500 bariatric operations are performed annually and, until 2014, the technique was almost exclusively RYGB [6].

Weight loss after surgery is typically achieved during the first and second year, followed by weight maintenance or moderate regain 5-10 years after surgery [7]. However, despite good overall results, the response and durability of surgically induced weight loss are heterogenous [8-10], and surgical treatment failure has been recognized as a potential clinical problem [11-13].

The prevalence of surgical treatment failure is unclear, largely because an all-encompassing, unambiguous definition remains elusive [11-14]. In a landmark controlled study by Adams et al. [5], based on 418 RYGB patients, 30% of participants experienced <20% of total body weight loss at 12 years after RYGB.

It is still unclear to which extent cardiometabolic improvements after bariatric surgery depends on the degree of weight loss. Long-term studies have reported temporally declining rates of remission from obesity-related comorbidities [5,15] and the rate of relapse, especially for type 2


For peer review only - http://bmjopen.bmj.com/site/about/guidelines.xhtml

diabetes (T2D), has rather been attributed to pre-surgery disease duration and progression than to insufficient weight loss [5,16]. Although an association between T2D relapse and weight regain has been suggested in some studies [17-19], others have not found any association between the degree of long-term weight loss and cardiometabolic outcome [20-22]. The annual summary of the Scandinavian Obesity Surgery Registry (SOReg) recently described an association between baseline T2D and inadequate post-operative weight loss [23].

In this study, based on a large cohort of patients prospectively collected in SOReg [6], we report on the heterogeneity of weight loss outcome, focusing primarily on the occurrence of surgical treatment failure five years after surgery, according to any of three published definitions. We also report the association between surgical treatment failure and cardiometabolic disease and we present predictions of surgical treatment failure based on background data and weight development during the first two years after RYGB.

## Methods

### Data Source

The data source for this study was SOReg, a Swedish nationwide registry that began collecting data in 2007; from 2011, the registry covered 95-99% of all bariatric surgery performed in Sweden. Data were retrieved in accordance with the study protocol. For this retrospective analysis, data were requested for all patients from surgical units and yearly cohorts that had a


For peer review only - http://bmjopen.bmj.com/site/about/guidelines.xhtml

five-year retention rate of ≥60%. Data covered demographics, anthropometrics, pharmacological treatment, obesity-related comorbidity, biochemical markers and blood pressure at four time points: before surgery (baseline), and at one, two and five years after surgery.

Participants

In total, 29 surgical units contributed data to the study through the SOReg database, ranging in number from 1 to 1643 patients, and data on 10633 unique patients were extracted. Exclusion was performed in iteration steps and a total of 4697 patients were excluded. Of the participants included in this study, 84·3% had BMI measurements available at all time points. Missing data totaled 13·2% at either the one- or two-year follow-up, and 2·5% at both the one- and two-year follow-ups. The follow-up modality was a clinical visit, telephone consultation, e-mail/letter, or unspecified. Figure 1 shows the flowchart of the study participants.

Loss to follow-up analysis

A comparison of baseline characteristics between the study participants and those lost to follow-up revealed that lost participants had a younger age, a higher BMI and a male predomination. A detailed comparison appears in eTable 1 in the Supplement.

## Definitions



For peer review only - http://bmjopen.bmj.com/site/about/guidelines.xhtml

Surgical treatment failure

Surgical treatment failure was assessed and defined as meeting at least one of three definitions five years after surgery: i) <50% excess BMI loss (%EBMIL), ii) <20% total weight loss (%TWL), and iii) BMI >35 kg/m$^2$ where baseline was <50 kg/m$^2$, or >40 kg/m$^2$ if baseline BMI was >50 kg/m$^2$. These definitions have been used elsewhere [11,24] and, taken together, provide a means to define failure for patients within different weight categories.

%EBMIL was calculated as ((baseline BMI – year five BMI)/(baseline BMI - 25))*100

%TWL was calculated as ((baseline BMI – year five BMI)/baseline BMI)*100

Two trajectories - inadequate weight loss and weight regain - can be defined that lead to long-term surgical treatment failure. Inadequate weight loss has been quantified during the first 6-12 months after surgery [25], and weight regain has typically been described as an increase above a specified threshold [12,13].

In this paper inadequate weight loss was defined as <25%TWL from baseline to one year post-surgery, similar to the 25$^{th}$ percentile presented by Manning et al. [25].

Early weight regain was defined as any absolute weight gain, expressed in kilograms, occurring between year one and two after surgery. This definition generated two groups. Long-term weight regain, defined according to Odom et al. [26] in three groups: >15% regain of BMI nadir, 0·1-15% regain of BMI nadir, and no weight regain, to five years post-surgery. These definitions were used to capture early weight regain as a predictive measurement of long-term surgical


For peer review only - http://bmjopen.bmj.com/site/about/guidelines.xhtml

treatment failure, and to differentiate between the normally occurring fluctuation of body weight in the maintenance phase and the potentially harmful weight regain previously suggested [18,19].

For calculations, BMI nadir was accepted as the lowest measured weight at either the one or two year follow-up. In the case of missing data from one of those time points the observed measurement was taken as the nadir.

## Obesity-related comorbidities and metabolic markers

It is mandatory to report obesity-related comorbidities (e.g., T2D, dyslipidemia, hypertension) [6] requiring pharmacological treatment in SOReg, and data were available for 88-100% included individuals depending on timepoint (full description in eTable 2 in the Supplement).

Blood pressure and biochemical markers, such as low-density lipoprotein (LDL), high-density lipoprotein (HDL), triglycerides (TG), fasting glucose, and glycated hemoglobin (HbA1c), are optional to report. Data were available data from 34-73% included participants (eTable 2 in the Supplement).

Changes in blood pressure and biochemical markers were compared, stratified by surgical treatment failure at the five-year follow-up, and by pharmacological treatment at baseline. Additionally a broader classification of disease traits was generated, similar to that previously described [5,27], by compiling a disease-specific biochemical marker above a cut-off (eAppendix 1


For peer review only - http://bmjopen.bmj.com/site/about/guidelines.xhtml

in the Supplement., in combination with pharmacological treatment. This classification was applied at all time points and used to assess prevalence and change over time. Thus, six groups were generated: participants without disease traits at baseline were classified “disease-free” if no disease trait was evident at any time point, “intermittent” if disease-free at both baseline and five-year follow-up, but not in between, and “incidence” where a disease trait developed during the five-year follow-up period. Participants with a disease trait at baseline were classified “remission” if no disease trait was evident at five-year follow-up, “relapse” if disease-free at year one, two, or both, but not at year five, and “no remission” where at least one disease trait was evident at all time points.

For clarity, the compiled disease traits are hereafter referred to as T2D, dyslipidemia and hypertension.

## Statistics

All statistical analyses were performed using SPSS v.24 (IBM Corp. USA) and STATA IC 15.1 (StataCorp USA). Descriptive statistics are presented as mean ± standard deviation (±sd), or as a percentage (%), unless otherwise specified.

Characteristics were compared between those lost to follow-up (eTable 1 in the Supplement) and those included in the analysis, as well as according to surgical treatment failure status (eTable 3 in the Supplement), using independent t- and chi-square tests.

We described the prevalence and change in cardiometabolic disease and assessed the odds associated with surgical treatment failure using logistic regression, first using a crude model


For peer review only - http://bmjopen.bmj.com/site/about/guidelines.xhtml

(data not shown) and then multivariable models (separate, compiled or additive for each definition of surgical treatment failure) in which we adjusted for sex, age and BMI at baseline, and corresponding cardiometabolic disease. Results are presented as odds ratios (OR) with 95% confidence intervals (95%CI).

In addition, we used logistic regression to predict the probability of meeting at least one definition of surgical treatment failure, which we considered dichotomously (1 = surgical treatment failure, 0 = otherwise). Our predictions used sex, baseline, age, BMI and %TWL for the first year and change in weight (kg) for the second year. We measured performance by calculating the receiver operating characteristic (ROC) curve and the corresponding area under the curve (AUC) and by using cross-validation (leave 10%, k = 10 replicates).

Finally, several sensitivity analyses were undertaken for the primary endpoint (i.e. surgical treatment failure), which can be found in eAppendix 2 in the Supplement.

The significance level was set to 0·05 for all analyses (two-tailed), and p-values are reported with three decimals.

Patient and public involvement

Patients nor the public were involved in the conduct of this study.

## Results



For peer review only - http://bmjopen.bmj.com/site/about/guidelines.xhtml

In total, 5936 patients (79·1% female), aged 18-55 years, who had undergone LRYGB from 2007 to 2012, were included in the final sample (Figure 1). At baseline, the mean age was 39·4±9·0 years and BMI was 42·9±5·1 kg/m$^2$. Patient characteristics are presented in Table 1. At year five, overall mean BMI was 30·4±5·2, mean weight loss 35·8 ±13·8 kg, BMI loss 12·6±4·7 kg/m$^2$, %EBMIL 72·2±25·2% and %TWL 29·1±9·8%.

Inadequate weight loss (i.e. <25%TWL from baseline to year one) was identified in 17·1% of 5596 participants with available data.

Early weight regain (between year one and two) was identified in 38·7% of 5010 participants with available data, with a mean increase of 4·5±3·9 kg (range 1 to 38 kg), compared with a mean decrease of 4·4±5·1 kg (range 66 to 0 kg) in the no regain group.

Long-term weight change between nadir and five year follow-up was distributed as follows: >15% regain (+17·7±7·2 kg, range 7 to 101 kg) in 19·9% of participants, 0·1-15% regain (+5·7±3·5 kg, range 0 to 19 kg) in 59·3%, and no weight regain (-5·0±5·2 kg, range -36 to 0 kg) in 20·8%.

Overall, the prevalence of meeting at least one of the three definitions of surgical treatment failure five years after LRYGB was 23·1% (n=1371). The distribution between the three definitions was 19·2% (n=1138) for <50%EBMIL, 17·0% (n=1010) for <20%TWL, and 14·1% (n=835) for BMI >35 or >40 kg/m$^2$. There was substantial overlap, 39·8% (n=545) meeting all three definitions and 38·1% (n=522) meeting two of the three definitions (Figure 2).


For peer review only - http://bmjopen.bmj.com/site/about/guidelines.xhtml

Surgical treatment failure was more common among patients with inadequate weight loss (60% vs. 15·4%, p<·001) and early weight regain (33·8% vs. 15·6%, p<·001). Comparing long-term weight regain, the proportion meeting criteria for failure was highest in participants with >15% regain from nadir (46·7%), followed by 0·1-15% (21·1%), and no regain (5·1%), (p<·001). Patients with no long-term weight regain but surgical treatment failure had higher baseline BMI (48·5 vs. 43·1, p<·001) and lower %TWL at one- and two-year follow-up (-18·0% vs. -30·5% and -18·1% vs. -32·3%, respectively, both p<·001).

Cardiometabolic disease

Biochemical and physiological measures improved following surgery in participants with and without surgical treatment failure. Mean values, stratified by surgical treatment failure and baseline pharmacological treatment, are shown from baseline to year five in eFigures 1.a-g and 2.a-g in the Supplement.

Overall, the prevalence of cardiometabolic disease decreased from baseline to 5 years: T2D from 15·1% (n=896) to 6·4% (n=380), dyslipidemia from 60·7% (n=3603) to 16·4% (n=974), and hypertension from 28·4% (n=1683) to 18·9% (n=1124). The rates of being disease-free, incident and intermittent disease, as well as remission, relapse and no remission, varied between surgical and non-surgical treatment failure (Table 2).

Logistic regression (adjusted for sex, age, BMI, and corresponding cardiometabolic disease at baseline) confirmed an association between surgical treatment failure and cardiometabolic


For peer review only - http://bmjopen.bmj.com/site/about/guidelines.xhtml

disease at year five: T2D, OR=2·10 (95%CI 1·61 to 2·75); dyslipidemia, OR=2·50 (95%CI 2·14 to 2·92); and hypertension, OR=1·85 (95%CI 1·55 to 2·21). Individual definitions were similarly associated with cardiometabolic disease (eTable 4 in the Supplement). The combined effect of fulfilling one, two, or three of the definitions is presented in eTable 5 in the Supplement. Predicted probability of cardiometabolic disease plotted against continuous %EBMIL, %TWL, and BMI at year five is illustrated in eFigures 3-5.a-c in the Supplement.

Inadequate weight loss during year one was significantly associated with T2D (OR=1·84; 95%CI 1·38 to 2·45), dyslipidemia (OR=1·89; 95%CI 1·59 to 2·25), and hypertension (OR=1·61; 95%CI 1·32 to 1·96). Late weight regain (≥15% regain from nadir) was significantly associated with dyslipidemia (OR=1·64; 95%CI 1·31 to 2·05) and hypertension (OR=1·41; 95%CI 1·10 to 1·81), but not T2D (OR=1·25; 95%CI 0·84 to 1·88).

Predicting surgical treatment failure

The estimated regression coefficients and OR are presented in Table 3. Given age, sex and baseline BMI and %TWL from baseline to the one-year follow-up, and change in weight (kg) between one- and two-year follow-up, the predicted probability of surgical treatment failure five years after surgery is given by:

P(surgical treatment failure) = exp(a)/(1+(a)) with a = - 1·1 + 0·00545*(sexFemale) + 0·00299*(age at surgery) + 0·14949*(baseline BMI) + 0·22310*(%TWL year one) + 0·15982*(weight change year one to year two (kg)). Examples of the probability calculation are presented in eAppendix 3 in the Supplement.


For peer review only - http://bmjopen.bmj.com/site/about/guidelines.xhtml

As depicted in Figure 3, this simple model provided a good prediction (AUC = 0·8743).

## Discussion

This analysis of prospectively collected data on 5963 adults who underwent primary LRYGB surgery, revealed that almost one in four participants fulfilled at least one of the three applied definitions of surgical treatment failure, five years after surgery. Surgical treatment failure was associated with a negative effect on cardiometabolic health: lower rate of remission and more frequent relapse and incidence of T2D, dyslipidemia and hypertension. Each definition of surgical treatment failure and weight regain was independently associated with cardiometabolic health. 9.2 percent of these patients fulfilled the criteria for all of the three definitions [11,24] of surgical treatment failure and they provided a very strong association with T2D, dyslipidemia and hypertension.

The extent to which insufficient weight loss and weight regain affect cardiometabolic outcome is unclear, both confirmative [13,18,19,28-30] and negative [20-22,31,32] findings have been reported. In the present study inadequate weight loss during year one and weight regain during year two were investigated. Both were found to be associated with cardiometabolic outcomes, however, both were in the present study viewed as prerequisites for surgical treatment failure, which in turn was associated with a less favorable metabolic profile five years after surgery, regardless



For peer review only - http://bmjopen.bmj.com/site/about/guidelines.xhtml

of whether or not patients were taking T2D, dyslipidemia, or hypertension medications prior to surgery.

Early identification of those with a high risk of long-term surgical treatment failure may facilitate additional weight loss support [33-35]. Unfortunately neither we, nor others, have been able to build a sufficiently reliable model using exclusively pre-surgical characteristics [36]. However, our results indicate that long-term surgical treatment failure can, with good accuracy (AUC = 0·8743), be predicted by sex, age and BMI at baseline, together with %TWL during year one and weight change during year two. We found that %TWL during year one was the strongest predictor of surgical treatment failure. Similarly the initial six month weight loss predicts the 24-month weight loss [25].

The present study terms long-term poor weight loss after surgery as surgical treatment failure. This wording should not be interpreted to mean that the surgical procedure failed, but rather that the therapy alone was insufficient to produce the required degree of long-term weight loss. This reasoning should not be surprising given the heterogeneous nature of obesity, as any standardized treatment is likely to result in a spectrum of outcomes. Despite that, bariatric surgery has remained a stand-alone treatment. This is contrary to bariatric surgery guidelines suggesting active treatment of patients with poor weight outcome [37]. In addition patients have also expressed a need for more extensive follow-up [38]. Recognizing this, bariatric surgery would likely benefit from the application of the multidisciplinary and multimodal approach that has evolved in other fields of disease, such as cancer care, where for decades surgery has been

For peer review only


For peer review only - http://bmjopen.bmj.com/site/about/guidelines.xhtml

integrated into multimodal treatment pathways, alongside chemotherapies and radiation therapies. It has been shown that behavioral support [35] and pharmacological treatment [34] can improve the outcome after surgery, indicating potential for additive, perhaps even synergistic effects of combination therapies. However, as a consequence of the disintegrated follow-up after surgery, it is still unclear to which extent outcome after bariatric surgery can be optimized by means of adjuvant treatment.

Strengths of this study include SOReg's prospective collection of data from the whole of Sweden, with broad national coverage. This was demonstrated by the inclusion of nearly 6000 patients from the database of centers with a ≥60% retention rate five years after LRYGB, providing a large and robust data set permitting subgroup analysis.

There are also some limitations. Although the impact of surgical treatment failure on metabolic health is substantial, it does not account for all comorbidity seen at the five-year follow-up. Other factors, such as disease duration before surgery, are also of importance but such information was not available in this study.

Missing data analysis revealed that rates of surgical treatment failure at year one and two were higher in those lost to follow-up year five. In addition, there was a difference in weight loss between the modes of follow-up, possibly implying bias of self-reported data. Similarly, a statistical limitation of note is that we compiled disease-specific traits where missing data are implicitly treated as zeroes. For example, the estimated effects may be diluted (biased towards zero) because the comparison is actual ones vs. a mixture of zeroes and ones. Thus, both the



For peer review only - http://bmjopen.bmj.com/site/about/guidelines.xhtml

overall prevalence of surgical treatment failure and cardiometabolic disease may be underestimated.

The developed prediction model for long-term surgical treatment failure was cross-validated using partial data and can readily be applied to countries with similar cultural and ethnic settings as in northern Europe. However, further validation of an unrelated cohort is preferable, and further devolvement of the model may be required to encompass ethnic diversity.

Unsuccessful surgical treatment result is difficult to define and a large number of definitions and time points have been used [11-14]. Our results would probably have been slightly modified if we had used other definitions. However, the strong associations between surgical treatment failure, as defined in the present study, and cardiometabolic health may support their clinical usefulness.

## Conclusion

RYGB is associated with improvement of obesity-related comorbidity. However, 23% of the patients developed surgical treatment failure five years after surgery, which was associated with a markedly increased risk of cardiometabolic disease. Initial weight loss and early weight regain were strong predictive markers that can be used for the early identification of patients with a high risk of long-term failure. This study underlines the need for long-term follow-up of patients undergoing bariatric surgery by a multidisciplinary team and improved additional behavioral and pharmacological treatment post-surgery are warranted.


For peer review only - http://bmjopen.bmj.com/site/about/guidelines.xhtml

Author contribution

MB and CM conceptualized the study, MB performed data and statistical analyses and drafted the manuscript. All authors contributed to result interpretation and critically revised and approved the final version of the manuscript. MB and CM had full access to all the data in the study and takes responsibility for the integrity of the data and the accuracy of the data analysis.

Funding

This work was funded by the Swedish order of Freemasons, the Swedish Heart- Lung Foundation, the Samariten foundation for pediatric research and Anna-Lisa and Arne Gustafssons foundation.

Competing interests

TO declares participation in advisory board for J&J and Novo Nordisk and reimbursement for lectures and education activities. All fees to institution.

CM has received research grants from Novo Nordisk, Sigrid THX AB and salaries as medical advisor for Itrim AB and Weight Watchers Int.

MB and AJB declares no conflict of interest.

Disclaimer

The funders of this study had no part in study design, collection, analysis or interpretation of data, nor in the writing of the report or in the decision to submit the paper for publication. The corresponding author had full access to the data and had final responsibility for the decision to submit for publication.

For peer review only

Transparency statement

The lead author (MB) and the guarantor (CM), affirms that the manuscript is an honest, accurate, and transparent account of the study being reported; that no important aspects of the study have been omitted; and that any discrepancies from the study as originally planned have been explained.

Dissemination of the study results to patients and patient organizations is not applicable.

Patient consent for publication

Not required.

Ethical approval

This study was approved by the Stockholm ethical board (2017/1793-31).

Data sharing

No additional data available from the authors. Original data may be requested from the Scandinavian Obesity Surgery Registry (https://www.ucr.uu.se/soreg/in-english)


For peer review only - http://bmjopen.bmj.com/site/about/guidelines.xhtml

Reference


1. Gonzalez-Muniesa P, Martinez-Gonzalez MA, Hu FB, et al. Obesity. *Nat Rev Dis Primers* 2017; **3**: 17034.
2. Aune D, Sen A, Prasad M, et al. BMI and all cause mortality: systematic review and non-linear dose-response meta-analysis of 230 cohort studies with 3.74 million deaths among 30.3 million participants. *BMJ* 2016; **353**: i2156.
3. Sjostrom L. Review of the key results from the Swedish Obese Subjects (SOS) trial - a prospective controlled intervention study of bariatric surgery. *Journal of internal medicine* 2013; **273**(3): 219-34.
4. Gloy VL, Briel M, Bhatt DL, et al. Bariatric surgery versus non-surgical treatment for obesity: a systematic review and meta-analysis of randomised controlled trials. *BMJ* 2013; **347**: f5934.
5. Adams TD, Davidson LE, Litwin SE, et al. Weight and Metabolic Outcomes 12 Years after Gastric Bypass. *N Engl J Med* 2017; **377**(12): 1143-55.
6. Hedenbro JL, Naslund E, Boman L, et al. Formation of the Scandinavian Obesity Surgery Registry, SOReg. *Obes Surg* 2015; **25**(10): 1893-900.
7. Buchwald H, Avidor Y, Braunwald E, et al. Bariatric surgery: a systematic review and meta-analysis. *JAMA* 2004; **292**(14): 1724-37.
8. de Hollanda A, Ruiz T, Jimenez A, Flores L, Lacy A, Vidal J. Patterns of Weight Loss Response Following Gastric Bypass and Sleeve Gastrectomy. *Obes Surg* 2015; **25**(7): 1177-83.
9. Azagury D, Mokhtari TE, Garcia L, et al. Heterogeneity of weight loss after gastric bypass, sleeve gastrectomy, and adjustable gastric banding. *Surgery* 2019; **165**(3): 565-70.
10. Courcoulas AP, King WC, Belle SH, et al. Seven-Year Weight Trajectories and Health Outcomes in the Longitudinal Assessment of Bariatric Surgery (LABS) Study. *JAMA Surg* 2018; **153**(5): 427-34.
11. Mann JP, Jakes AD, Hayden JD, Barth JH. Systematic review of definitions of failure in revisional bariatric surgery. *Obes Surg* 2015; **25**(3): 571-4.
12. Karmali S, Brar B, Shi X, Sharma AM, de Gara C, Birch DW. Weight recidivism post-bariatric surgery: a systematic review. *Obes Surg* 2013; **23**(11): 1922-33.
13. King WC, Hinerman AS, Belle SH, Wahed AS, Courcoulas AP. Comparison of the Performance of Common Measures of Weight Regain After Bariatric Surgery for Association With Clinical Outcomes. *JAMA* 2018; **320**(15): 1560-9.
14. Bonouvrie DS, Uittenbogaart M, Luijten A, van Dielen FMH, Leclercq WKG. Lack of Standard Definitions of Primary and Secondary (Non)responders After Primary Gastric Bypass and Gastric Sleeve: a Systematic Review. *Obes Surg* 2019; **29**(2): 691-7.
15. Sjostrom L, Lindroos AK, Peltonen M, et al. Lifestyle, diabetes, and cardiovascular risk factors 10 years after bariatric surgery. *N Engl J Med* 2004; **351**(26): 2683-93.
16. Pories WJ, Swanson MS, MacDonald KG, et al. Who would have thought it? An operation proves to be the most effective therapy for adult-onset diabetes mellitus. *Annals of surgery* 1995; **222**(3): 339-50; discussion 50-2.
17. Shah M, Simha V, Garg A. Review: long-term impact of bariatric surgery on body weight, comorbidities, and nutritional status. *J Clin Endocrinol Metab* 2006; **91**(11): 4223-31.


For peer review only

18. DiGiorgi M, Rosen DJ, Choi JJ, et al. Re-emergence of diabetes after gastric bypass in patients with mid- to long-term follow-up. *Surg Obes Relat Dis* 2010; **6**(3): 249-53.
19. Laurino Neto RM, Herbella FA, Tauil RM, Silva FS, de Lima SE, Jr. Comorbidities remission after Roux-en-Y Gastric Bypass for morbid obesity is sustained in a long-term follow-up and correlates with weight regain. *Obes Surg* 2012; **22**(10): 1580-5.
20. Hawkins RB, Mehaffey JH, McMurry TL, et al. Clinical significance of failure to lose weight 10 years after roux-en-y gastric bypass. *Surg Obes Relat Dis* 2017; **13**(10): 1710-6.
21. Magno F, Sousa PAM, Rodrigues MP, et al. Long term maintenance of glucose and lipid concentrations after Roux-en-Y gastric bypass. *Arch Endocrinol Metab* 2018; **62**(3): 346-51.
22. Aminian A, Jamal M, Augustin T, et al. Failed Surgical Weight Loss Does Not Necessarily Mean Failed Metabolic Effects. *Diabetes Technol Ther* 2015; **17**(10): 682-4.
23. Registry TSOS. Fördjupningsrapport SOReg 2019, diabetes. 2019. https://www.ucr.uu.se/soreg/arsrapporter (accessed 28th oct 2019).
24. Corcelles R, Boules M, Froylich D, et al. Total Weight Loss as the Outcome Measure of Choice After Roux-en-Y Gastric Bypass. *Obes Surg* 2016; **26**(8): 1794-8.
25. Manning S, Pucci A, Carter NC, et al. Early postoperative weight loss predicts maximal weight loss after sleeve gastrectomy and Roux-en-Y gastric bypass. *Surg Endosc* 2015; **29**(6): 1484-91.
26. Odom J, Zalesin KC, Washington TL, et al. Behavioral predictors of weight regain after bariatric surgery. *Obes Surg* 2010; **20**(3): 349-56.
27. Inge TH, Courcoulas AP, Jenkins TM, et al. Five-Year Outcomes of Gastric Bypass in Adolescents as Compared with Adults. *N Engl J Med* 2019; **380**(22): 2136-45.
28. Zhou K, Wolski K, Malin SK, et al. Impact of Weight Loss Trajectory Following Randomization to Bariatric Surgery on Long-Term Diabetes Glycemic and Cardiometabolic Parameters. *Endocr Pract* 2019; **25**(6): 572-9.
29. Nicoletti CF, de Oliveira BA, de Pinhel MA, et al. Influence of excess weight loss and weight regain on biochemical indicators during a 4-year follow-up after Roux-en-Y gastric bypass. *Obes Surg* 2015; **25**(2): 279-84.
30. Brethauer SA, Aminian A, Romero-Talamas H, et al. Can diabetes be surgically cured? Long-term metabolic effects of bariatric surgery in obese patients with type 2 diabetes mellitus. *Annals of surgery* 2013; **258**(4): 628-36; discussion 36-7.
31. Cohen RV, Rubino F, Schiavon C, Cummings DE. Diabetes remission without weight loss after duodenal bypass surgery. *Surg Obes Relat Dis* 2012; **8**(5): e66-8.
32. Cohen RV, Pinheiro JC, Schiavon CA, Salles JE, Wajchenberg BL, Cummings DE. Effects of gastric bypass surgery in patients with type 2 diabetes and only mild obesity. *Diabetes Care* 2012; **35**(7): 1420-8.
33. Kalarchian MA, Marcus MD. Psychosocial Interventions Pre and Post Bariatric Surgery. *Eur Eat Disord Rev* 2015; **23**(6): 457-62.
34. Nor Hanipah Z, Nasr EC, Bucak E, et al. Efficacy of adjuvant weight loss medication after bariatric surgery. *Surg Obes Relat Dis* 2018; **14**(1): 93-8.
35. Andreu A, Jimenez A, Vidal J, et al. Bariatric Support Groups Predicts Long-term Weight Loss. *Obes Surg* 2020.
36. Livhits M, Mercado C, Yermilov I, et al. Preoperative predictors of weight loss following bariatric surgery: systematic review. *Obes Surg* 2012; **22**(1): 70-89.


For peer review only - http://bmjopen.bmj.com/site/about/guidelines.xhtml

37. Mechanick JI, Youdim A, Jones DB, et al. Clinical practice guidelines for the perioperative nutritional, metabolic, and nonsurgical support of the bariatric surgery patient--2013 update: cosponsored by American Association of Clinical Endocrinologists, The Obesity Society, and American Society for Metabolic & Bariatric Surgery. *Obesity (Silver Spring)* 2013; **21 Suppl 1**: S1-27.
38. Parretti HM, Hughes CA, Jones LL. 'The rollercoaster of follow-up care' after bariatric surgery: a rapid review and qualitative synthesis. *Obes Rev* 2019; **20**(1): 88-107.

For peer review only



For peer review only - http://bmjopen.bmj.com/site/about/guidelines.xhtml

**Table 1. Baseline characteristics of the study population**

| | n | mean (sd) |
|---|---|---|
| Age at surgery | 5936 | 39·4 (9·0) |
| Sex, no. % female | 5936 | 79·1% |
| Height, cm | 5936 | 168·8 (8·9) |
| Weight, kg | 5936 | 122·8 (20·0) |
| Body mass index at surgery, kg/m$^2$ | 5936 | 42·9 (5·1) |
| **Glucose Metabolism** | | |
| Glucose, mmol/l | 2861 | 5·9 (1·9) |
| HbA1c, mmol/mol | 4168 | 40·6 (11·4) |
| Pharmacological diabetes treatment, no. (%) | 5936 | 675 (11·4%) |
| Diabetes type 2[A], no. (%) | 5936 | 896 15·1% |
| **Lipids** | | |
| High-density lipoprotein, mmol/l | 4188 | 1·2 (0·4) |
| Low-density lipoprotein, mmol/l | 4110 | 3·1 (0·9) |
| Triglycerides, mmol/l | 4314 | 1·7 (1·4) |
| Pharmacological dyslipidemia treatment, no. (%) | 5936 | 414 (7·0%) |
| Dyslipidemia[B], no. (%) | 5936 | 3601 67·5% |
| **Physiology** | | |
| Systolic BP, mm/Hg | 2960 | 133 (16) |
| Diastolic BP, mm/Hg | 2960 | 83 (10) |
| Pharmacological hypertension treatment, no. (%) | 5936 | 1158 (19·5%) |
| Hypertension[C], no. (%) | 5936 | 1683 28·4% |

[A] Pharmacologically treated T2D | fasting glucose >7·0mmol/l | HbA1c >48mmol/mol
[B] Pharmacologically treated dyslipidemia | LDL >4·1 | TG > 2·0 | HDL <1mmol/L for males and <1·3mmol/L for females
[C] Pharmacologically treated blood pressure | Systolic- >140mm/Hg or Diastolic blood pressure >90mm/Hg

For peer review only

**Table 2. Change in cardiometabolic disease status from baseline to five years post-surgery compared between surgical treatment failure (STF) and non-STF.**

| | Type 2 diabetes[A] | | Dyslipidemia[B] | | Hypertension[C] | |
|---|---|---|---|---|---|---|
| | STF n = 1135 | Non-STF n = 3878 | STF n = 1120 | Non-STF n = 3867 | STF n = 1126 | Non-STF n = 3842 |
| **No disease at baseline** | **n = 882** | **n = 3379** | **n = 377** | **n = 1616** | **n = 735** | **n = 2818** |
| *Disease-free* | *97·4%* | *98·5%* | *82·0%* | *87·3%* | *83·9%**** | *91·3%* |
| *Incidence* | *1·6%*** | *0·7%* | *9·5%**** | *4·9%* | *9·9%**** | *4·6%* |
| *Intermittent* | *1·0%* | *0·9%* | *8·5%* | *7·8%* | *6·1%** | *4·0%* |
| **Disease at baseline** | **n = 253** | **n = 499** | **n = 743** | **n = 2251** | **n =391** | **n = 1024** |
| *Remission* | *51·4%**** | *66·5%* | *63·7%**** | *81·1%* | *38·6%**** | *54·6%* |
| *No remission* | *26·1%* | *22·4%* | *17·2%**** | *8·8%* | *37·6%**** | *27·1%* |
| *Relapse* | *22·5%**** | *11·0%* | *19·1%**** | *10·1%* | *23·8%** | *18·3%* |

*indicates a statistically significant difference at p<·05
**indicates a statistically significant difference at p<·010
***indicates a statistically significant difference at p<·001

[A] pharmacologically treated T2D | fasting glucose >7·0mmol/l | HbA1c >48mmol/mol

[B] Pharmacologically treated dyslipidemia | LDL >4·1 | TG > 2·0 | HDL <1mmol/L for males and <1·3mmol/L for females
[C] Pharmacologically treated blood pressure | Systolic- >140mm/Hg or Diastolic blood pressure >90mm/Hg


For peer review only - http://bmjopen.bmj.com/site/about/guidelines.xhtml
For peer review only

**Table 3. Final multivariable model for predicting surgical treatment failure five years after surgery**

| | Beta (B) | S.E. | Wald | p | Exp(B) | 95%Confidence interval |
|---|---|---|---|---|---|---|
| Sex (0=male) | -0·00545 | 0·099 | 0·003 | 0·956 | 0·995 | 0·818-1·209 |
| Age at surgery, years | 0·00299 | 0·005 | 0·361 | 0·548 | 1·003 | 0·993-1·013 |
| BMI at surgery, kg/m$^2$ | 0·14949 | 0·009 | 283·640 | 0·000 | 1·161 | 1·141-1·182 |
| Percentage BMI loss during year one, %TWL | 0·22310 | 0·008 | 794·848 | 0·000 | 1·250 | 1·231-1·269 |
| Change in weight between year one and two, kg | 0·15982 | 0·008 | 382·606 | 0·000 | 1·173 | 1·155-1·192 |
| Intercept | -1·09588 | 0·513 | 4·569 | 0·033 | 0·334 | |

BMI – Body mass index
S.E. – Standard error

For peer review only

**Figure 1. Flowchart of the study participants.**

**Figure 2. Venn diagram of the prevalence of developing surgical treatment failure five years post-surgery according to three definitions: %excess BMI loss (n=1138), BMI >35 or >40 (n=835) and <20% total weight loss (n=1010).**

**Figure 3. Receiver operating characteristic curve with predicted probability of surgical treatment failure, given age, sex and baseline BMI and %TWL from baseline to the one year follow-up and change in weight (kg) between year one and year two follow-ups: area under the curve = 0·8743 (95% confidence interval 0·8630-0·8856).**

For peer review only


For peer review only - http://bmjopen.bmj.com/site/about/guidelines.xhtml

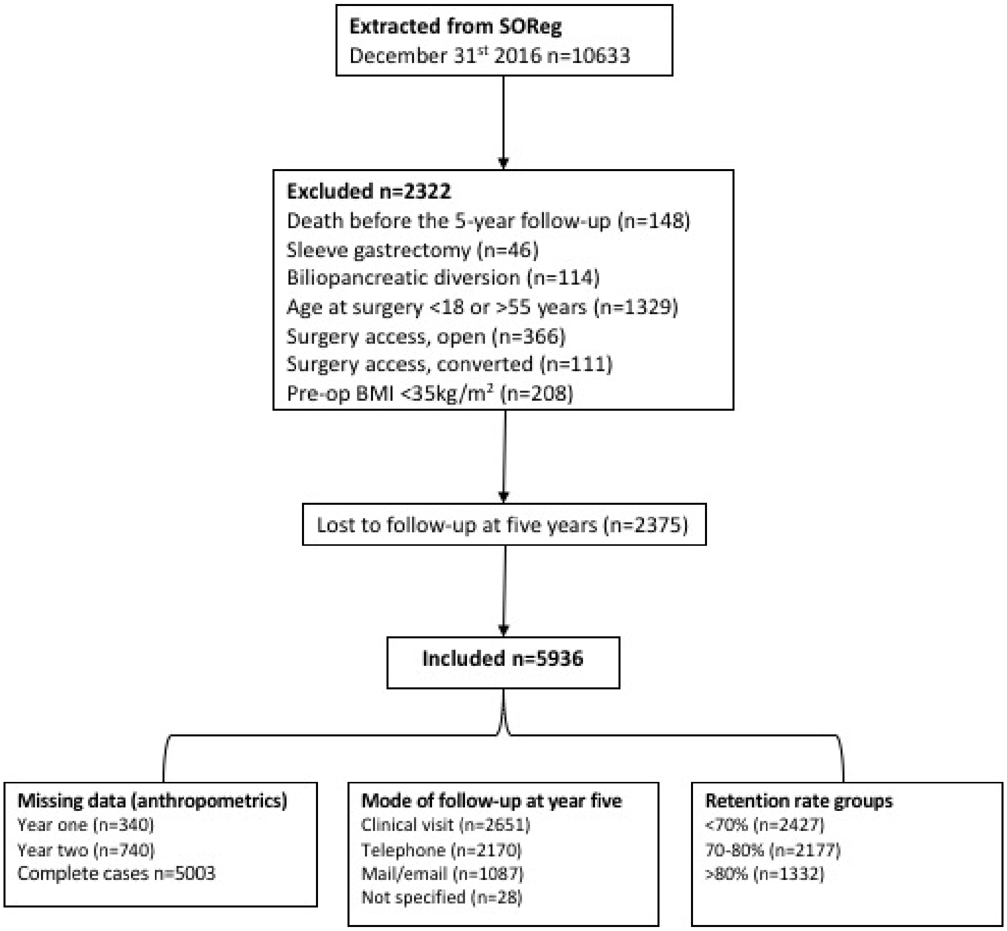


179x165mm (72 x 72 DPI)

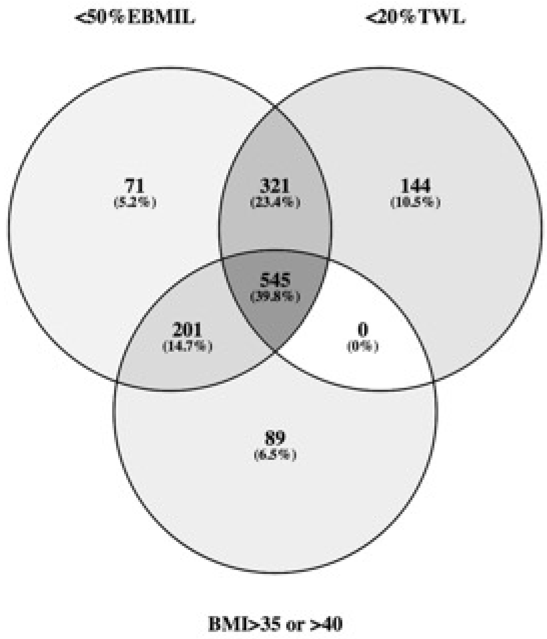


123x123mm (72 x 72 DPI)

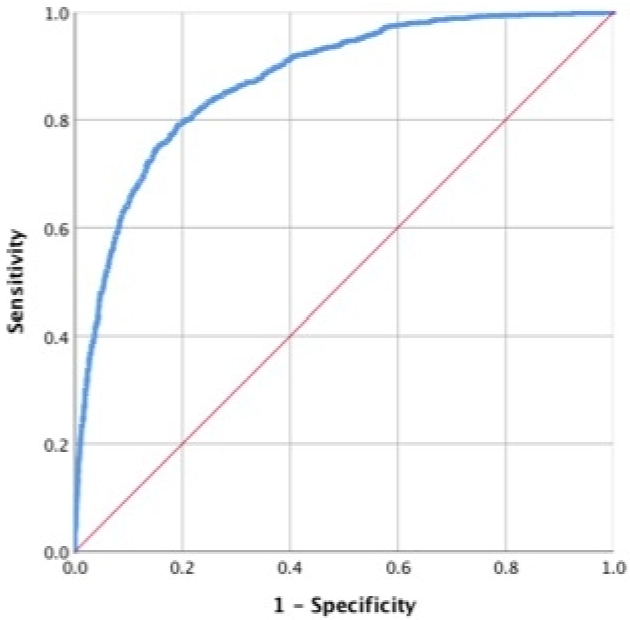


121x111mm (72 x 72 DPI)

Online-only Supplements

**eAppendix 1.** Cut-offs used to define cardiometabolic disease

**eAppendix 2.** Sensitivity analysis

**eAppendix 3.** Examples of risk calculation

**eFigure 1 a-g.** Unadjusted mean participants with pharmacological treatment of dyslipidemia and/or type 2 diabetes or hypertension at baseline, stratified on surgical treatment failure

**eFigure 2 a-g.** Unadjusted mean participants without pharmacological treatment of dyslipidemia and/or type 2 diabetes or hypertension at baseline, stratified on surgical treatment failure

**eFigure 3.a-c.** Predicted probability of type 2 diabetes at year five after surgery plotted over a, percentage weight loss from baseline to year five, b, excess body mass index loss from baseline to year five, c, body mass index at year five

**eFigure 4.a-c.** Predicted probability of dyslipidemia at year five after surgery plotted over a, percentage weight loss from baseline to year five, b, excess body mass index loss from baseline to year five, c, body mass index at year five.

**eFigure 5.a-c.** Predicted probability of Hypertension at year five after surgery plotted over a, percentage weight loss from baseline to year five, b, excess body mass index loss from baseline to year five, c, body mass index at year five.

**eTable 1.** Comparison of baseline characteristics between included participants and those lost to follow-up at year five

**eTable 2.** Percentage of available data on pharmacological treatment, biochemistry and blood pressure.

**eTable 3.** Comparison of baseline characteristics between surgical treatment failure and non-surgical treatment failure

**eTable 4.** Odds of cardiometabolic disease at year five, separate models for each definitions of surgical treatment failure

**eTable 5.** Odds of Cardiometabolic disease at year five for subjects reaching one, two or three of the three definitions

For peer review only

## eAppendix 1. Cut-offs used to define cardiometabolic disease

LDL>3.0mmol/L
HDL <1.0mmol/L for males and <1.3mmol/L for females
Triglycerides >2.0mmol/L
Fasting glucose >7.0mmol/L
HbA1c >48mmol/mol
Systolic- >140mmHg or Diastolic blood pressure >90mmHg

Conversion to mg/dL is done by a multiplying factor of 38.67 for LDL and HDL, 88.57 for TG, 18 for fasting glucose and using the formula (0.09148*IFCC) + 2.152 for HbA1c(%).

For peer review only


For peer review only - http://bmjopen.bmj.com/site/about/guidelines.xhtml

## eAppendix 2. Sensitivity analysis

We compared participants lost to follow-up with those not lost and found that the former had a higher prevalence of surgical treatment failure at year one (12.3% vs. 10.3%, p=0.015) and a similar prevalence (13.3% vs. 11.6%) at year two (p=0.081). Within the sample, there was a strong carryover effect, as 73.5% who met the definition of surgical treatment failure in year one and 79.5% in year two also met the definition at year five.

Additionally, participants who visited the clinic for follow-up had a higher prevalence of surgical treatment failure (26.7%) compared with other modes of follow-up (20.2%, p<0.001).

No statistical differences in prevalence of surgical treatment failure at year five were evident between retention groups 60-70%, 70-80% and >80%, (23.8% vs. 23.4% vs. 21.4%) (p=0.235). We found no statistical difference according to the year of surgery (p=0.280), or surgical volume (<50 [23.7%] vs. ≥50 [23.0%] LRYGB per year, p=0.695).

There was a crude difference in the prevalence of surgical treatment failure between males and females (males 31.6% vs. females 20.9%, p<0.001): OR=1.46 (95%CI 1.26-1.69) after baseline adjustments for age, BMI, T2D, hypertension and dyslipidemia. Males experienced a lower %TWL from baseline to all follow-up periods (data not shown).

At baseline, T2D (25.1% vs. 12.4%) and hypertension (44.4% vs. 24.1%) were more common among males (both p<0.001), whereas dyslipidemia was more common among females (61.7% vs. 56.9%, p=0.002).

We also found an association between those who had cardiometabolic disease present at baseline and surgical treatment failure at year five. The presence of T2D at baseline was associated with surgical treatment failure (OR=1.70; 95%CI 1.44-2.00), as was dyslipidemia at baseline (OR=1.30; 95%CI 1.15-1.48) and hypertension at baseline (OR=1.16; 95%CI 1.01-1.34), all adjusted for sex, age and BMI.

In terms of the magnitude of prediction factors, four additional models were assessed, one omitting change in weight between year one and two (AUC = 0.8260); another omitting %TWL at year one (AUC = 0.7214). Third, we assessed a model adding cardiometabolic disease (T2D, dyslipidemia, hypertension) at baseline as additional predictors. With this latter model, we found that only dyslipidemia remained significant and improved the model minimally (AUC = 0.8749). We also assessed a model restricted to variables available at baseline that included sex, age, BMI and cardiometabolic disease (AUC = 0.638).

For peer review only


For peer review only - http://bmjopen.bmj.com/site/about/guidelines.xhtml

**eAppendix 3. Examples of calculation of predicted probability meeting one of the definitions of surgical treatment failure at year five.**

Example 1: male, age 43 years, BMI = 45, -23%TWL during year one, +4 kg between year one and two

a = -1.1 + 0.00545*0 + 0.00299*43 + 0.14949*45 + 0.22310*-23 + 0.15982*4
exp(a)/(1+(a)) = 0.78

Hence, predicted probability of surgical treatment failure at year five is 78%.

Example 2: male, age 43 years, BMI = 49, -33%TWL during year one, -1 kg between year one and two,
a = -1.1 + 0.00545*0 + 0.00299*43 + 0.14949*49 + 0.22310*-33 + 0.15982*-1
This yields a predicted probability of 23.8% for surgical treatment failure at year five.

For peer review only


For peer review only - http://bmjopen.bmj.com/site/about/guidelines.xhtml

**eFigure 1 a-g. Unadjusted mean values (bars) with error bars (95% confidence intervals) for participants <u>with</u> pharmacological treatment of dyslipidemia and/or type 2 diabetes or hypertension at baseline, stratified on surgical treatment failure. a) low density lipoprotein, b) high density lipoprotein, c) triglycerides, d) fasting glucose, e) HbA1c, f) systolic blood pressure, g) diastolic blood pressure. Tables below figures show number available at each timepoint and prevalence above cut-off. Bold font with * indicates a difference p<0.05).**

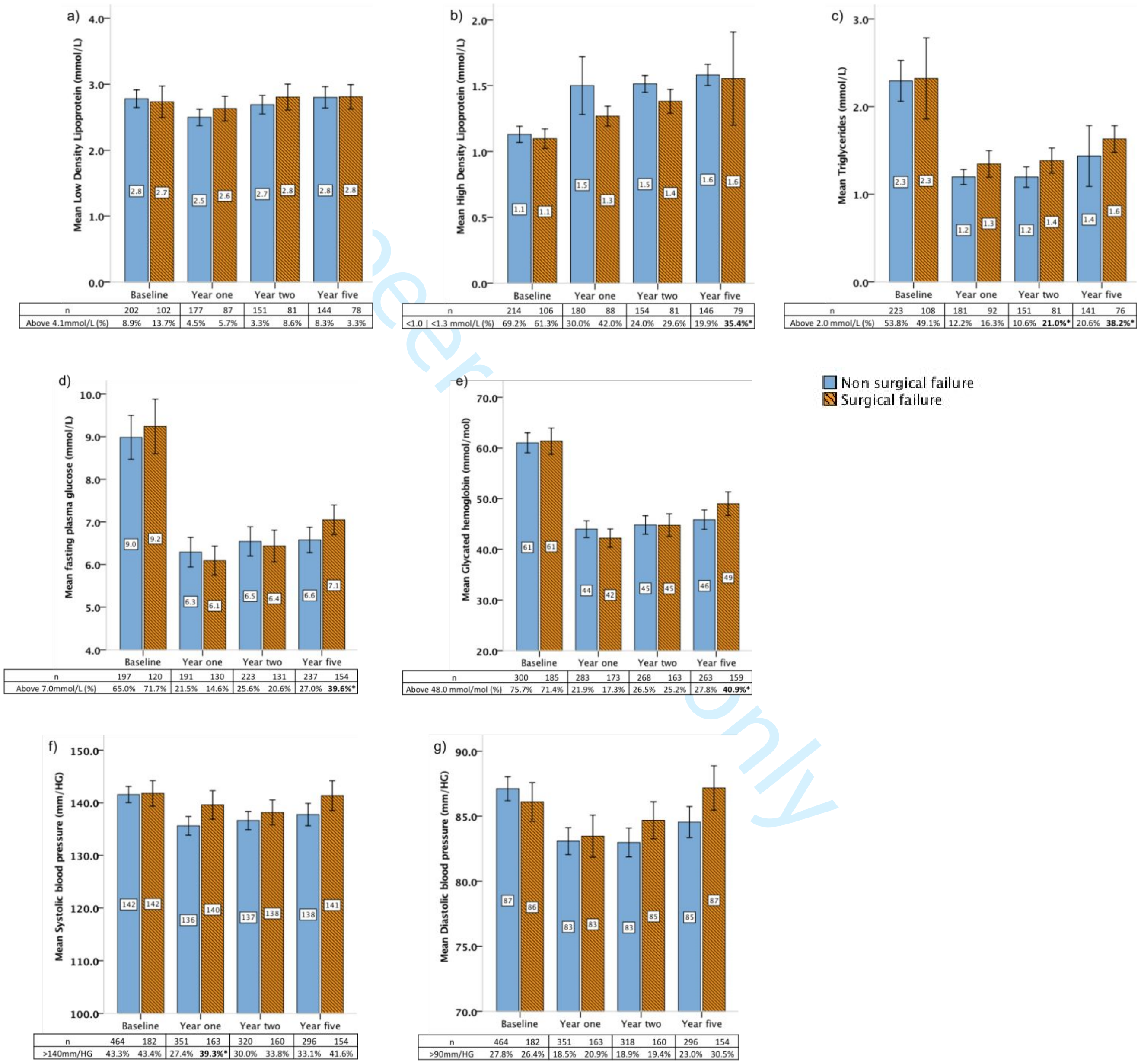


a)

| | Baseline | | Year one | | Year two | | Year five | |
|---|---|---|---|---|---|---|---|---|
| n | 202 | 102 | 177 | 87 | 151 | 81 | 144 | 78 |
| Above 4.1mmol/L (%) | 8.9% | 13.7% | 4.5% | 5.7% | 3.3% | 8.6% | 8.3% | 3.3% |

b)

| | Baseline | | Year one | | Year two | | Year five | |
|---|---|---|---|---|---|---|---|---|
| n | 214 | 106 | 180 | 88 | 154 | 81 | 146 | 79 |
| <1.0 \| <1.3 mmol/L (%) | 69.2% | 61.3% | 30.0% | 42.0% | 24.0% | 29.6% | 19.9% | **35.4%*** |

c)

| | Baseline | | Year one | | Year two | | Year five | |
|---|---|---|---|---|---|---|---|---|
| n | 223 | 108 | 181 | 92 | 151 | 81 | 141 | 76 |
| Above 2.0 mmol/L (%) | 53.8% | 49.1% | 12.2% | 16.3% | 10.6% | **21.0%*** | 20.6% | **38.2%*** |

d)

| | Baseline | | Year one | | Year two | | Year five | |
|---|---|---|---|---|---|---|---|---|
| n | 197 | 120 | 191 | 130 | 223 | 131 | 237 | 154 |
| Above 7.0mmol/L (%) | 65.0% | 71.7% | 21.5% | 14.6% | 25.6% | 20.6% | 27.0% | **39.6%*** |

e)

| | Baseline | | Year one | | Year two | | Year five | |
|---|---|---|---|---|---|---|---|---|
| n | 300 | 185 | 283 | 173 | 268 | 163 | 263 | 159 |
| Above 48.0 mmol/mol (%) | 75.7% | 71.4% | 21.9% | 17.3% | 26.5% | 25.2% | 27.8% | **40.9%*** |

f)

| | Baseline | | Year one | | Year two | | Year five | |
|---|---|---|---|---|---|---|---|---|
| n | 464 | 182 | 351 | 163 | 320 | 160 | 296 | 154 |
| >140mm/HG | 43.3% | 43.4% | 27.4% | **39.3%*** | 30.0% | 33.8% | 33.1% | 41.6% |

g)

| | Baseline | | Year one | | Year two | | Year five | |
|---|---|---|---|---|---|---|---|---|
| n | 464 | 182 | 351 | 163 | 318 | 160 | 296 | 154 |
| >90mm/HG | 27.8% | 26.4% | 18.5% | 20.9% | 18.9% | 19.4% | 23.0% | 30.5% |

**eFigure 2 a-g. Unadjusted mean values (bars) with error bars (95% confidence intervals) for participants <u>without</u> pharmacological treatment of dyslipidemia and/or type 2 diabetes or hypertension at baseline, stratified on surgical treatment failure. a) low density lipoprotein, b) high density lipoprotein, c) triglycerides, d) fasting glucose, e) HbA1c, f) systolic blood pressure, g) diastolic blood pressure. Tables below figures show number available at each timepoint and prevalence above cut-off. Bold font with * indicates a difference (p<0.05).**

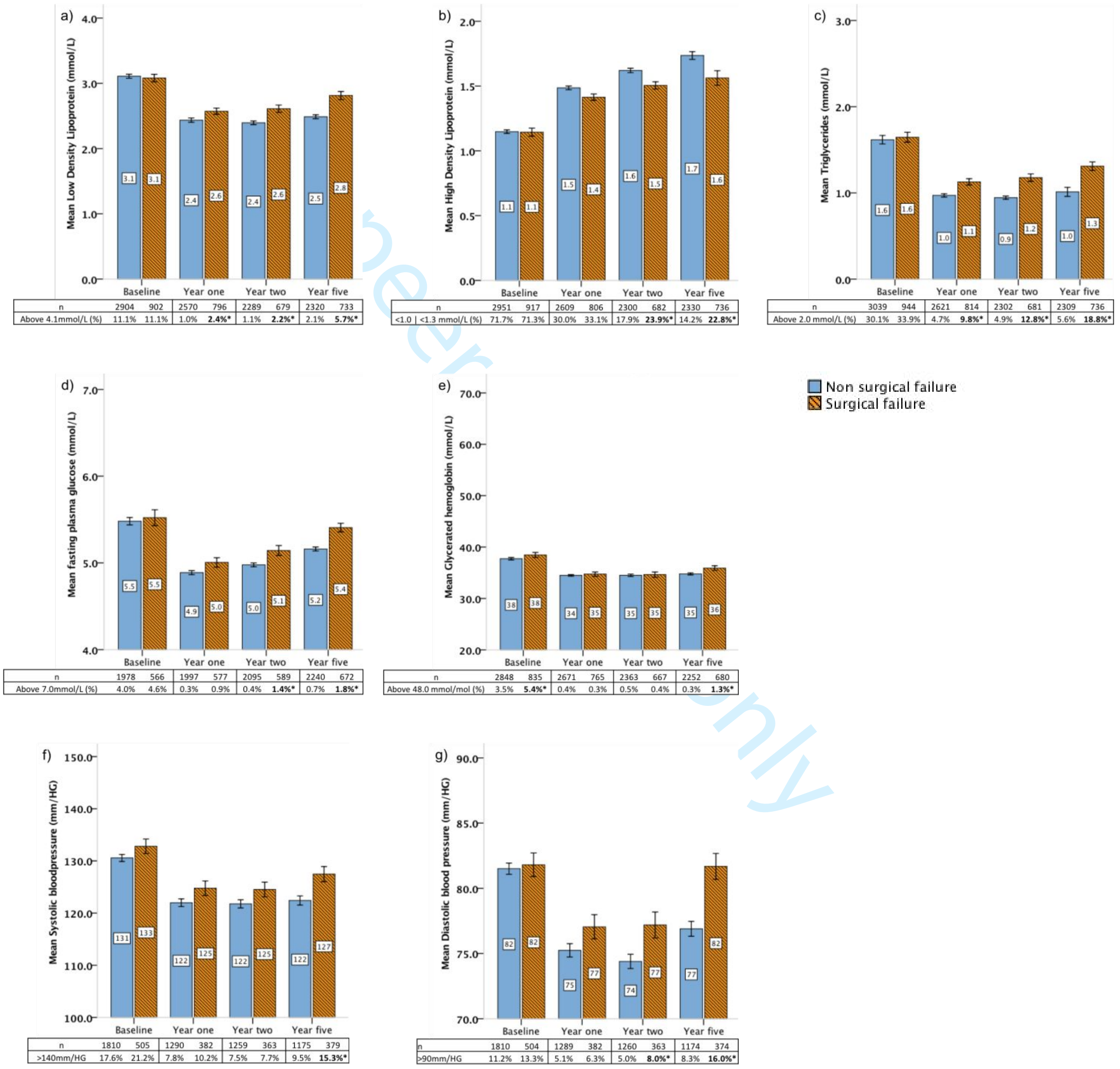


a)

| | Baseline | | Year one | | Year two | | Year five | |
|---|---|---|---|---|---|---|---|---|
| n | 2904 | 902 | 2570 | 796 | 2289 | 679 | 2320 | 733 |
| Above 4.1mmol/L (%) | 11.1% | 11.1% | 1.0% | **2.4%*** | 1.1% | **2.2%*** | 2.1% | **5.7%*** |

b)

| | Baseline | | Year one | | Year two | | Year five | |
|---|---|---|---|---|---|---|---|---|
| n | 2951 | 917 | 2609 | 806 | 2300 | 682 | 2330 | 736 |
| <1.0 \| <1.3 mmol/L (%) | 71.7% | 71.3% | 30.0% | 33.1% | 17.9% | **23.9%*** | 14.2% | **22.8%*** |

c)

| | Baseline | | Year one | | Year two | | Year five | |
|---|---|---|---|---|---|---|---|---|
| n | 3039 | 944 | 2621 | 814 | 2302 | 681 | 2309 | 736 |
| Above 2.0 mmol/L (%) | 30.1% | 33.9% | 4.7% | **9.8%*** | 4.9% | **12.8%*** | 5.6% | **18.8%*** |

d)

| | Baseline | | Year one | | Year two | | Year five | |
|---|---|---|---|---|---|---|---|---|
| n | 1978 | 566 | 1997 | 577 | 2095 | 589 | 2240 | 672 |
| Above 7.0mmol/L (%) | 4.0% | 4.6% | 0.3% | 0.9% | 0.4% | **1.4%*** | 0.7% | **1.8%*** |

e)

| | Baseline | | Year one | | Year two | | Year five | |
|---|---|---|---|---|---|---|---|---|
| n | 2848 | 835 | 2671 | 765 | 2363 | 667 | 2252 | 680 |
| Above 48.0 mmol/mol (%) | 3.5% | **5.4%*** | 0.4% | 0.3% | 0.5% | 0.4% | 0.3% | **1.3%*** |

f)

| | Baseline | | Year one | | Year two | | Year five | |
|---|---|---|---|---|---|---|---|---|
| n | 1810 | 505 | 1290 | 382 | 1259 | 363 | 1175 | 379 |
| >140mm/HG | 17.6% | 21.2% | 7.8% | 10.2% | 7.5% | 7.7% | 9.5% | **15.3%*** |

g)

| | Baseline | | Year one | | Year two | | Year five | |
|---|---|---|---|---|---|---|---|---|
| n | 1810 | 504 | 1289 | 382 | 1260 | 363 | 1174 | 374 |
| >90mm/HG | 11.2% | 13.3% | 5.1% | 6.3% | 5.0% | **8.0%*** | 8.3% | **16.0%*** |


For peer review only - http://bmjopen.bmj.com/site/about/guidelines.xhtml

**eFigure 3.a-c. Predicted probability of type 2 diabetes at year five after surgery plotted over a, percentage weight loss from baseline to year five, b, excess body mass index loss from baseline to year five, c, body mass index at year five. Groups based on presence on presence of type 2 diabetes at baseline. Adjusted for age, sex, body mass index and type 2 diabetes at baseline.**

a,

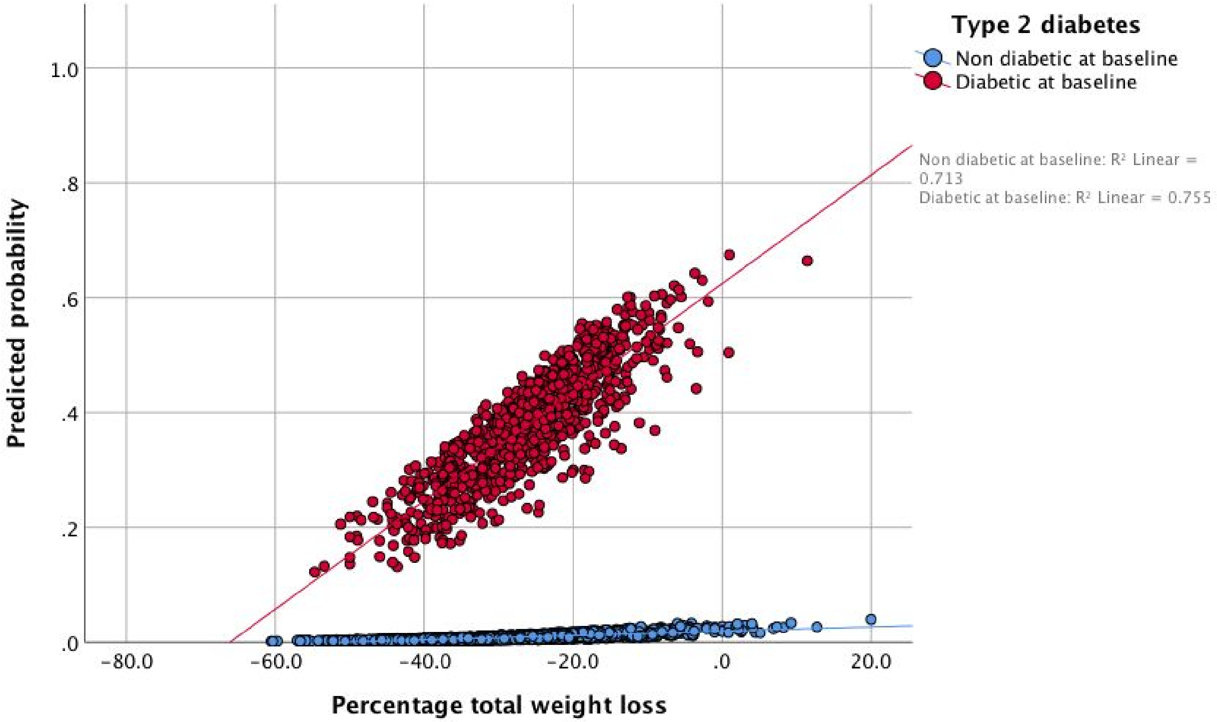



For peer review only - http://bmjopen.bmj.com/site/about/guidelines.xhtml

b,

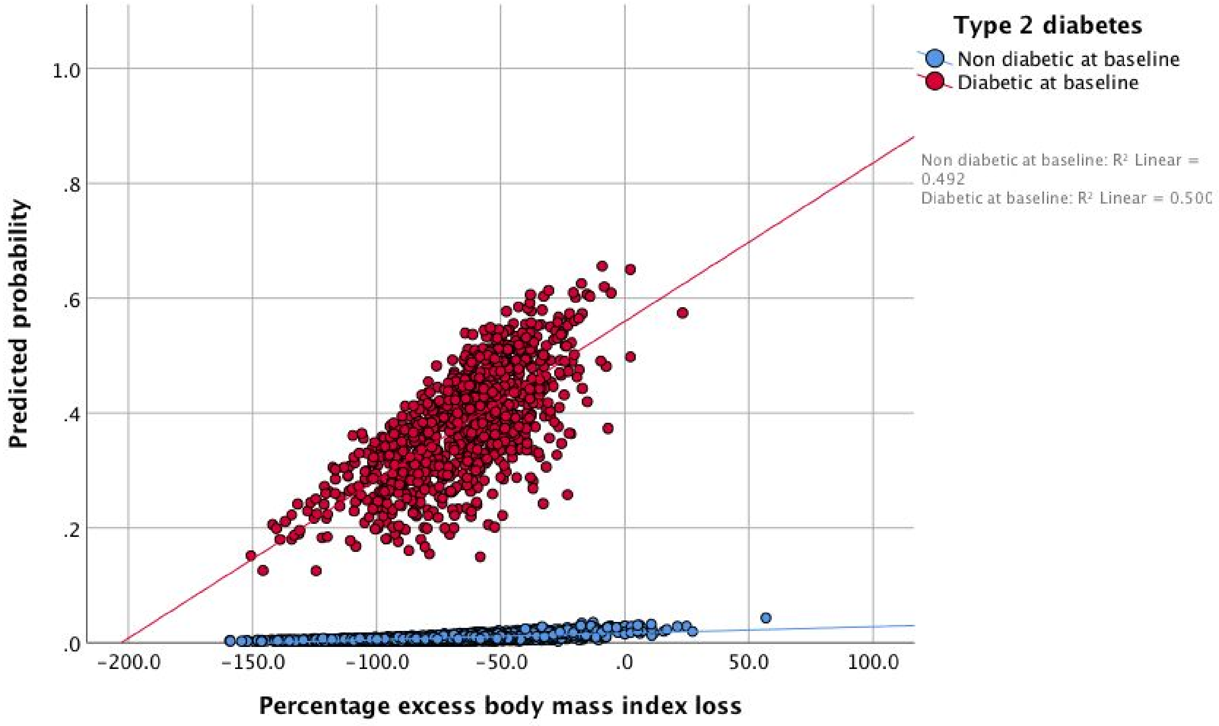


c,

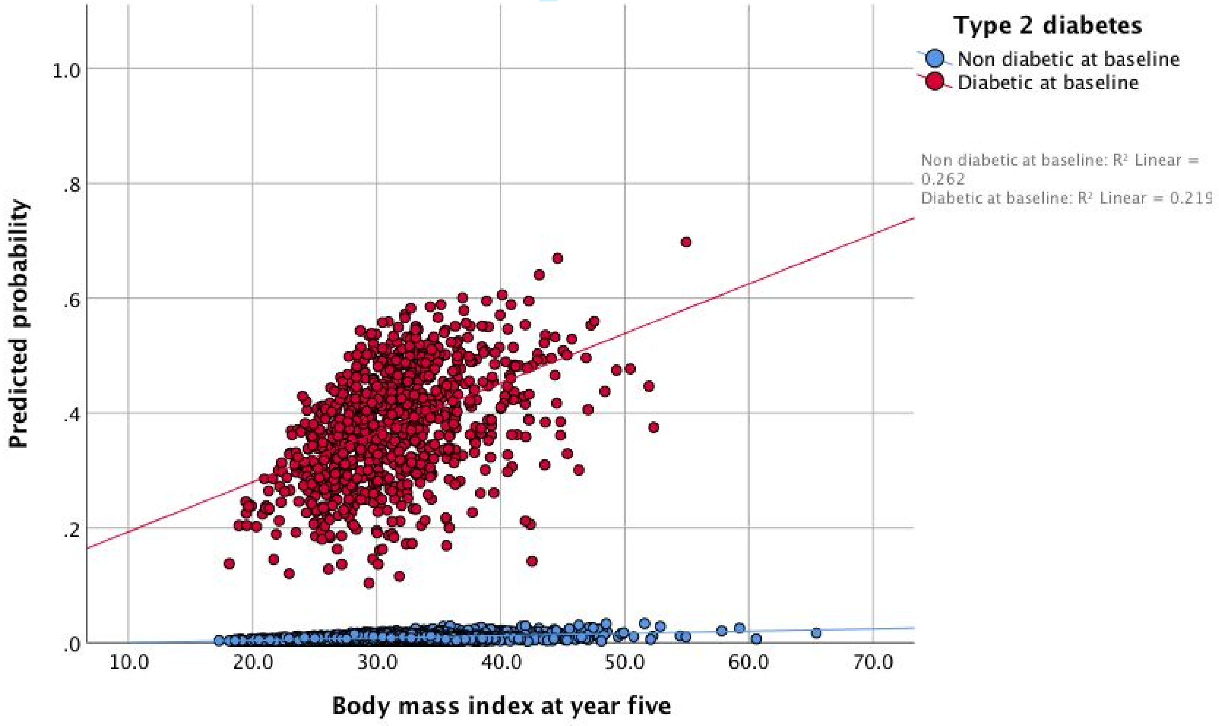




For peer review only - http://bmjopen.bmj.com/site/about/guidelines.xhtml

**eFigure 4.a-c .Predicted probability of dyslipidemia at year five after surgery plotted over a, percentage weight loss from baseline to year five, b, excess body mass index loss from baseline to year five, c, body mass index at year five. Groups based on presence of dyslipidemia at baseline. Adjusted for age, sex, baseline body mass index and dyslipidemia at baseline.**

a,

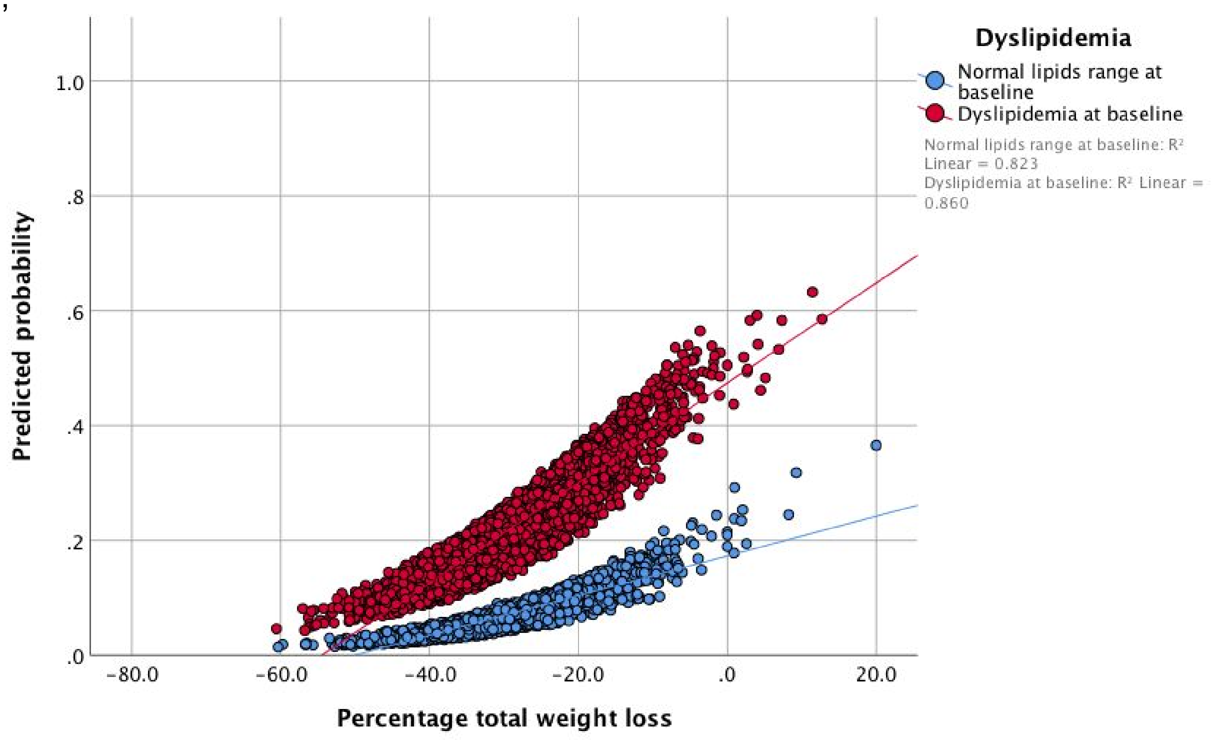



For peer review only - http://bmjopen.bmj.com/site/about/guidelines.xhtml

b,

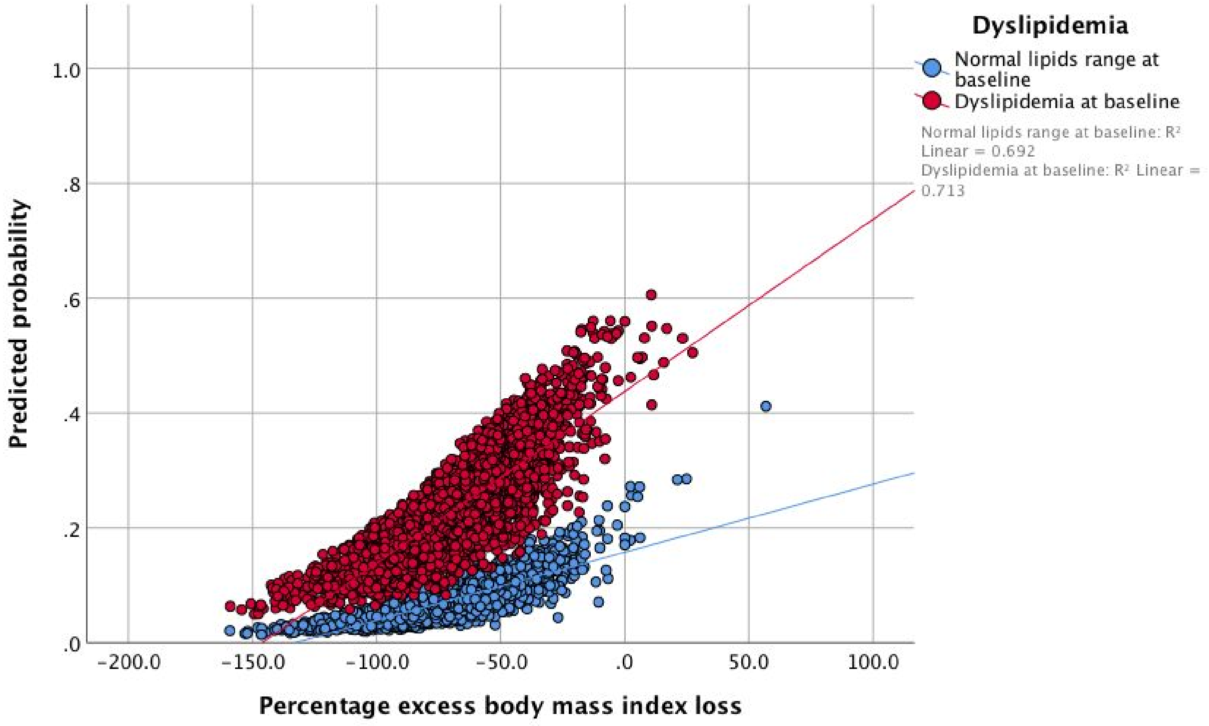


c,

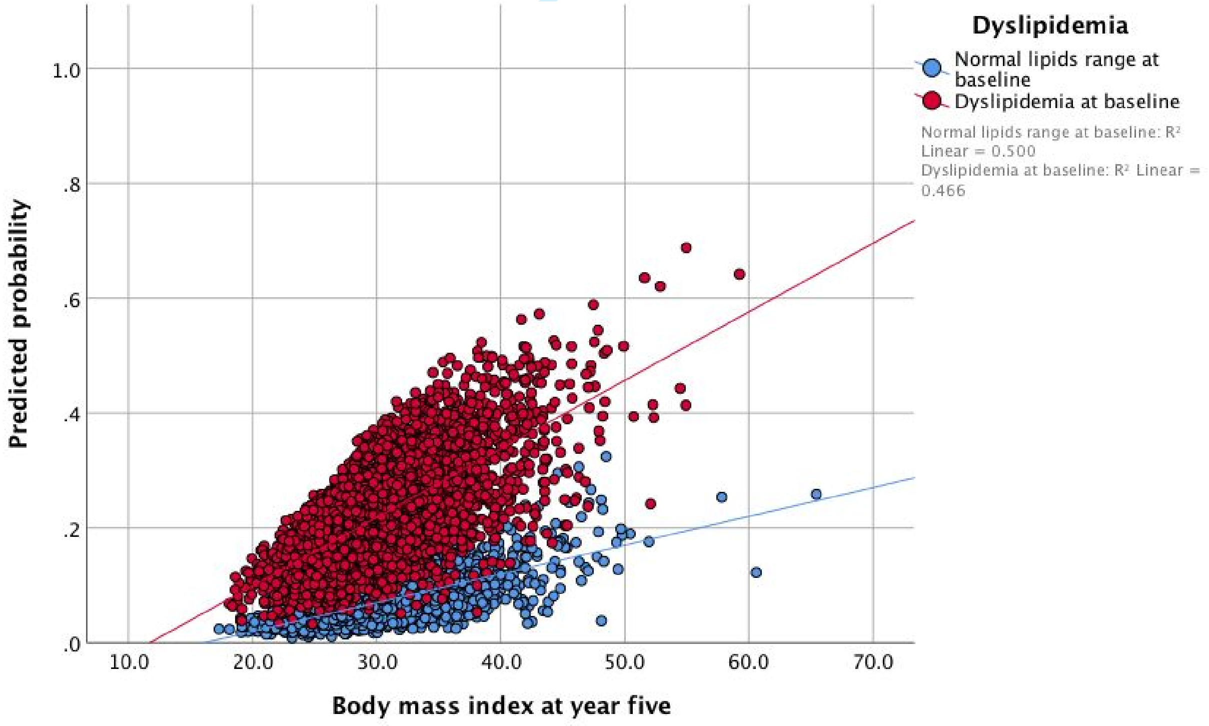




For peer review only - http://bmjopen.bmj.com/site/about/guidelines.xhtml

**eFigure 5.a-c. Predicted probability of Hypertension at year five after surgery plotted over a, percentage weight loss from baseline to year five, b, excess body mass index loss from baseline to year five, c, body mass index at year five. Groups based on presence of hypertension at baseline. Adjusted for age, sex, body mass index and hypertension at baseline.**

a,

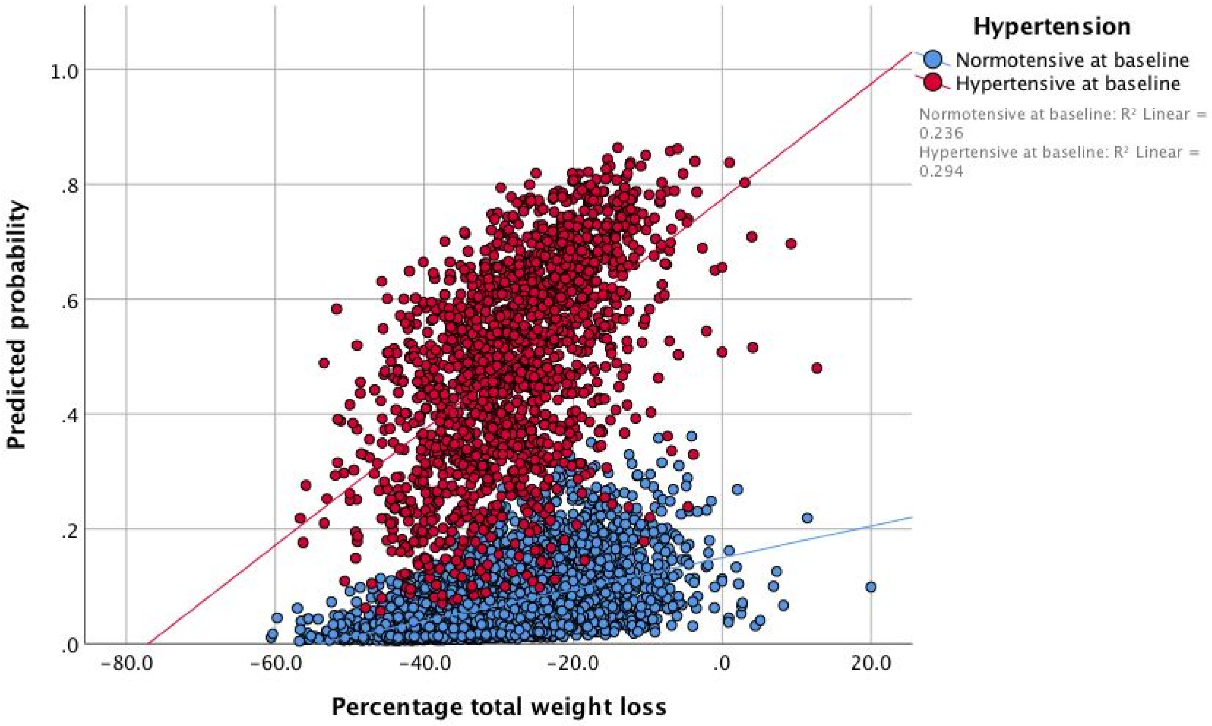


For peer review only

b,

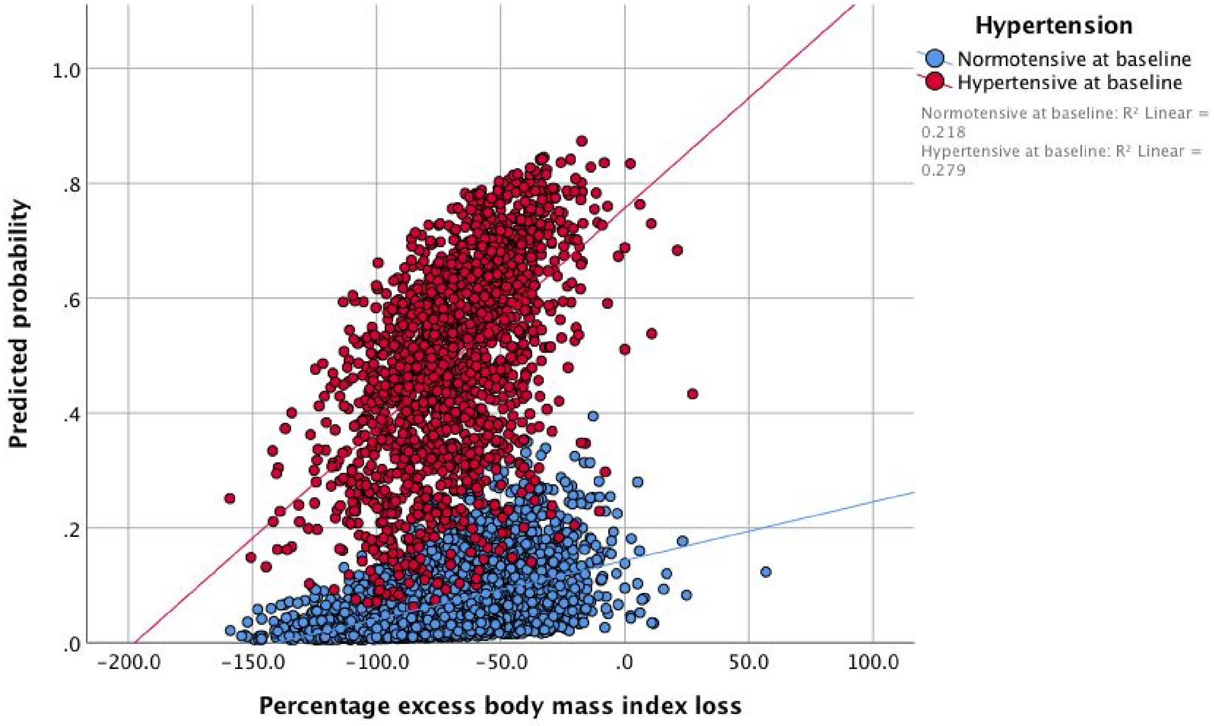


c,

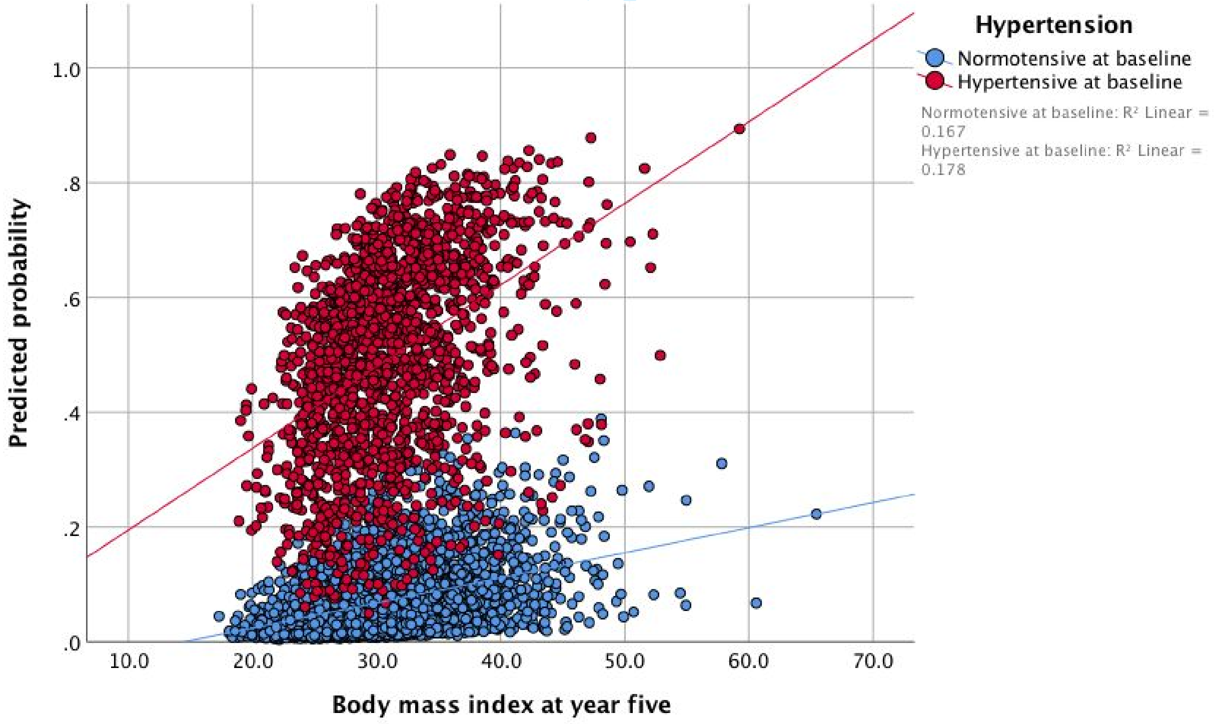



For peer review only - http://bmjopen.bmj.com/site/about/guidelines.xhtml

## eTable 1. Comparison of baseline characteristics between included participants and those lost to follow-up at year five

| | Included | | Lost to follow-up | | |
|---|---|---|---|---|---|
| | n | mean (sd) | n | mean (sd) | P |
| Age at surgery | 5936 | 39.4 (9.0) | 2375 | 37.6 (9.1) | <0.0001 |
| Sex, % female | 5936 | 79.10% | 2375 | 73.6% | <0.0001 |
| Height, cm | 5936 | 168.8 (8.9) | 2375 | 169.8 (9.3) | <0.0001 |
| Weight, kg | 5936 | 122.8 (20.0) | 2375 | 126.3 (20.7) | <0.0001 |
| BMI at surgery, kg/m$^2$ | 5936 | 42.9 (5.1) | 2375 | 43.6 (5.2) | <0.0001 |
| Glucose, mmol/l | 2861 | 5.9 (1.9) | 1312 | 5.8 (1.8) | 0.223 |
| HbA1c, mmol/mol | 4168 | 40.6 (11.4) | 1846 | 40.8 (11.7) | 0.528 |
| Pharmacological diabetes treatment | 5936 | 11.4% | 2375 | 9.6% | 0.022 |
| Diabetes type 2[A] | 5936 | 15.1% | 2375 | 14.6% | 0.543 |
| High-density lipoprotein, mmol/l | 4188 | 1.2 (0.4) | 1845 | 1.1 (0.5) | 0.017 |
| Low-density lipoprotein, mmol/l | 4110 | 3.1 (0.9) | 1798 | 3.1 (1.0) | 0.756 |
| Triglycerides, mmol/l | 4314 | 1.7 (1.4) | 1883 | 1.8 (2.2) | 0.087 |
| Pharmacological dyslipidemia treatment | 5936 | 7% | 2375 | 6.5% | 0.465 |
| Dyslipidemia[B] | 5936 | 67.5% | 2375 | 74.1% | <0.0001 |
| Systolic BP, mmHg | 2960 | 133 (16) | 1348 | 135 (17) | 0.015 |
| Diastolic BP, mmHg | 2960 | 83 (10) | 1347 | 83 (10) | 0.164 |
| Pharmacological hypertension treatment | 5936 | 19.5% | 2375 | 18.1% | 0.142 |
| Hypertension[C] | 5936 | 28.4% | 2375 | 29.3% | 0.365 |

BMI – Body mass index

[A] Pharmacologically treated T2D | fasting glucose >7.0mmol/l | HbA1c >48mmol/mol
[B] Pharmacologically treated dyslipidemia | LDL >4.1 | TG > 2.0 | HDL <1.0mmol/L for males and <1.3mmol/L for females
[C] Pharmacologically treated blood pressure | systolic- >140mm/Hg or diastolic blood pressure >90mm/Hg

For peer review only

**eTable 2. Percentage of the 5936 participants with available data on pharmacological treatment and on biochemistry and blood pressure at baseline, one, two and five years after surgery.**

| | Baseline | Year one | Year two | Year five |
|---|---|---|---|---|
| Pharmacological treatment | | | | |
| Type 2 diabetes | 100% | 94% | 88% | 100% |
| Dyslipidemia | 100% | 94% | 88% | 100% |
| Hypertension | 100% | 94% | 88% | 100% |
| | | | | |
| Biochemistry | | | | |
| Low-density lipoprotein | 69% | 61% | 54% | 55% |
| High-density lipoprotein | 71% | 62% | 54% | 55% |
| Triglycerides | 73% | 63% | 54% | 55% |
| Fasting glucose | 48% | 49% | 51% | 56% |
| HbA1c | 70% | 66% | 58% | 57% |
| Blood pressure | 50% | 37% | 35% | 34% |

For peer review only

## eTable 3. Comparison of baseline characteristics between surgical treatment failure (STF) and non-STF

| | STF | | Non-STF | | |
|---|---|---|---|---|---|
| | n | mean (sd) | n | mean (sd) | P |
| Age at surgery | 1371 | 40.5 (8.8) | 4565 | 39.1 (9.0) | <0.001 |
| Sex, % female | 1371 | 71.4% | 4565 | 81.4% | <0.001 |
| Height, cm | 1371 | 169.6 (9.4) | 4565 | 168.6 (8.7) | <0.001 |
| Weight, kg | 1371 | 128.2 (22.9) | 4565 | 121.2 (18.7) | <0.001 |
| BMI at surgery, $kg/m^2$ | 1371 | 44.4 (6.1) | 4565 | 42.5 (4.6) | <0.001 |
| **Glucose Metabolism** | | | | | |
| Glucose, mmol/l | 686 | 6.2 (2.3) | 2175 | 5.8 (1.8) | <0.001 |
| HbA1c, mmol/mol | 1020 | 42.6 (13.3) | 3148 | 40.0 (10.7) | <0.001 |
| Pharmacological Diabetes treatment | 1371 | 17.4% | 4565 | 9.6% | <0.001 |
| Diabetes type 2[A] | 1371 | 22.0% | 4565 | 13.0% | <0.001 |
| **Lipids** | | | | | |
| High-density lipoprotein, mmol/l | 1023 | 1.1 (0.5) | 3165 | 1.2 (0.4) | 0.605 |
| Low-density lipoprotein, mmol/l | 1004 | 3.1 (0.9) | 3106 | 3.1 (0.9) | 0.201 |
| Triglycerides, mmol/l | 1052 | 1.7 (1.2) | 3262 | 1.7 (1.4) | 0.285 |
| Pharmacological dyslipidemia treatment | 1371 | 9.6% | 4565 | 6.2% | <0.001 |
| Dyslipidemia[B] | 1371 | 72.3% | 4565 | 66.0% | <0.001 |
| **Physiology** | | | | | |
| Systolic BP, mmHg | 687 | 135 (16) | 2274 | 133 (16) | 0.001 |
| Diastolic BP, mmHg | 686 | 83 (11) | 2274 | 83 (10) | 0.509 |
| Pharmacological hypertension treatment | 1371 | 24.8% | 4565 | 17.9% | <0.001 |
| Hypertension[C] | 1371 | 34.2% | 4565 | 26.6% | <0.001 |

[A] Pharmacologically treated T2D | fasting glucose >7.0mmol/l | HbA1c >48mmol/mol
[B] Pharmacologically treated dyslipidemia | LDL >4.1 | TG > 2.0 | HDL <1.0mmol/L for males and <1.3mmol/L for females
[C] Pharmacologically treated blood pressure | systolic- >140mm/Hg or diastolic blood pressure >90mm/Hg

For peer review only



For peer review only - http://bmjopen.bmj.com/site/about/guidelines.xhtml

**eTable 4. Odds of cardiometabolic disease at year five, separate models for each definitions of surgical treatment failure. Adjusted for sex and baseline; age, BMI and corresponding cardiometabolic disease.**

| | Beta | Standard error | Odds ratio | 95% Confidence interval Lower | Upper | p |
|---|---|---|---|---|---|---|
| **Type 2 diabetes** | | | | | | |
| Non surgical treatment failure (ref) | | | | | | |
| Total weight loss <20% | 0.818 | 0.142 | 2.266 | 1.715 | 2.995 | <0.001 |
| Excess BMI loss <50% | 0.760 | 0.144 | 2.138 | 1.611 | 2.837 | <0.001 |
| BMI >35 or >40* | 0.893 | 0.184 | 2.441 | 1.703 | 3.499 | <0.001 |
| Compiled** | 0.743 | 0.137 | 2.102 | 1.608 | 2.749 | <0.001 |
| **Dyslipidemia** | | | | | | |
| Non surgical treatment failure (ref) | | | | | | |
| Total weight loss <20% | 0.946 | 0.084 | 2.574 | 2.185 | 3.033 | <0.001 |
| Excess BMI loss <50% | 0.935 | 0.083 | 2.548 | 2.164 | 3.000 | <0.001 |
| BMI >35 or >40* | 0.863 | 0.100 | 2.370 | 1.949 | 2.883 | <0.001 |
| Compiled** | 0.916 | 0.079 | 2.500 | 2.143 | 2.918 | <0.001 |
| **Hypertension** | | | | | | |
| Non surgical treatment failure (ref) | | | | | | |
| Total weight loss <20% | 0.687 | 0.098 | 1.988 | 1.642 | 2.407 | <0.001 |
| Excess BMI loss <50% | 0.652 | 0.095 | 1.920 | 1.593 | 2.315 | <0.001 |
| BMI >35 or >40* | 0.569 | 0.114 | 1.767 | 1.413 | 2.210 | <0.001 |
| Compiled** | 0.616 | 0.089 | 1.851 | 1.554 | 2.206 | <0.001 |

*For subjects with presurgery BMI of <50 and >50, respectively.
**Defined as meeting any of the definitions, <20%TWL | <50%EBMIL | BMI >35 or >40.

For peer review only

**eTable 5. Odds of Cardiometabolic disease at year five for subjects reaching one, two or three of the three definitions (exclusively, subjects may only be in one group). Adjusted for sex and baseline; age, BMI and corresponding cardiometabolic disease.**

| | Beta | Standard error | Odds ratio | 95% Confidence Interval Lower | Upper | p |
|---|---|---|---|---|---|---|
| **Type 2 diabetes** | | | | | | |
| Non surgical treatment failure (ref) | | | | | | |
| Surgical failure 1/3 | 0.409 | 0.258 | 1.506 | 0.908 | 2.496 | 0.113 |
| Surgical failure 2/3 | 0.592 | 0.186 | 1.808 | 1.255 | 2.606 | 0.001 |
| Surgical failure 3/3 | 1.119 | 0.198 | 3.061 | 2.078 | 4.509 | <0.001 |
| **Dyslipidemia** | | | | | | |
| Non surgical treatment failure (ref) | | | | | | |
| Surgical failure 1/3 | 0.605 | 0.147 | 1.832 | 1.373 | 2.445 | <0.001 |
| Surgical failure 2/3 | 0.961 | 0.111 | 2.614 | 2.103 | 3.250 | <0.001 |
| Surgical failure 3/3 | 1.050 | 0.111 | 2.857 | 2.299 | 3.550 | <0.001 |
| **Hypertension** | | | | | | |
| Non surgical treatment failure (ref) | | | | | | |
| Surgical failure 1/3 | 0.415 | 0.165 | 1.515 | 1.097 | 2.091 | 0.012 |
| Surgical failure 2/3 | 0.556 | 0.130 | 1.744 | 1.352 | 2.248 | <0.001 |
| Surgical failure 3/3 | 0.798 | 0.129 | 2.221 | 1.724 | 2.862 | <0.001 |

For peer review only

STROBE Statement—checklist of items that should be included in reports of observational studies

| | Item No | Recommendation | Page No |
|---|---|---|---|
| **Title and abstract** | 1 | (*a*) Indicate the study's design with a commonly used term in the title or the abstract | 1 |
| | | (*b*) Provide in the abstract an informative and balanced summary of what was done and what was found | 3-4 |
| **Introduction** | | | |
| Background/rationale | 2 | Explain the scientific background and rationale for the investigation being reported | 5-6 |
| Objectives | 3 | State specific objectives, including any prespecified hypotheses | 6 |
| **Methods** | | | |
| Study design | 4 | Present key elements of study design early in the paper | 6-7 |
| Setting | 5 | Describe the setting, locations, and relevant dates, including periods of recruitment, exposure, follow-up, and data collection | 6-7 |
| Participants | 6 | (*a*) *Cohort study*—Give the eligibility criteria, and the sources and methods of selection of participants. Describe methods of follow-up<br>*Case-control study*—Give the eligibility criteria, and the sources and methods of case ascertainment and control selection. Give the rationale for the choice of cases and controls<br>*Cross-sectional study*—Give the eligibility criteria, and the sources and methods of selection of participants | 7 |
| | | (*b*) *Cohort study*—For matched studies, give matching criteria and number of exposed and unexposed<br>*Case-control study*—For matched studies, give matching criteria and the number of controls per case | |
| Variables | 7 | Clearly define all outcomes, exposures, predictors, potential confounders, and effect modifiers. Give diagnostic criteria, if applicable | 8-10 |
| Data sources/ measurement | 8* | For each variable of interest, give sources of data and details of methods of assessment (measurement). Describe comparability of assessment methods if there is more than one group | *8-10* |
| Bias | 9 | Describe any efforts to address potential sources of bias | 7 |
| Study size | 10 | Explain how the study size was arrived at | 7 |
| Quantitative variables | 11 | Explain how quantitative variables were handled in the analyses. If applicable, describe which groupings were chosen and why | 10-11 |
| Statistical methods | 12 | (*a*) Describe all statistical methods, including those used to control for confounding | 10-11 |
| | | (*b*) Describe any methods used to examine subgroups and interactions | 10-11 |
| | | (*c*) Explain how missing data were addressed | 7,18 |
| | | (*d*) *Cohort study*—If applicable, explain how loss to follow-up was addressed<br>*Case-control study*—If applicable, explain how matching of cases and controls was addressed<br>*Cross-sectional study*—If applicable, describe analytical methods taking account of sampling strategy | 7 |
| | | (*e*) Describe any sensitivity analyses | 10-11 |


For peer review only - http://bmjopen.bmj.com/site/about/guidelines.xhtml

Continued on next page


For peer review only - http://bmjopen.bmj.com/site/about/guidelines.xhtml

| | | | |
|---|---|---|---|
| **Results** | | | |
| Participants | 13* | (a) Report numbers of individuals at each stage of study—eg numbers potentially eligible, examined for eligibility, confirmed eligible, included in the study, completing follow-up, and analysed | 6-7 |
| | | (b) Give reasons for non-participation at each stage | |
| | | (c) Consider use of a flow diagram | Figure 1 |
| Descriptive data | 14* | (a) Give characteristics of study participants (eg demographic, clinical, social) and information on exposures and potential confounders | table 1 |
| | | (b) Indicate number of participants with missing data for each variable of interest | Throughout including tables |
| | | (c) *Cohort study*—Summarise follow-up time (eg, average and total amount) | 6-7 |
| Outcome data | 15* | *Cohort study*—Report numbers of outcome events or summary measures over time | *6-10* |
| | | *Case-control study*—Report numbers in each exposure category, or summary measures of exposure | |
| | | *Cross-sectional study*—Report numbers of outcome events or summary measures | |
| Main results | 16 | (*a*) Give unadjusted estimates and, if applicable, confounder-adjusted estimates and their precision (eg, 95% confidence interval). Make clear which confounders were adjusted for and why they were included | 13 |
| | | (*b*) Report category boundaries when continuous variables were categorized | |
| | | (*c*) If relevant, consider translating estimates of relative risk into absolute risk for a meaningful time period | |
| Other analyses | 17 | Report other analyses done—eg analyses of subgroups and interactions, and sensitivity analyses | Appendix |
| **Discussion** | | | |
| Key results | 18 | Summarise key results with reference to study objectives | 15-16 |
| Limitations | 19 | Discuss limitations of the study, taking into account sources of potential bias or imprecision. Discuss both direction and magnitude of any potential bias | 18 |
| Interpretation | 20 | Give a cautious overall interpretation of results considering objectives, limitations, multiplicity of analyses, results from similar studies, and other relevant evidence | 15-18 |
| Generalisability | 21 | Discuss the generalisability (external validity) of the study results | 18 |
| **Other information** | | | |
| Funding | 22 | Give the source of funding and the role of the funders for the present study and, if applicable, for the original study on which the present article is based | 20 |

*Give information separately for cases and controls in case-control studies and, if applicable, for exposed and unexposed groups in cohort and cross-sectional studies.

**Note:** An Explanation and Elaboration article discusses each checklist item and gives methodological background and published examples of transparent reporting. The STROBE checklist is best used in conjunction with this article (freely available on the Web sites of PLoS Medicine at http://www.plosmedicine.org/, Annals of Internal Medicine at http://www.annals.org/, and Epidemiology at http://www.epidem.com/). Information on the STROBE Initiative is available at www.strobe-statement.org.



For peer review only - http://bmjopen.bmj.com/site/about/guidelines.xhtml

For peer review only

BMJ Open

# Prevalence of insufficient weight loss five years after Roux-en-Y gastric bypass: Prevalence, metabolic consequences and prediction estimates -A prospective registry study

| | |
|---|---|
| Journal: | *BMJ Open* |
| Manuscript ID | bmjopen-2020-046407.R1 |
| Article Type: | Original research |
| Date Submitted by the Author: | 01-Feb-2021 |
| Complete List of Authors: | Brissman, Markus; KI CLINTEC, Department of Clinical Science, Intervention and Technology, Division of Pediatrics; Karolinska University Hospital, Allied Health Professionals Function, Occupational Therapy & Physiotherapy<br>Beamish, Andrew; University of Gothenburg Institute of Clinical Sciences, Department of Surgical Research; Swansea University Medical School<br>Olbers, Torsten; Linkoping University Department of Biomedical and Clinical Sciences; Vrinnevi Hospital in Norrkoping, Department of surgery<br>Marcus, Claude; KI CLINTEC, Department of Clinical Science, Intervention and Technology, Division of Pediatrics, |
| <b>Primary Subject Heading</b>: | Surgery |
| Secondary Subject Heading: | Surgery |
| Keywords: | SURGERY, Lipid disorders < DIABETES & ENDOCRINOLOGY, DIABETES & ENDOCRINOLOGY |
| | |

SCHOLARONE™
Manuscripts

*I, the Submitting Author has the right to grant and does grant on behalf of all authors of the Work (as defined in the below author licence), an exclusive licence and/or a non-exclusive licence for contributions from authors who are: i) UK Crown employees; ii) where BMJ has agreed a CC-BY licence shall apply, and/or iii) in accordance with the terms applicable for US Federal Government officers or employees acting as part of their official duties; on a worldwide, perpetual, irrevocable, royalty-free basis to BMJ Publishing Group Ltd ("BMJ") its licensees and where the relevant Journal is co-owned by BMJ to the co-owners of the Journal, to publish the Work in this journal and any other BMJ products and to exploit all rights, as set out in our licence.*

*The Submitting Author accepts and understands that any supply made under these terms is made by BMJ to the Submitting Author unless you are acting as an employee on behalf of your employer or a postgraduate student of an affiliated institution which is paying any applicable article publishing charge ("APC") for Open Access articles. Where the Submitting Author wishes to make the Work available on an Open Access basis (and intends to pay the relevant APC), the terms of reuse of such Open Access shall be governed by a Creative Commons licence – details of these licences and which Creative Commons licence will apply to this Work are set out in our licence referred to above.*

*Other than as permitted in any relevant BMJ Author's Self Archiving Policies, I confirm this Work has not been accepted for publication elsewhere, is not being considered for publication elsewhere and does not duplicate material already published. I confirm all authors consent to publication of this Work and authorise the granting of this licence.*

For peer review only

# Prevalence of insufficient weight loss five years after Roux-en-Y gastric bypass: Prevalence, metabolic consequences and prediction estimates
# -A prospective registry study

Brissman, M. 1,2, Beamish, A. J. 3,4, Olbers, T. 5,6, Marcus, C. 1

1. Department of Clinical Science, Intervention and Technology, Division of Pediatrics, Karolinska Institute, Huddinge, Sweden
2. Allied Health Professionals Function, Occupational Therapy & Physiotherapy, Karolinska University Hospital, Stockholm, Sweden
3. Department of Surgical Research, Institute of Clinical Sciences, Gothenburg University, Gothenburg, Sweden.
4. Swansea University Medical School, Swansea, United Kingdom of Great Britain and Northern Ireland.
5. Department of Biomedical and Clinical Sciences and Wallenberg Center for Molecular Medicine, Linkoping University, Linkoping, Sweden.
6. Department of Surgery, Vrinnevi hospital, Norrkoping, Sweden

Corresponding author: Markus Brissman, Blickagången 6A, 141 57 Huddinge, Sweden.
Markus.brissman@ki.se

Word Count: 3286

Key words: Bariatric surgery, treatment failure, obesity-related comorbidity, prediction algorithms

For peer review only

Strengths and limitations of this study

- A large prospective cohort of nearly 6000 patients from bariatric surgery centers with a minimum of 60% retention rate at year five after bariatric surgery.
- Pre-defined thresholds of surgical treatment failure and cardiometabolic health were applied.
- The prediction model of surgical treatment failure was cross-validated using partial data, however, further validation of an unrelated cohort is preferable.
- Data originates from the whole of Sweden; thus generalizability may be limited to countries with similar ethnic diversity.

For peer review only


For peer review only - http://bmjopen.bmj.com/site/about/guidelines.xhtml

Abstract

**Objective:** The study aimed to investigate the heterogeneity of weight loss five years after RYGB and the association with cardiometabolic health as well as to model prediction estimates of surgical treatment failure.

**Design:** Retrospective analysis of prospectively collected data from the Scandinavian Obesity Surgery Registry (SOReg).

**Setting:** 29 surgical units from the whole of Sweden contributed data. Inclusion was restricted to surgical units with a retention rate of >60% five years post-surgery.

**Participants:** 10633 patients were extracted from SOReg. In total 5936 participants were included in the final sample, 79·1% females. The mean age of participants before surgery was 39·4±9 years and mean body mass index (BMI) 42·9±5·1. 2322 were excluded (death before the 5-year follow-up (n=148), other types of surgery or reoperations (n=637), age at surgery <18 or >55 years (n=1329), pre-surgery BMI <35kg/m$^2$ (n=208)). In total 2375 (29%) of eligible individuals were lost to the 5-year follow-up.

**Main Outcome:** The occurrence of surgical treatment failure five years after surgery was based on the three previously published definitions: percent excess BMI loss <50%, total weight loss <20%, or BMI >35 where initial BMI was <50, or >40 where initial BMI was >50. In addition, we report the association between surgical treatment failure and biochemical markers of obesity-

For peer review only

related comorbidity. We also developed predictive models to identify patients with a high risk of surgical treatment failure five years post-surgery.

**Results:** In total, 23·1% met at least one definition of surgical treatment failure at year five which was associated with (adjusted odds ratio [OR] with 95% confidence interval [95%CI]): Type 2 diabetes (T2D, OR=2·1; 95%CI 1·6 to 2·7), dyslipidemia (OR=1·8; 95%CI 1·6 to 2·1), and hypertension (OR=1·9; 95%CI 1·6 to 2·2). Surgical treatment failure at five years was predicted by combined demographic and anthropometric measures from baseline, one and two years post-surgery (area under the curve=0·874).

**Conclusion:** LRYGB leads to a marked and sustained weight loss with improvement of obesity-related comorbidity in most patients. However, 23% met at least one definition of surgical treatment failure, which was associated with a greater risk of relapse and a higher incidence of T2D, dyslipidemia and hypertension five years after surgery. Poor Initial weight loss and early weight regain are strong predictors of long-term treatment failure and may be used for early identification of patients who require additional weight loss support.

For peer review only

## Introduction

Obesity is a heterogeneous disease [1] associated with several comorbid conditions, which ultimately increases the risk of all-cause mortality [2]. Bariatric surgery is the most effective treatment for severe obesity. Long-term follow-up studies of Roux-en-Y Gastric Bypass (RYGB) show excellent results at the group level in reductions in weight, morbidity, and mortality compared with non-surgical treatment [3-5]. In Sweden, approximately 5500 bariatric operations are performed annually and, until 2014, the technique was almost exclusively RYGB [6].

Weight loss after surgery is typically achieved during the first and second year, followed by weight maintenance or moderate regain 5-10 years after surgery [7]. However, despite good overall results, the response and durability of surgically induced weight loss are heterogenous [8-10], and surgical treatment failure has been recognized as a potential clinical problem [11-13].

The prevalence of surgical treatment failure is unclear, largely because an all-encompassing, unambiguous definition remains elusive [11-14]. In a landmark controlled study by Adams et al. [5], based on 418 RYGB patients, 30% of participants experienced <20% of total body weight loss at 12 years after RYGB.

It is still unclear to which extent cardiometabolic improvements after bariatric surgery depends on the degree of weight loss. Long-term studies have reported temporally declining rates of remission from obesity-related comorbidities [5,15] and the rate of relapse, especially for type 2


For peer review only - http://bmjopen.bmj.com/site/about/guidelines.xhtml

diabetes (T2D), has rather been attributed to pre-surgery disease duration and progression than to insufficient weight loss [5,16]. Although an association between T2D relapse and weight regain has been suggested in some studies [17-19], others have not found any association between the degree of long-term weight loss and cardiometabolic outcome [20-22]. The annual summary of the Scandinavian Obesity Surgery Registry (SOReg) recently described an association between baseline T2D and inadequate post-operative weight loss [23].

In this study, based on a large cohort of patients prospectively collected in SOReg [6], we report on the heterogeneity of weight loss outcome, focusing primarily on the occurrence of surgical treatment failure five years after surgery, according to any of three published definitions. We also report the association between surgical treatment failure and cardiometabolic disease and we present predictions of surgical treatment failure based on background data and weight development during the first two years after RYGB.

## Methods

### Data Source

The data source for this study was SOReg, a Swedish nationwide registry that began collecting data in 2007; from 2011, the registry covered 95-99% of all bariatric surgery performed in Sweden. Between 2007 and 2011, RYGB constituted 96-97% of all bariatric surgery performed. Data was retrieved in accordance with the study protocol. For this retrospective analysis, data


For peer review only - http://bmjopen.bmj.com/site/about/guidelines.xhtml

For peer review only

were requested for all patients from surgical units and yearly cohorts that had a five-year retention rate of ≥60%. Data covered demographics, anthropometrics, pharmacological treatment, obesity-related comorbidity, biochemical markers and blood pressure at four time points: before surgery (baseline), and at one, two and five years after surgery.

## Participants

In total, 29 surgical units contributed data to the study through the SOReg database, ranging in number from 1 to 1643 patients, and data on 10633 unique patients were extracted. Exclusion was performed in iteration steps and a total of 4697 patients were excluded. The participants included in this study underwent RYGB during 2007-2011, 84·3% had BMI reported for all time points. Missing data on BMI totaled 13·2% at either the one- or two-year follow-up, and 2·5% at both the one- and two-year follow-ups. The follow-up modality at the five-year follow-up was a clinical visit (44·7%), telephone consultation (36·6%), e-mail/letter (18·3%), or unspecified (0·5%). The follow-up modality at the one- and two-year follow-ups are presented in detail in eTable1 in the Supplement. Figure 1 shows the flowchart of the study participants.

## Loss to follow-up analysis

A comparison of baseline characteristics between the study participants and those lost to follow-up revealed that lost participants had a younger age, a higher BMI and a male predomination. A detailed comparison appears in eTable 2 in the Supplement.

For peer review only

## Definitions

Surgical treatment failure

Surgical treatment failure was assessed and defined as meeting at least one of three definitions five years after surgery: i) <50% excess BMI loss (%EBMIL), ii) <20% total weight loss (%TWL), and iii) BMI >35 kg/m$^2$ where baseline was <50 kg/m$^2$, or >40 kg/m$^2$ if baseline BMI was >50 kg/m$^2$. These definitions have been used elsewhere [11,24] and, taken together, provide a means to define failure for patients within different weight categories.

%EBMIL was calculated as ((baseline BMI – year five BMI)/(baseline BMI - 25))*100

%TWL was calculated as ((baseline BMI – year five BMI)/baseline BMI)*100

Two trajectories - inadequate weight loss and weight regain - can be defined that lead to long-term surgical treatment failure. Inadequate weight loss has been quantified during the first 6-12 months after surgery [25], and weight regain has typically been described as an increase above a specified threshold [12,13].

In this paper inadequate weight loss was defined as <25%TWL from baseline to one year post-surgery, similar to the 25$^{th}$ percentile presented by Manning et al. [25].

Early weight regain was defined as any absolute weight gain, expressed in kilograms, occurring between year one and two after surgery. This definition generated two groups. Long-term weight regain, defined according to Odom et al. [26] in three groups: >15% regain of BMI nadir,


For peer review only - http://bmjopen.bmj.com/site/about/guidelines.xhtml

0·1-15% regain of BMI nadir, and no weight regain, to five years post-surgery. These definitions were used to capture early weight regain as a predictive measurement of long-term surgical treatment failure, and to differentiate between the normally occurring fluctuation of body weight in the maintenance phase and the potentially harmful weight regain previously suggested [18,19].

For calculations, BMI nadir was accepted as the lowest measured weight at either the one or two year follow-up. In the case of missing data from one of those time points the observed measurement was taken as the nadir.

## Obesity-related comorbidities and metabolic markers

It is mandatory to report obesity-related comorbidities (e.g., T2D, dyslipidemia, hypertension) [6] requiring pharmacological treatment in SOReg, and data were available for 88-100% included individuals depending on timepoint (full description in eTable 3 in the Supplement).

Blood pressure and biochemical markers, such as low-density lipoprotein (LDL), high-density lipoprotein (HDL), triglycerides (TG), fasting glucose, and glycated hemoglobin (HbA1c), are optional to report. Data were available data from 34-73% included participants (eTable 3 in the Supplement).

Changes in blood pressure and biochemical markers were compared, stratified by surgical treatment failure at the five-year follow-up, and by pharmacological treatment at baseline.



For peer review only - http://bmjopen.bmj.com/site/about/guidelines.xhtml

Additionally a broader classification of disease traits was generated, similar to that previously described [5,27], by compiling a disease-specific biochemical marker above a cut-off (eAppendix 1 in the Supplement., in combination with pharmacological treatment. This classification was applied at all time points and used to assess prevalence and change over time. Thus, six groups were generated: participants without disease traits at baseline were classified “disease-free” if no disease trait was evident at any time point, “intermittent” if disease-free at both baseline and five-year follow-up, but not in between, and “incidence” where a disease trait developed during the five-year follow-up period. Participants with a disease trait at baseline were classified “remission” if no disease trait was evident at five-year follow-up, “relapse” if disease-free at year one, two, or both, but not at year five, and “no remission” where at least one disease trait was evident at all time points.

For clarity, the compiled disease traits are hereafter referred to as T2D, dyslipidemia and hypertension.

## Statistics

All statistical analyses were performed using SPSS v.24 (IBM Corp. USA) and STATA IC 15.1 (StataCorp USA). Descriptive statistics are presented as mean ± standard deviation (±sd), or as a percentage (%), unless otherwise specified.

Characteristics were compared between those lost to follow-up (eTable 2 in the Supplement) and those included in the analysis, as well as according to surgical treatment failure status (eTable 4 in the Supplement), using independent t- and chi-square tests.


For peer review only - http://bmjopen.bmj.com/site/about/guidelines.xhtml

We described the prevalence and change in cardiometabolic disease and assessed the odds associated with surgical treatment failure using logistic regression, first using a crude model (data not shown) and then multivariable models (separate, compiled or additive for each definition of surgical treatment failure) in which we adjusted for sex, age and BMI at baseline, and corresponding cardiometabolic disease. Results are presented as odds ratios (OR) with 95% confidence intervals (95%CI).

In addition, we used logistic regression to predict the probability of meeting at least one definition of surgical treatment failure, which we considered dichotomously (1 = surgical treatment failure, 0 = otherwise). Our predictions used sex, baseline, age, BMI and %TWL for the first year and change in weight (kg) for the second year. We measured performance by calculating the receiver operating characteristic (ROC) curve and the corresponding area under the curve (AUC) and by using cross-validation (leave 10%, k = 10 replicates).

Finally, several sensitivity analyses were undertaken for the primary endpoint (i.e. surgical treatment failure), which can be found in eAppendix 2 in the Supplement.

The significance level was set to 0·05 for all analyses (two-tailed), and p-values are reported with three decimals.

Patient and public involvement

Patients nor the public were involved in the conduct of this study.

## Results


For peer review only - http://bmjopen.bmj.com/site/about/guidelines.xhtml

In total, 5936 patients (79·1% female), aged 18-55 years, who had undergone LRYGB from 2007 to 2012, were included in the final sample (Figure 1). At baseline, the mean age was 39·4±9·0 years and BMI was 42·9±5·1 kg/m$^2$. Patient characteristics are presented in Table 1. At year five, overall mean BMI was 30·4±5·2, mean weight loss 35·8 ±13·8 kg, BMI loss 12·6±4·7 kg/m$^2$, %EBMIL 72·2±25·2% and %TWL 29·1±9·8%.

Inadequate weight loss (i.e. <25%TWL from baseline to year one) was identified in 17·1% of 5596 participants with available data.

Early weight regain (between year one and two) was identified in 38·7% of 5010 participants with available data, with a mean increase of 4·5±3·9 kg (range 1 to 38 kg), compared with a mean decrease of 4·4±5·1 kg (range 66 to 0 kg) in the no regain group.

Long-term weight change between nadir and five year follow-up was distributed as follows: >15% regain (+17·7±7·2 kg, range 7 to 101 kg) in 19·9% of participants, 0·1-15% regain (+5·7±3·5 kg, range 0 to 19 kg) in 59·3%, and no weight regain (-5·0±5·2 kg, range -36 to 0 kg) in 20·8%.

Overall, the prevalence of meeting at least one of the three definitions of surgical treatment failure five years after LRYGB was 23·1% (n=1371). The distribution between the three definitions was 19·2% (n=1138) for <50%EBMIL, 17·0% (n=1010) for <20%TWL, and 14·1% (n=835) for BMI >35 or >40 kg/m$^2$. There was substantial overlap, 39·8% (n=545) meeting all three definitions and 38·1% (n=522) meeting two of the three definitions (Figure 2).


For peer review only - http://bmjopen.bmj.com/site/about/guidelines.xhtml

Surgical treatment failure was more common among patients with inadequate weight loss (60% vs. 15·4%, p<·001) and early weight regain (33·8% vs. 15·6%, p<·001). Comparing long-term weight regain, the proportion meeting criteria for failure was highest in participants with >15% regain from nadir (46·7%), followed by 0·1-15% (21·1%), and no regain (5·1%), (p<·001). Patients with no long-term weight regain but surgical treatment failure (n=59) had higher baseline BMI (48·5 vs. 43·1, p<·001) and lower %TWL at one- and two-year follow-up (-18·0% vs. -30·5% and -18·1% vs. -32·3%, respectively, both p<·001).

Cardiometabolic disease

Biochemical and physiological measures improved following surgery in participants with and without surgical treatment failure. Mean values, stratified by surgical treatment failure and baseline pharmacological treatment, are shown from baseline to year five in eFigures 1.a-g and 2.a-g in the Supplement.

Overall, the prevalence of cardiometabolic disease decreased from baseline to 5 years: T2D from 15·1% (n=896) to 6·4% (n=380), dyslipidemia from 60·7% (n=3603) to 16·4% (n=974), and hypertension from 28·4% (n=1683) to 18·9% (n=1124). The rates of being disease-free, incident and intermittent disease, as well as remission, relapse and no remission, varied between surgical and non-surgical treatment failure (Table 2).

Logistic regression (adjusted for sex, age, BMI, and corresponding cardiometabolic disease at baseline) confirmed an association between surgical treatment failure and cardiometabolic



For peer review only - http://bmjopen.bmj.com/site/about/guidelines.xhtml

disease at year five: T2D, OR=2·10 (95%CI 1·61 to 2·75); dyslipidemia, OR=2·50 (95%CI 2·14 to 2·92); and hypertension, OR=1·85 (95%CI 1·55 to 2·21). Individual definitions were similarly associated with cardiometabolic disease (eTable 5 in the Supplement). The combined effect of fulfilling one, two, or three of the definitions is presented in eTable 6 in the Supplement. Predicted probability of cardiometabolic disease plotted against continuous %EBMIL, %TWL, and BMI at year five is illustrated in eFigures 3-5.a-c in the Supplement.

Inadequate weight loss during year one was significantly associated with T2D (OR=1·84; 95%CI 1·38 to 2·45), dyslipidemia (OR=1·89; 95%CI 1·59 to 2·25), and hypertension (OR=1·61; 95%CI 1·32 to 1·96). Late weight regain (≥15% regain from nadir) was significantly associated with dyslipidemia (OR=1·64; 95%CI 1·31 to 2·05) and hypertension (OR=1·41; 95%CI 1·10 to 1·81), but not T2D (OR=1·25; 95%CI 0·84 to 1·88).

Predicting surgical treatment failure

The estimated regression coefficients and OR are presented in Table 3. Given age, sex and baseline BMI and %TWL from baseline to the one-year follow-up, and change in weight (kg) between one- and two-year follow-up, the predicted probability of surgical treatment failure five years after surgery is given by:

P(surgical treatment failure) = exp(a)/(1+(a)) with a = - 1·1 + 0·00545*(sex male=0 female=1) + 0·00299*(age at surgery, years) + 0·14949*(baseline BMI) + 0·22310*(%TWL year one) + 0·15982*(weight change year one to year two (kg)). Examples of the probability calculation are presented in eAppendix 3 in the Supplement.


For peer review only - http://bmjopen.bmj.com/site/about/guidelines.xhtml

As depicted in Figure 3, this simple model provided a good prediction (AUC = 0·8743).

## Discussion

For peer review only

This analysis of prospectively collected data on 5963 adults who underwent primary LRYGB surgery, revealed that almost one in four participants fulfilled at least one of the three applied definitions of surgical treatment failure, five years after surgery. Surgical treatment failure was associated with a negative effect on cardiometabolic health: lower rate of remission and more frequent relapse and incidence of T2D, dyslipidemia and hypertension. Each definition of surgical treatment failure and weight regain was independently associated with cardiometabolic health. In total, 9·2% of the study population fulfilled the criteria for all of the three definitions [11,24] of surgical treatment failure and they provided a very strong association with T2D, dyslipidemia and hypertension.

The extent to which insufficient weight loss and weight regain affect cardiometabolic outcome is unclear, both confirmative [13,18,19,28-30] and negative [20-22,31,32] findings have been reported. In the present study inadequate weight loss during year one and weight regain during year two were investigated. Both were found to be associated with cardiometabolic outcomes, however, both were in the present study viewed as prerequisites for surgical treatment failure, which in turn was associated with a less favorable metabolic profile five years after surgery, regardless


For peer review only - http://bmjopen.bmj.com/site/about/guidelines.xhtml

of whether or not patients were taking T2D, dyslipidemia, or hypertension medications prior to surgery.

Early identification of those with a high risk of long-term surgical treatment failure may facilitate additional weight loss support [33-35]. Unfortunately neither we, nor others, have been able to build a sufficiently reliable model using exclusively pre-surgical characteristics [36]. However, our results indicate that long-term surgical treatment failure can, with good accuracy (AUC = 0·8743), be predicted by sex, age and BMI at baseline, together with %TWL during year one and weight change during year two. We found that %TWL during year one was the strongest predictor of surgical treatment failure. Similarly the initial six month weight loss predicts the 24-month weight loss [25]. Of note, we found that pre-surgical T2D, dyslipidemia and hypertension were associated with surgical treatment failure, a finding that may warrant further research as the associations could be dependent on both behavioral and physiological factors.

The present study terms long-term poor weight loss after surgery as surgical treatment failure. This wording should not be interpreted to mean that the surgical procedure failed, but rather that the therapy alone was insufficient to produce the required degree of long-term weight loss. This reasoning should not be surprising given the heterogeneous nature of obesity, as any standardized treatment is likely to result in a spectrum of outcomes. Despite that, bariatric surgery has remained a stand-alone treatment. This is contrary to bariatric surgery guidelines suggesting active treatment of patients with poor weight outcome [37]. In addition patients have


For peer review only - http://bmjopen.bmj.com/site/about/guidelines.xhtml
For peer review only

also expressed a need for more extensive follow-up [38]. Recognizing this, bariatric surgery would likely benefit from the application of the multidisciplinary and multimodal approach that has evolved in other fields of disease, such as cancer care, where for decades surgery has been integrated into multimodal treatment pathways, alongside chemotherapies and radiation therapies. It has been shown that behavioral support [35] and pharmacological treatment [34] can improve the outcome after surgery, indicating potential for additive, perhaps even synergistic effects of combination therapies. However, as a consequence of the disintegrated follow-up after surgery, it is still unclear to which extent outcome after bariatric surgery can be optimized by means of adjuvant treatment.

Strengths of this study include SOReg's prospective collection of data from the whole of Sweden, with broad national coverage. This was demonstrated by the inclusion of nearly 6000 patients from the database of centers with a ≥60% retention rate five years after LRYGB, providing a large and robust data set permitting subgroup analysis. All patients included in the final sample had undergone LRYGB. This constituted 95-97.5% of all bariatric surgery performed between 2007 and 2011 in Sweden, thus reducing possible bias in patient selection for different surgical procedures.

There are also some limitations. Although the impact of surgical treatment failure on metabolic health is substantial, it does not account for all comorbidity seen at the five-year follow-up. Other factors, such as disease duration before surgery, are also of importance but such information was not available in this study. Neither was information on psychological disorders

For peer review only

available, thus limiting the possibility to evaluate and include such factors in the prediction model.

Missing data analysis revealed that rates of surgical treatment failure at year one and two were higher in the 28·6% that were lost to follow-up year five, indicating that the actual proportion of surgical treatment failure may be higher than what the results suggests. In addition, there was a difference in weight loss between the modes of follow-up, possibly implying bias of self-reported data. Similarly, a statistical limitation of note is that we compiled disease-specific traits where missing data are implicitly treated as zeroes. For example, the estimated effects may be diluted (biased towards zero) because the comparison is actual ones vs. a mixture of zeroes and ones. Thus, both the overall prevalence of surgical treatment failure and cardiometabolic disease may be underestimated.

The developed prediction model for long-term surgical treatment failure was cross-validated using partial data and can readily be applied to countries with similar cultural and ethnic settings as in northern Europe. However, further validation of an unrelated cohort is preferable, and further devolvement of the model may be required to encompass ethnic diversity.

Unsuccessful surgical treatment result is difficult to define and a large number of definitions and time points have been used [11-14]. Our results would probably have been slightly modified if we had used other definitions. However, the strong associations between surgical treatment failure, as defined in the present study, and cardiometabolic health may support their clinical usefulness.


For peer review only - http://bmjopen.bmj.com/site/about/guidelines.xhtml

## Conclusion

RYGB is associated with improvement of obesity-related comorbidity. However, 23% of the patients developed surgical treatment failure five years after surgery, which was associated with a markedly increased risk of cardiometabolic disease. Initial weight loss and early weight regain were strong predictive markers that can be used for the early identification of patients with a high risk of long-term failure. This study underlines the need for long-term follow-up of patients undergoing bariatric surgery by a multidisciplinary team and improved additional behavioral and pharmacological treatment post-surgery are warranted.

## Author contribution

MB and CM conceptualized the study, MB performed data and statistical analyses and drafted the manuscript. All authors (MB, AJB, TO, CM) contributed to result interpretation and critically revised and approved the final version of the manuscript. MB and CM had full access to all the data in the study and takes responsibility for the integrity of the data and the accuracy of the data analysis.

## Funding

This work was funded by the Swedish order of Freemasons (grant no: not applicable), the Swedish Heart- Lung Foundation (grant no: 20180581), the Samariten foundation for pediatric research (grant no: not applicable) and Anna-Lisa and Arne Gustafssons foundation (grant no: not applicable).

For peer review only

Competing interests

TO declares participation in advisory board for J&J and Novo Nordisk and reimbursement for lectures and education activities. All fees to institution.

CM has received research grants from Novo Nordisk, Sigrid THX AB and salaries as medical advisor for Itrim AB and Weight Watchers Int.

MB and AJB declares no conflict of interest.

Disclaimer

The funders of this study had no part in study design, collection, analysis or interpretation of data, nor in the writing of the report or in the decision to submit the paper for publication. The corresponding author had full access to the data and had final responsibility for the decision to submit for publication.

Transparency statement

The lead author (MB) and the guarantor (CM), affirms that the manuscript is an honest, accurate, and transparent account of the study being reported; that no important aspects of the study have been omitted; and that any discrepancies from the study as originally planned have been explained.

Dissemination of the study results to patients and patient organizations is not applicable.

Patient consent for publication

Not required.

For peer review only



For peer review only - http://bmjopen.bmj.com/site/about/guidelines.xhtml

Ethical approval

This study was approved by the Stockholm ethical board (2017/1793-31).

Data sharing

No additional data available from the authors. Original data may be requested from the Scandinavian Obesity Surgery Registry (https://www.ucr.uu.se/soreg/in-english)

For peer review only

Reference

1. Gonzalez-Muniesa P, Martinez-Gonzalez MA, Hu FB, et al. Obesity. *Nat Rev Dis Primers* 2017; **3**: 17034.
2. Aune D, Sen A, Prasad M, et al. BMI and all cause mortality: systematic review and non-linear dose-response meta-analysis of 230 cohort studies with 3.74 million deaths among 30.3 million participants. *BMJ* 2016; **353**: i2156.
3. Sjostrom L. Review of the key results from the Swedish Obese Subjects (SOS) trial - a prospective controlled intervention study of bariatric surgery. *Journal of internal medicine* 2013; **273**(3): 219-34.
4. Gloy VL, Briel M, Bhatt DL, et al. Bariatric surgery versus non-surgical treatment for obesity: a systematic review and meta-analysis of randomised controlled trials. *BMJ* 2013; **347**: f5934.
5. Adams TD, Davidson LE, Litwin SE, et al. Weight and Metabolic Outcomes 12 Years after Gastric Bypass. *N Engl J Med* 2017; **377**(12): 1143-55.
6. Hedenbro JL, Naslund E, Boman L, et al. Formation of the Scandinavian Obesity Surgery Registry, SOReg. *Obes Surg* 2015; **25**(10): 1893-900.
7. Buchwald H, Avidor Y, Braunwald E, et al. Bariatric surgery: a systematic review and meta-analysis. *JAMA* 2004; **292**(14): 1724-37.
8. de Hollanda A, Ruiz T, Jimenez A, Flores L, Lacy A, Vidal J. Patterns of Weight Loss Response Following Gastric Bypass and Sleeve Gastrectomy. *Obes Surg* 2015; **25**(7): 1177-83.


For peer review only - http://bmjopen.bmj.com/site/about/guidelines.xhtml

9. Azagury D, Mokhtari TE, Garcia L, et al. Heterogeneity of weight loss after gastric bypass, sleeve gastrectomy, and adjustable gastric banding. *Surgery* 2019; **165**(3): 565-70.
10. Courcoulas AP, King WC, Belle SH, et al. Seven-Year Weight Trajectories and Health Outcomes in the Longitudinal Assessment of Bariatric Surgery (LABS) Study. *JAMA Surg* 2018; **153**(5): 427-34.
11. Mann JP, Jakes AD, Hayden JD, Barth JH. Systematic review of definitions of failure in revisional bariatric surgery. *Obes Surg* 2015; **25**(3): 571-4.
12. Karmali S, Brar B, Shi X, Sharma AM, de Gara C, Birch DW. Weight recidivism post-bariatric surgery: a systematic review. *Obes Surg* 2013; **23**(11): 1922-33.
13. King WC, Hinerman AS, Belle SH, Wahed AS, Courcoulas AP. Comparison of the Performance of Common Measures of Weight Regain After Bariatric Surgery for Association With Clinical Outcomes. *JAMA* 2018; **320**(15): 1560-9.
14. Bonouvrie DS, Uittenbogaart M, Luijten A, van Dielen FMH, Leclercq WKG. Lack of Standard Definitions of Primary and Secondary (Non)responders After Primary Gastric Bypass and Gastric Sleeve: a Systematic Review. *Obes Surg* 2019; **29**(2): 691-7.
15. Sjostrom L, Lindroos AK, Peltonen M, et al. Lifestyle, diabetes, and cardiovascular risk factors 10 years after bariatric surgery. *N Engl J Med* 2004; **351**(26): 2683-93.
16. Pories WJ, Swanson MS, MacDonald KG, et al. Who would have thought it? An operation proves to be the most effective therapy for adult-onset diabetes mellitus. *Annals of surgery* 1995; **222**(3): 339-50; discussion 50-2.
17. Shah M, Simha V, Garg A. Review: long-term impact of bariatric surgery on body weight, comorbidities, and nutritional status. *J Clin Endocrinol Metab* 2006; **91**(11): 4223-31.
18. DiGiorgi M, Rosen DJ, Choi JJ, et al. Re-emergence of diabetes after gastric bypass in patients with mid- to long-term follow-up. *Surg Obes Relat Dis* 2010; **6**(3): 249-53.
19. Laurino Neto RM, Herbella FA, Tauil RM, Silva FS, de Lima SE, Jr. Comorbidities remission after Roux-en-Y Gastric Bypass for morbid obesity is sustained in a long-term follow-up and correlates with weight regain. *Obes Surg* 2012; **22**(10): 1580-5.
20. Hawkins RB, Mehaffey JH, McMurry TL, et al. Clinical significance of failure to lose weight 10 years after roux-en-y gastric bypass. *Surg Obes Relat Dis* 2017; **13**(10): 1710-6.
21. Magno F, Sousa PAM, Rodrigues MP, et al. Long term maintenance of glucose and lipid concentrations after Roux-en-Y gastric bypass. *Arch Endocrinol Metab* 2018; **62**(3): 346-51.
22. Aminian A, Jamal M, Augustin T, et al. Failed Surgical Weight Loss Does Not Necessarily Mean Failed Metabolic Effects. *Diabetes Technol Ther* 2015; **17**(10): 682-4.
23. Registry TSOS. Fördjupningsrapport SOReg 2019, diabetes. 2019. https://www.ucr.uu.se/soreg/arsrapporter (accessed 28th oct 2019).
24. Corcelles R, Boules M, Froylich D, et al. Total Weight Loss as the Outcome Measure of Choice After Roux-en-Y Gastric Bypass. *Obes Surg* 2016; **26**(8): 1794-8.
25. Manning S, Pucci A, Carter NC, et al. Early postoperative weight loss predicts maximal weight loss after sleeve gastrectomy and Roux-en-Y gastric bypass. *Surg Endosc* 2015; **29**(6): 1484-91.
26. Odom J, Zalesin KC, Washington TL, et al. Behavioral predictors of weight regain after bariatric surgery. *Obes Surg* 2010; **20**(3): 349-56.
27. Inge TH, Courcoulas AP, Jenkins TM, et al. Five-Year Outcomes of Gastric Bypass in Adolescents as Compared with Adults. *N Engl J Med* 2019; **380**(22): 2136-45.


For peer review only - http://bmjopen.bmj.com/site/about/guidelines.xhtml

28. Zhou K, Wolski K, Malin SK, et al. Impact of Weight Loss Trajectory Following Randomization to Bariatric Surgery on Long-Term Diabetes Glycemic and Cardiometabolic Parameters. *Endocr Pract* 2019; **25**(6): 572-9.
29. Nicoletti CF, de Oliveira BA, de Pinhel MA, et al. Influence of excess weight loss and weight regain on biochemical indicators during a 4-year follow-up after Roux-en-Y gastric bypass. *Obes Surg* 2015; **25**(2): 279-84.
30. Brethauer SA, Aminian A, Romero-Talamas H, et al. Can diabetes be surgically cured? Long-term metabolic effects of bariatric surgery in obese patients with type 2 diabetes mellitus. *Annals of surgery* 2013; **258**(4): 628-36; discussion 36-7.
31. Cohen RV, Rubino F, Schiavon C, Cummings DE. Diabetes remission without weight loss after duodenal bypass surgery. *Surg Obes Relat Dis* 2012; **8**(5): e66-8.
32. Cohen RV, Pinheiro JC, Schiavon CA, Salles JE, Wajchenberg BL, Cummings DE. Effects of gastric bypass surgery in patients with type 2 diabetes and only mild obesity. *Diabetes Care* 2012; **35**(7): 1420-8.
33. Kalarchian MA, Marcus MD. Psychosocial Interventions Pre and Post Bariatric Surgery. *Eur Eat Disord Rev* 2015; **23**(6): 457-62.
34. Nor Hanipah Z, Nasr EC, Bucak E, et al. Efficacy of adjuvant weight loss medication after bariatric surgery. *Surg Obes Relat Dis* 2018; **14**(1): 93-8.
35. Andreu A, Jimenez A, Vidal J, et al. Bariatric Support Groups Predicts Long-term Weight Loss. *Obes Surg* 2020.
36. Livhits M, Mercado C, Yermilov I, et al. Preoperative predictors of weight loss following bariatric surgery: systematic review. *Obes Surg* 2012; **22**(1): 70-89.
37. Mechanick JI, Youdim A, Jones DB, et al. Clinical practice guidelines for the perioperative nutritional, metabolic, and nonsurgical support of the bariatric surgery patient--2013 update: cosponsored by American Association of Clinical Endocrinologists, The Obesity Society, and American Society for Metabolic & Bariatric Surgery. *Obesity (Silver Spring)* 2013; **21 Suppl 1**: S1-27.
38. Parretti HM, Hughes CA, Jones LL. 'The rollercoaster of follow-up care' after bariatric surgery: a rapid review and qualitative synthesis. *Obes Rev* 2019; **20**(1): 88-107.


For peer review only - http://bmjopen.bmj.com/site/about/guidelines.xhtml

**Table 1. Baseline characteristics of the study population**

| | n | mean (sd) |
|---|---|---|
| Age at surgery | 5936 | 39·4 (9·0) |
| Sex, no. % female | 5936 | 79·1% |
| Height, cm | 5936 | 168·8 (8·9) |
| Weight, kg | 5936 | 122·8 (20·0) |
| Body mass index at surgery, kg/$m^2$ | 5936 | 42·9 (5·1) |
| **Glucose Metabolism** | | |
| Glucose, mmol/l | 2861 | 5·9 (1·9) |
| HbA1c, mmol/mol | 4168 | 40·6 (11·4) |
| Pharmacological diabetes treatment, no. (%) | 5936 | 675 (11·4%) |
| Diabetes type 2[A], no. (%) | 5936 | 896 15·1% |
| **Lipids** | | |
| High-density lipoprotein, mmol/l | 4188 | 1·2 (0·4) |
| Low-density lipoprotein, mmol/l | 4110 | 3·1 (0·9) |
| Triglycerides, mmol/l | 4314 | 1·7 (1·4) |
| Pharmacological dyslipidemia treatment, no. (%) | 5936 | 414 (7·0%) |
| Dyslipidemia[B], no. (%) | 5936 | 3601 67·5% |
| **Physiology** | | |
| Systolic BP, mm/Hg | 2960 | 133 (16) |
| Diastolic BP, mm/Hg | 2960 | 83 (10) |
| Pharmacological hypertension treatment, no. (%) | 5936 | 1158 (19·5%) |
| Hypertension[C], no. (%) | 5936 | 1683 28·4% |

[A] Pharmacologically treated T2D | fasting glucose >7·0mmol/l | HbA1c >48mmol/mol
[B] Pharmacologically treated dyslipidemia | LDL >4·1 | TG > 2·0 | HDL <1mmol/L for males and <1·3mmol/L for females
[C] Pharmacologically treated blood pressure | Systolic- >140mm/Hg or Diastolic blood pressure >90mm/Hg

For peer review only


For peer review only - http://bmjopen.bmj.com/site/about/guidelines.xhtml

**Table 2. Change in cardiometabolic disease status from baseline to five years post-surgery compared between surgical treatment failure (STF) and non-STF.**

| | Type 2 diabetes[A] | | Dyslipidemia[B] | | Hypertension[C] | |
|---|---|---|---|---|---|---|
| | STF n = 1135 | Non-STF n = 3878 | STF n = 1120 | Non-STF n = 3867 | STF n = 1126 | Non-STF n = 3842 |
| **No disease at baseline** | **n = 882** | **n = 3379** | **n = 377** | **n = 1616** | **n = 735** | **n = 2818** |
| *Disease-free* | *97·4%* | *98·5%* | *82·0%* | *87·3%* | *83·9%**** | *91·3%* |
| *Incidence* | *1·6%*** | *0·7%* | *9·5%**** | *4·9%* | *9·9%**** | *4·6%* |
| *Intermittent* | *1·0%* | *0·9%* | *8·5%* | *7·8%* | *6·1%** | *4·0%* |
| | | | | | | |
| **Disease at baseline** | **n = 253** | **n = 499** | **n = 743** | **n = 2251** | **n =391** | **n = 1024** |
| *Remission* | *51·4%**** | *66·5%* | *63·7%**** | *81·1%* | *38·6%**** | *54·6%* |
| *No remission* | *26·1%* | *22·4%* | *17·2%**** | *8·8%* | *37·6%**** | *27·1%* |
| *Relapse* | *22·5%**** | *11·0%* | *19·1%**** | *10·1%* | *23·8%** | *18·3%* |

*indicates a statistically significant difference at p<·05
**indicates a statistically significant difference at p<·010
***indicates a statistically significant difference at p<·001

[A] pharmacologically treated T2D | fasting glucose >7·0mmol/l | HbA1c >48mmol/mol

[B] Pharmacologically treated dyslipidemia | LDL >4·1 | TG > 2·0 | HDL <1mmol/L for males and <1·3mmol/L for females
[C] Pharmacologically treated blood pressure | Systolic- >140mm/Hg or Diastolic blood pressure >90mm/Hg


For peer review only - http://bmjopen.bmj.com/site/about/guidelines.xhtml
For peer review only

**Table 3. Final multivariable model for predicting surgical treatment failure five years after surgery**

| | Beta (B) | S.E. | Wald | p | Exp(B) | 95%Confidence interval |
|---|---|---|---|---|---|---|
| Sex (0=male) | -0·00545 | 0·099 | 0·003 | 0·956 | 0·995 | 0·818-1·209 |
| Age at surgery, years | 0·00299 | 0·005 | 0·361 | 0·548 | 1·003 | 0·993-1·013 |
| BMI at surgery, kg/m$^2$ | 0·14949 | 0·009 | 283·640 | 0·000 | 1·161 | 1·141-1·182 |
| Percentage BMI loss during year one, %TWL | 0·22310 | 0·008 | 794·848 | 0·000 | 1·250 | 1·231-1·269 |
| Change in weight between year one and two, kg | 0·15982 | 0·008 | 382·606 | 0·000 | 1·173 | 1·155-1·192 |
| Intercept | -1·09588 | 0·513 | 4·569 | 0·033 | 0·334 | |

BMI – Body mass index
S.E. – Standard error

For peer review only

**Figure 1. Flowchart of the study participants.**

**Figure 2. Venn diagram of the prevalence of developing surgical treatment failure five years post-surgery according to three definitions: %excess BMI loss (n=1138), BMI >35 or >40 (n=835) and <20% total weight loss (n=1010).**

**Figure 3. Receiver operating characteristic curve with predicted probability of surgical treatment failure, given age, sex and baseline BMI and %TWL from baseline to the one year follow-up and change in weight (kg) between year one and year two follow-ups: area under the curve = 0·8743 (95% confidence interval 0·8630-0·8856).**

For peer review only


For peer review only - http://bmjopen.bmj.com/site/about/guidelines.xhtml

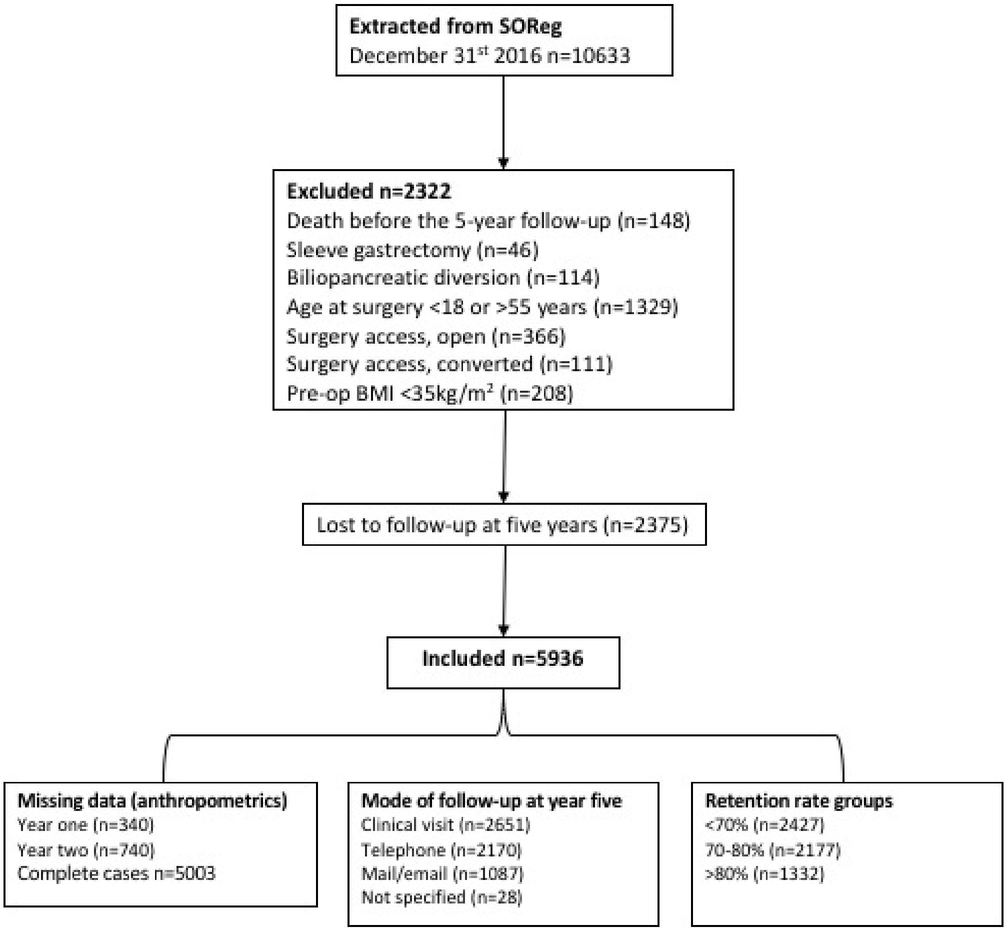


179x165mm (72 x 72 DPI)

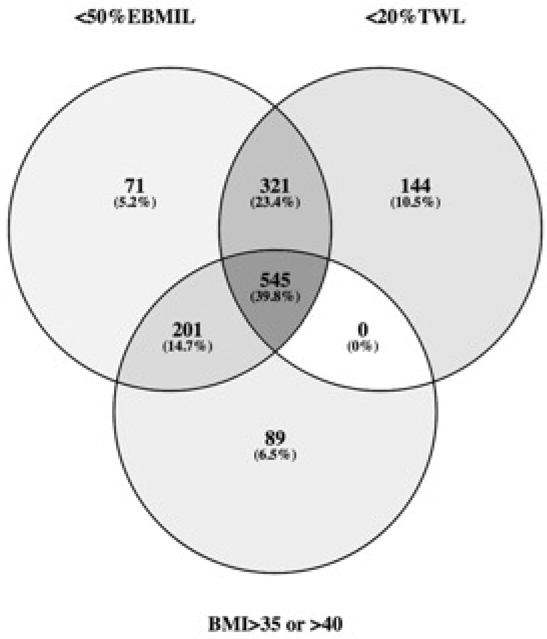


123x123mm (72 x 72 DPI)

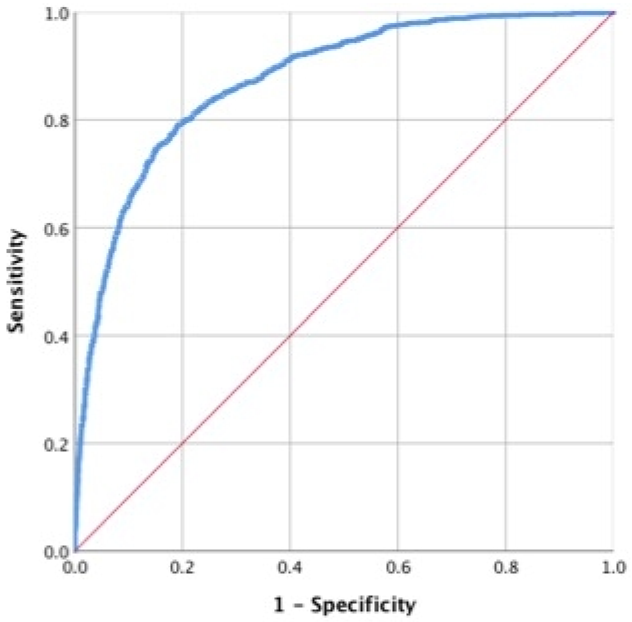


121x111mm (72 x 72 DPI)

Online-only Supplements

**eAppendix 1.** Cut-offs used to define cardiometabolic disease

**eAppendix 2.** Sensitivity analysis

**eAppendix 3.** Examples of risk calculation

**eFigure 1 a-g.** Unadjusted mean participants with pharmacological treatment of dyslipidemia and/or type 2 diabetes or hypertension at baseline, stratified on surgical treatment failure

**eFigure 2 a-g.** Unadjusted mean participants without pharmacological treatment of dyslipidemia and/or type 2 diabetes or hypertension at baseline, stratified on surgical treatment failure

**eFigure 3.a-c.** Predicted probability of type 2 diabetes at year five after surgery plotted over a, percentage weight loss from baseline to year five, b, excess body mass index loss from baseline to year five, c, body mass index at year five

**eFigure 4.a-c.** Predicted probability of dyslipidemia at year five after surgery plotted over a, percentage weight loss from baseline to year five, b, excess body mass index loss from baseline to year five, c, body mass index at year five.

**eFigure 5.a-c.** Predicted probability of Hypertension at year five after surgery plotted over a, percentage weight loss from baseline to year five, b, excess body mass index loss from baseline to year five, c, body mass index at year five.

**eTable 1.** Modality of the one-, two-, and five year follow-ups

**eTable 2.** Comparison of baseline characteristics between included participants and those lost to follow-up at year five

**eTable 3.** Percentage of available data on pharmacological treatment, biochemistry and blood pressure.

**eTable 4.** Comparison of baseline characteristics between surgical treatment failure and non-surgical treatment failure

**eTable 5.** Odds of cardiometabolic disease at year five, separate models for each definitions of surgical treatment failure

**eTable 6.** Odds of Cardiometabolic disease at year five for subjects reaching one, two or three of the three definitions



For peer review only - http://bmjopen.bmj.com/site/about/guidelines.xhtml

## eAppendix 1. Cut-offs used to define cardiometabolic disease

LDL>3.0mmol/L
HDL <1.0mmol/L for males and <1.3mmol/L for females
Triglycerides >2.0mmol/L
Fasting glucose >7.0mmol/L
HbA1c >48mmol/mol
Systolic- >140mmHg or Diastolic blood pressure >90mmHg

Conversion to mg/dL is done by a multiplying factor of 38.67 for LDL and HDL, 88.57 for TG, 18 for fasting glucose and using the formula (0.09148*IFCC) + 2.152 for HbA1c(%).

For peer review only


For peer review only - http://bmjopen.bmj.com/site/about/guidelines.xhtml

## eAppendix 2. Sensitivity analysis

We compared participants lost to follow-up with those not lost and found that the former had a higher prevalence of surgical treatment failure at year one (12.3% vs. 10.3%, p=0.015) and a similar prevalence (13.3% vs. 11.6%) at year two (p=0.081). Within the sample, there was a strong carryover effect, as 73.5% who met the definition of surgical treatment failure in year one and 79.5% in year two also met the definition at year five.

Additionally, participants who visited the clinic for follow-up had a higher prevalence of surgical treatment failure (26.7%) compared with other modes of follow-up (20.2%, p<0.001).

No statistical differences in prevalence of surgical treatment failure at year five were evident between retention groups 60-70%, 70-80% and >80%, (23.8% vs. 23.4% vs. 21.4%) (p=0.235). We found no statistical difference according to the year of surgery (p=0.280), or surgical volume (<50 [23.7%] vs. ≥50 [23.0%] LRYGB per year, p=0.695).

There was a crude difference in the prevalence of surgical treatment failure between males and females (males 31.6% vs. females 20.9%, p<0.001): OR=1.46 (95%CI 1.26-1.69) after baseline adjustments for age, BMI, T2D, hypertension and dyslipidemia. Males experienced a lower %TWL from baseline to all follow-up periods (data not shown).

At baseline, T2D (25.1% vs. 12.4%) and hypertension (44.4% vs. 24.1%) were more common among males (both p<0.001), whereas dyslipidemia was more common among females (61.7% vs. 56.9%, p=0.002).

We also found an association between those who had cardiometabolic disease present at baseline and surgical treatment failure at year five. The presence of T2D at baseline was associated with surgical treatment failure (OR=1.70; 95%CI 1.44-2.00), as was dyslipidemia at baseline (OR=1.30; 95%CI 1.15-1.48) and hypertension at baseline (OR=1.16; 95%CI 1.01-1.34), all adjusted for sex, age and BMI.

In terms of the magnitude of prediction factors, four additional models were assessed, one omitting change in weight between year one and two (AUC = 0.8260); another omitting %TWL at year one (AUC = 0.7214). Third, we assessed a model adding cardiometabolic disease (T2D, dyslipidemia, hypertension) at baseline as additional predictors. With this latter model, we found that only dyslipidemia remained significant and improved the model minimally (AUC = 0.8749). We also assessed a model restricted to variables available at baseline that included sex, age, BMI and cardiometabolic disease (AUC = 0.638).

For peer review only

**eAppendix 3. Examples of calculation of predicted probability meeting one of the definitions of surgical treatment failure at year five.**

Fictitious example 1: male=0, age 43 years, BMI = 45, -23%TWL during year one, +4 kg between year one and two

a = -1.1 + 0.00545*0 + 0.00299*43 + 0.14949*45 + 0.22310*-23 + 0.15982*4
exp(a)/(1+(a)) = 0.78

Hence, predicted probability of surgical treatment failure at year five is 78%.

Fictitious example 2: male=0, age 43 years, BMI = 49, -33%TWL during year one, -1 kg between year one and two,

a = -1.1 + 0.00545*0 + 0.00299*43 + 0.14949*49 + 0.22310*-33 + 0.15982*-1

This yields a predicted probability of 23.8% for surgical treatment failure at year five.

For peer review only

**eFigure 1 a-g. Unadjusted mean values (bars) with error bars (95% confidence intervals) for participants <u>with</u> pharmacological treatment of dyslipidemia and/or type 2 diabetes or hypertension at baseline, stratified on surgical treatment failure. a) low density lipoprotein, b) high density lipoprotein, c) triglycerides, d) fasting glucose, e) HbA1c, f) systolic blood pressure, g) diastolic blood pressure. Tables below figures show number available at each timepoint and prevalence above cut-off. Bold font with * indicates a difference p<0.05).**

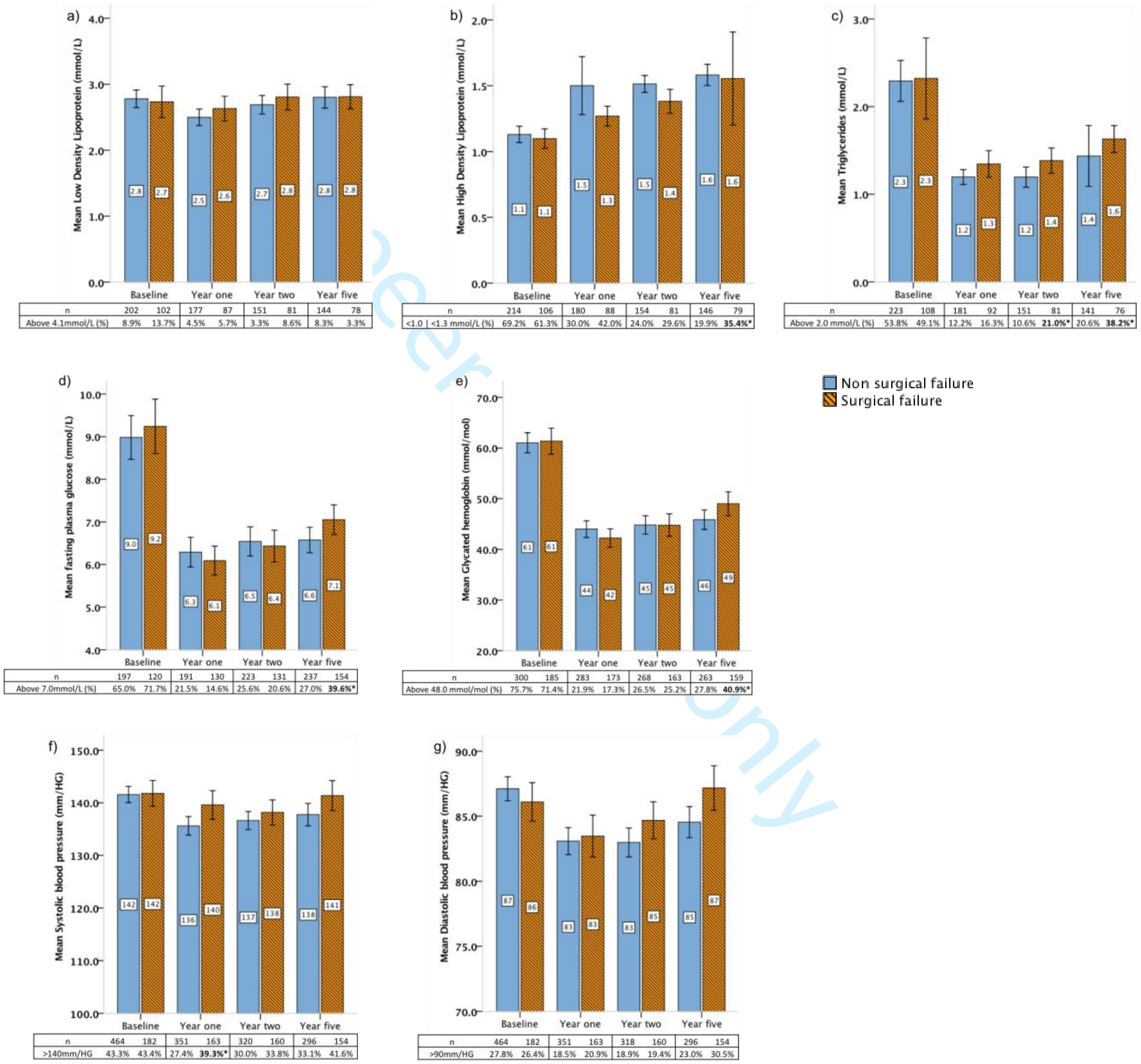


For peer review only

**eFigure 2 a-g. Unadjusted mean values (bars) with error bars (95% confidence intervals) for participants <u>without</u> pharmacological treatment of dyslipidemia and/or type 2 diabetes or hypertension at baseline, stratified on surgical treatment failure. a) low density lipoprotein, b) high density lipoprotein, c) triglycerides, d) fasting glucose, e) HbA1c, f) systolic blood pressure, g) diastolic blood pressure. Tables below figures show number available at each timepoint and prevalence above cut-off. Bold font with * indicates a difference (p<0.05).**

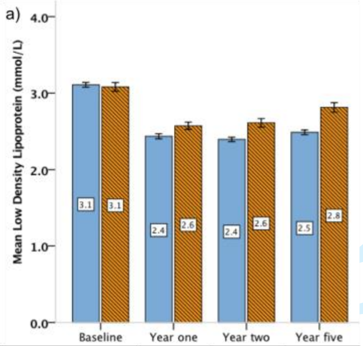


| | Baseline | | Year one | | Year two | | Year five | |
|---|---|---|---|---|---|---|---|---|
| n | 2904 | 902 | 2570 | 796 | 2289 | 679 | 2320 | 733 |
| Above 4.1mmol/L (%) | 11.1% | 11.1% | 1.0% | **2.4%*** | 1.1% | **2.2%*** | 2.1% | **5.7%*** |

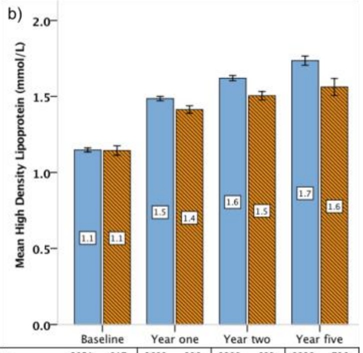


| | Baseline | | Year one | | Year two | | Year five | |
|---|---|---|---|---|---|---|---|---|
| n | 2951 | 917 | 2609 | 806 | 2300 | 682 | 2330 | 736 |
| <1.0 \| <1.3 mmol/L (%) | 71.7% | 71.3% | 30.0% | 33.1% | 17.9% | **23.9%*** | 14.2% | **22.8%*** |

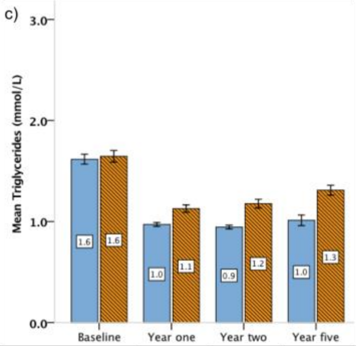


| | Baseline | | Year one | | Year two | | Year five | |
|---|---|---|---|---|---|---|---|---|
| n | 3039 | 944 | 2621 | 814 | 2302 | 681 | 2309 | 736 |
| Above 2.0 mmol/L (%) | 30.1% | 33.9% | 4.7% | **9.8%*** | 4.9% | **12.8%*** | 5.6% | **18.8%*** |

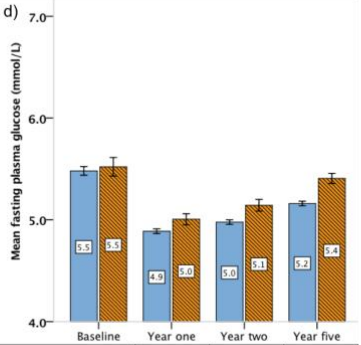


| | Baseline | | Year one | | Year two | | Year five | |
|---|---|---|---|---|---|---|---|---|
| n | 1978 | 566 | 1997 | 577 | 2095 | 589 | 2240 | 672 |
| Above 7.0mmol/L (%) | 4.0% | 4.6% | 0.3% | 0.9% | 0.4% | **1.4%*** | 0.7% | **1.8%*** |

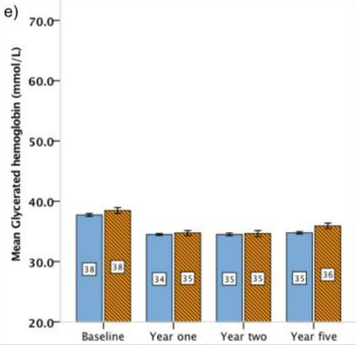


| | Baseline | | Year one | | Year two | | Year five | |
|---|---|---|---|---|---|---|---|---|
| n | 2848 | 835 | 2671 | 765 | 2363 | 667 | 2252 | 680 |
| Above 48.0 mmol/mol (%) | 3.5% | **5.4%*** | 0.4% | 0.3% | 0.5% | 0.4% | 0.3% | **1.3%*** |

Non surgical failure
Surgical failure

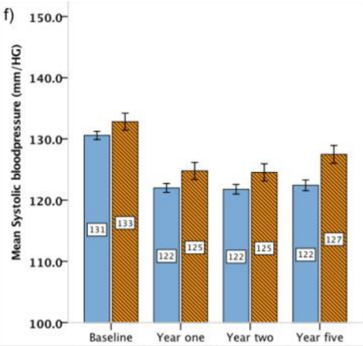


| | Baseline | | Year one | | Year two | | Year five | |
|---|---|---|---|---|---|---|---|---|
| n | 1810 | 505 | 1290 | 382 | 1259 | 363 | 1175 | 379 |
| >140mm/HG | 17.6% | 21.2% | 7.8% | 10.2% | 7.5% | 7.7% | 9.5% | **15.3%*** |

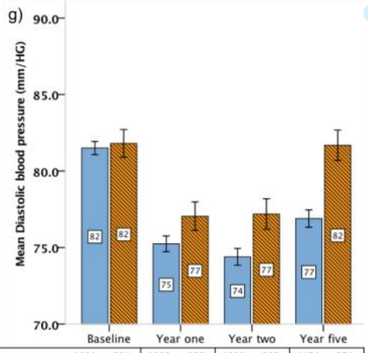


| | Baseline | | Year one | | Year two | | Year five | |
|---|---|---|---|---|---|---|---|---|
| n | 1810 | 504 | 1289 | 382 | 1260 | 363 | 1174 | 374 |
| >90mm/HG | 11.2% | 13.3% | 5.1% | 6.3% | 5.0% | **8.0%*** | 8.3% | **16.0%*** |


For peer review only - http://bmjopen.bmj.com/site/about/guidelines.xhtml

**eFigure 3.a-c. Predicted probability of type 2 diabetes at year five after surgery plotted over a, percentage weight loss from baseline to year five, b, excess body mass index loss from baseline to year five, c, body mass index at year five. Groups based on presence on presence of type 2 diabetes at baseline. Adjusted for age, sex, body mass index and type 2 diabetes at baseline.**

a,

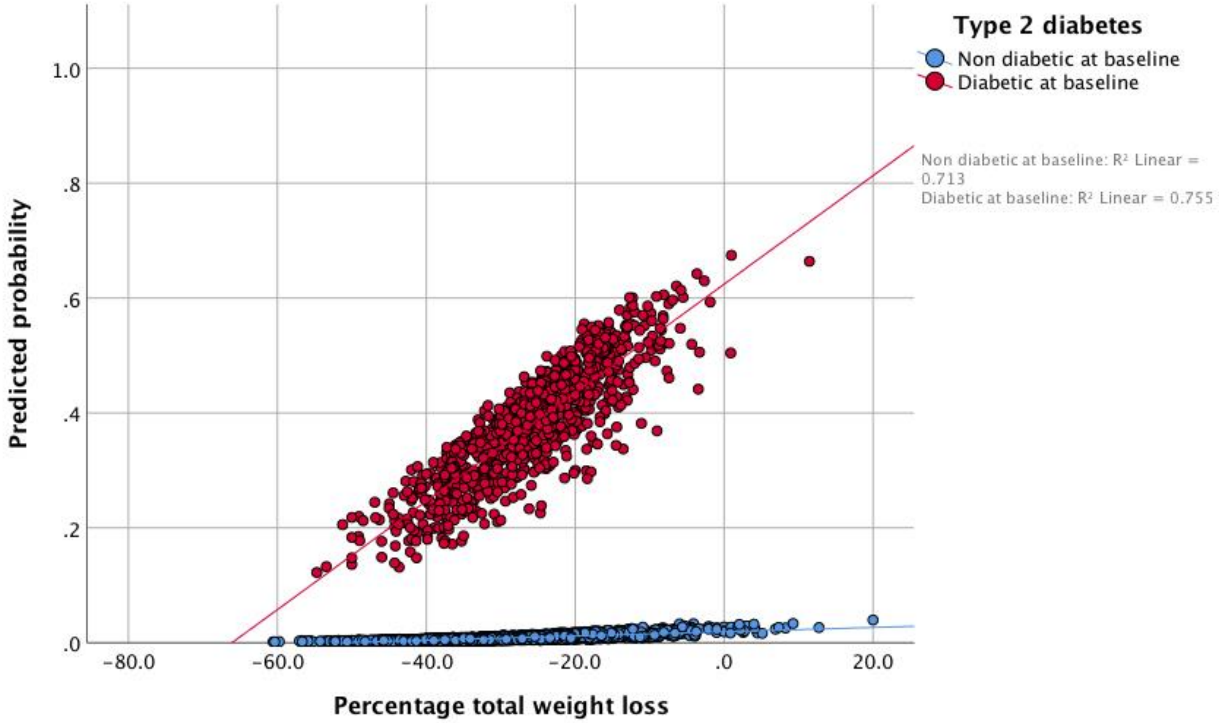


For peer review only

b,

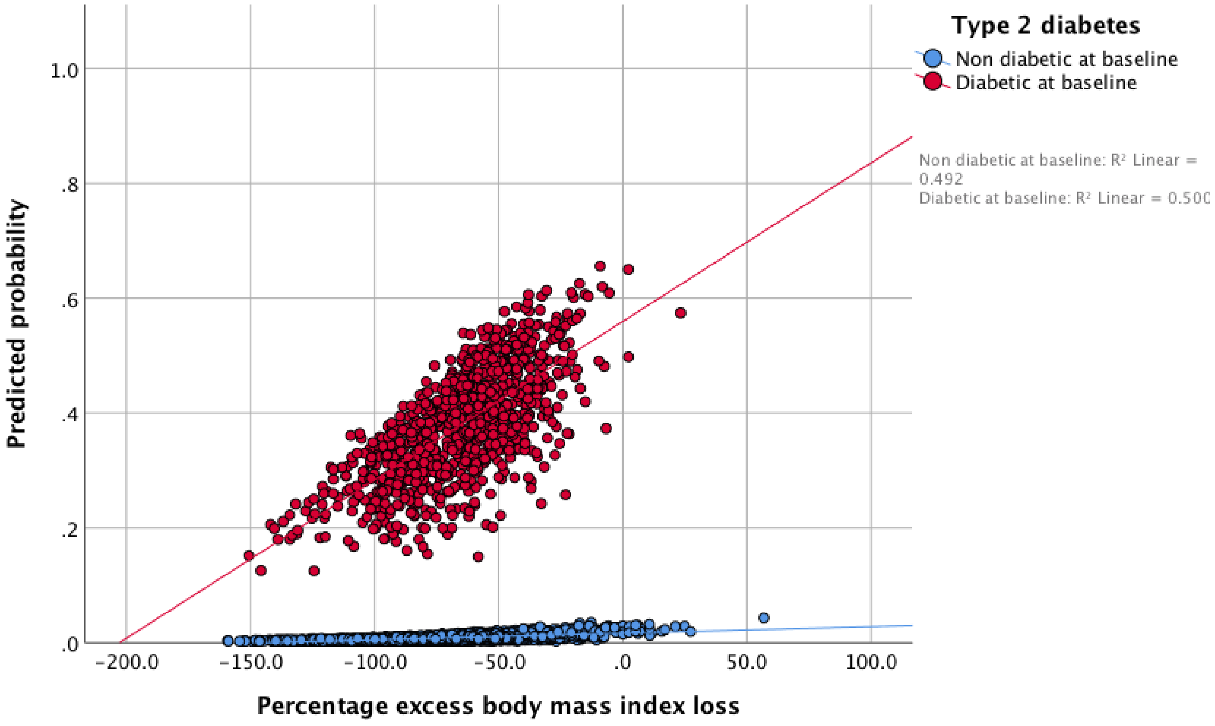
Type 2 diabetes
Non diabetic at baseline
Diabetic at baseline
Non diabetic at baseline: R² Linear = 0.492
Diabetic at baseline: R² Linear = 0.500
Predicted probability
1.0
.8
.6
.4
.2
.0
-200.0
-150.0
-100.0
-50.0
.0
50.0
100.0
Percentage excess body mass index loss

c,

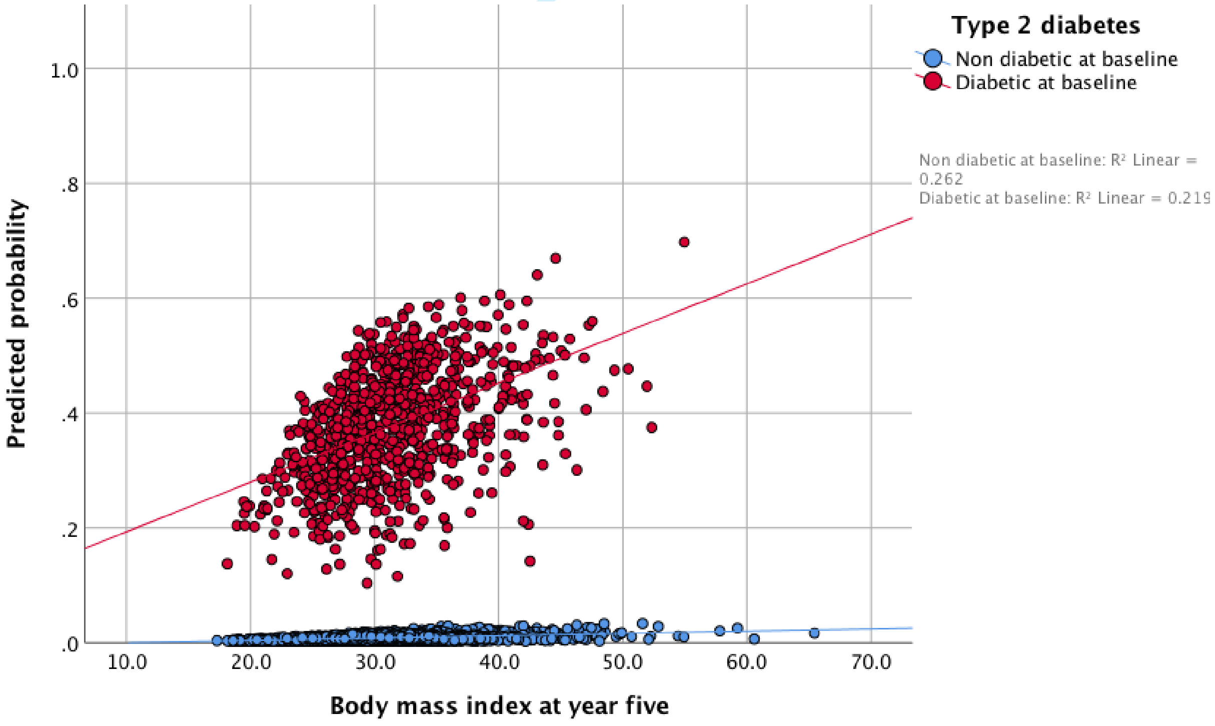
Type 2 diabetes
Non diabetic at baseline
Diabetic at baseline
Non diabetic at baseline: R² Linear = 0.262
Diabetic at baseline: R² Linear = 0.219
Predicted probability
1.0
.8
.6
.4
.2
.0
10.0
20.0
30.0
40.0
50.0
60.0
70.0
Body mass index at year five

**eFigure 4.a-c .Predicted probability of dyslipidemia at year five after surgery plotted over a, percentage weight loss from baseline to year five, b, excess body mass index loss from baseline to year five, c, body mass index at year five. Groups based on presence of dyslipidemia at baseline. Adjusted for age, sex, baseline body mass index and dyslipidemia at baseline.**

a,

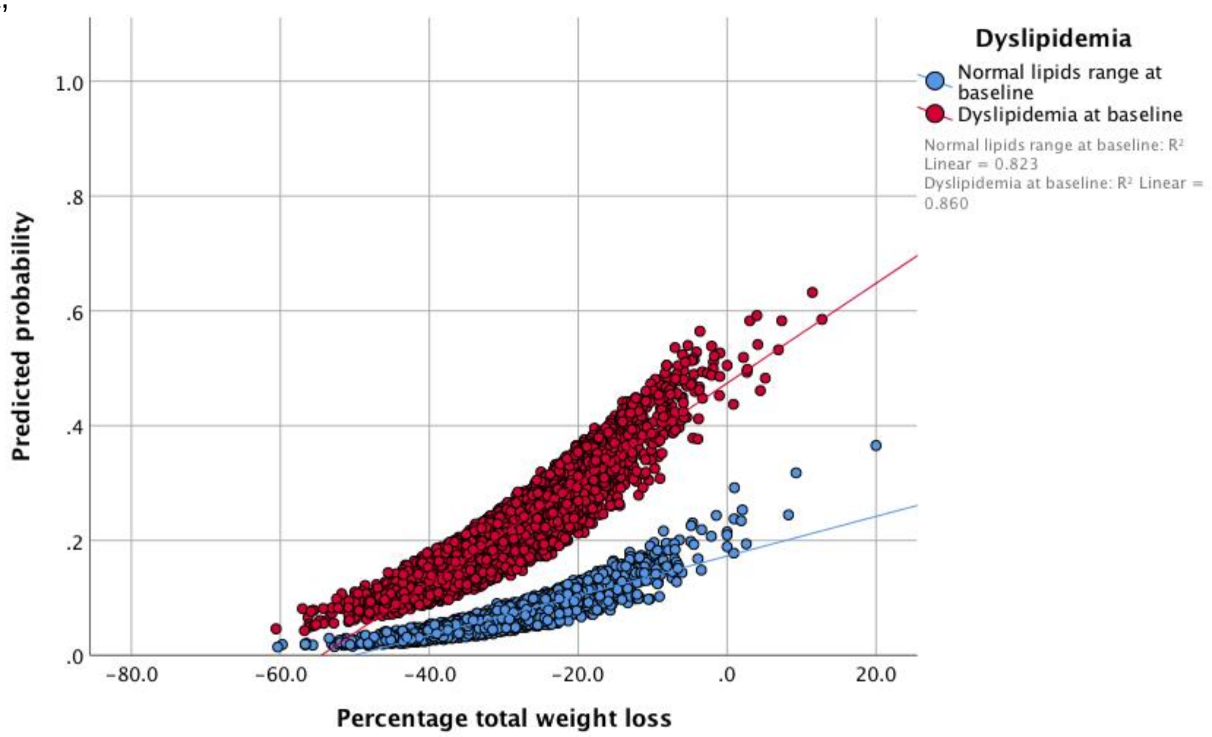



For peer review only - http://bmjopen.bmj.com/site/about/guidelines.xhtml

b,

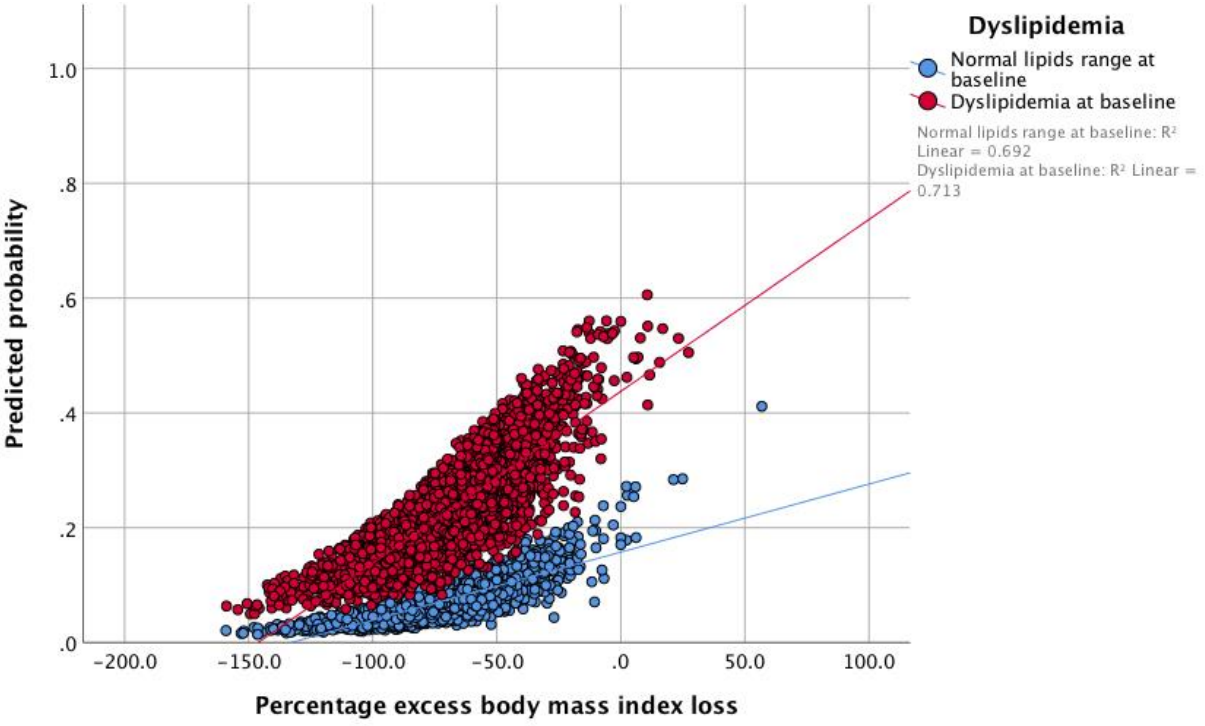


c,

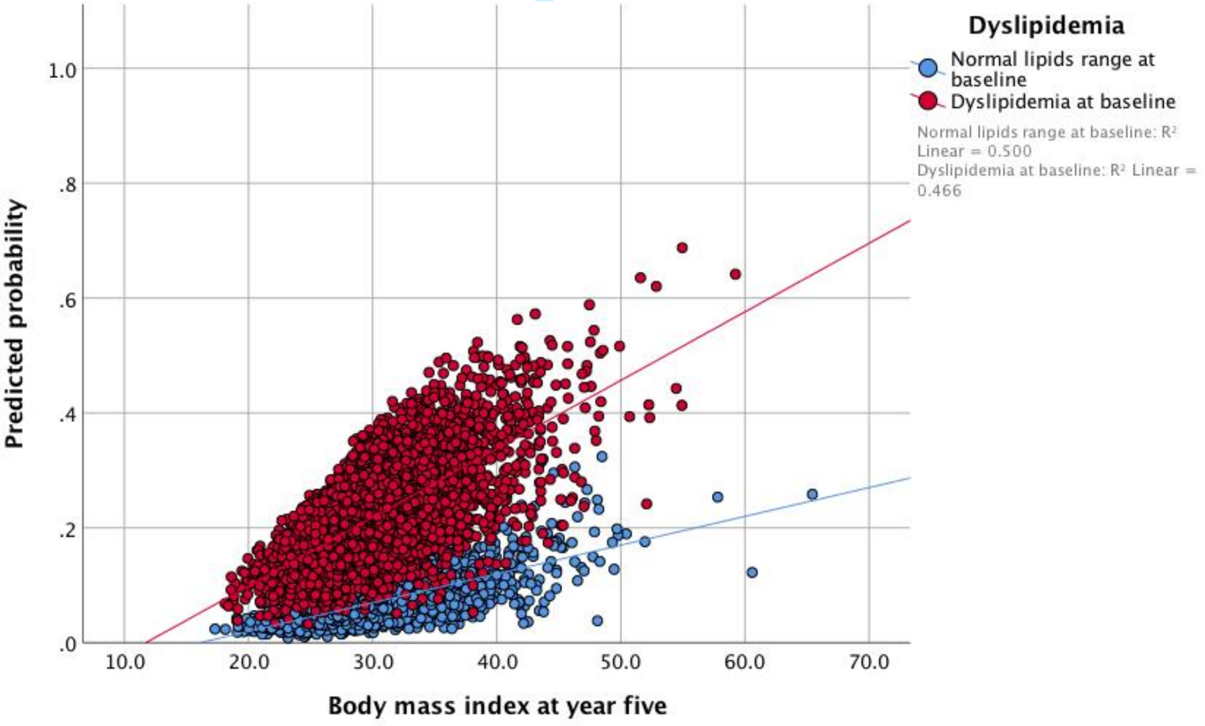



For peer review only - http://bmjopen.bmj.com/site/about/guidelines.xhtml

**eFigure 5.a-c. Predicted probability of Hypertension at year five after surgery plotted over a, percentage weight loss from baseline to year five, b, excess body mass index loss from baseline to year five, c, body mass index at year five. Groups based on presence of hypertension at baseline. Adjusted for age, sex, body mass index and hypertension at baseline.**

a,

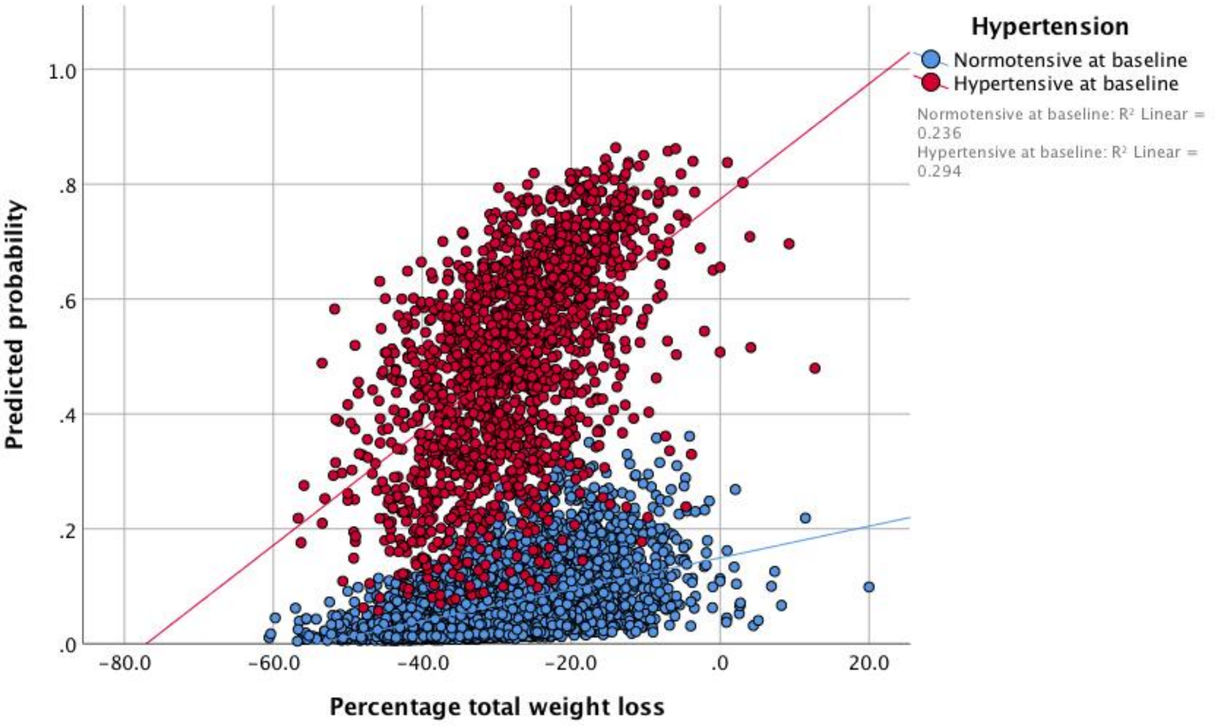



For peer review only - http://bmjopen.bmj.com/site/about/guidelines.xhtml

b,

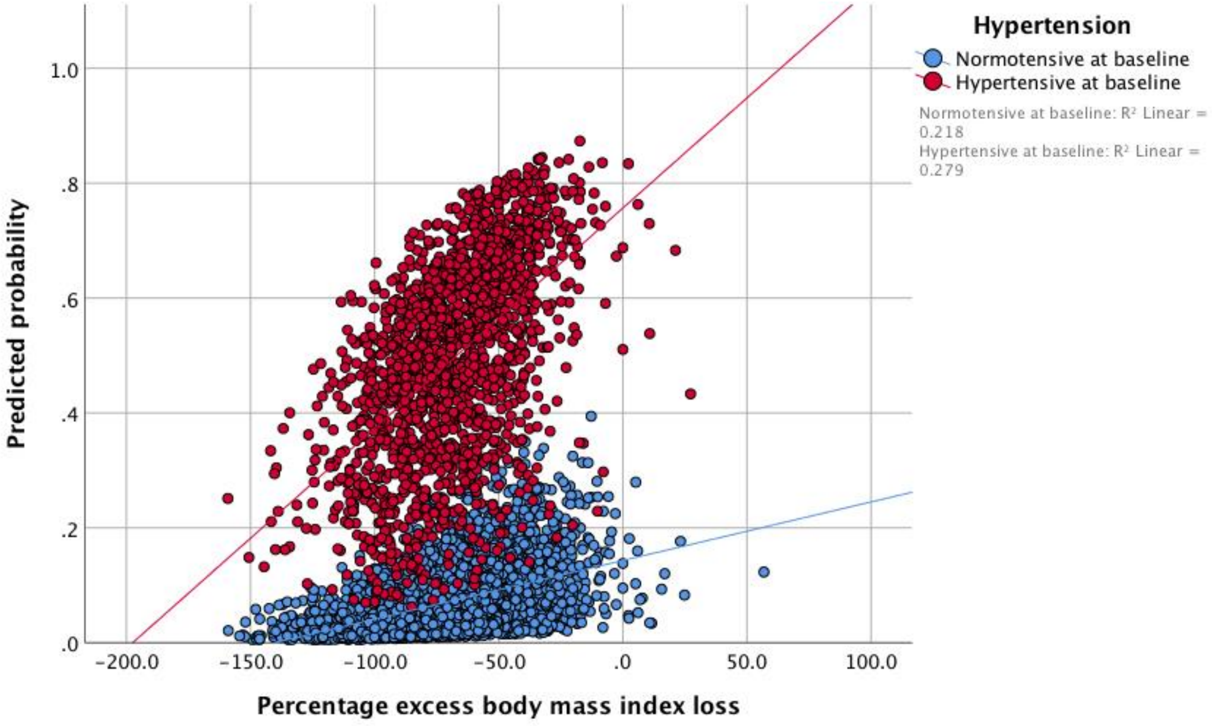


c,

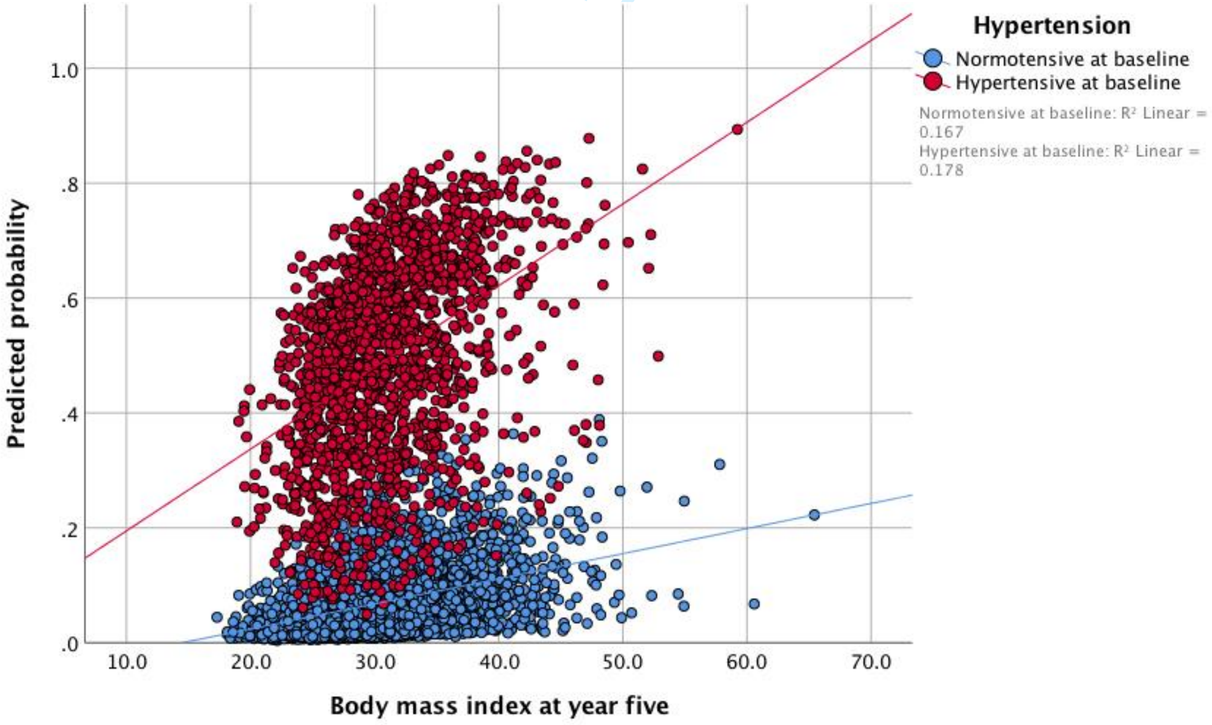



For peer review only - http://bmjopen.bmj.com/site/about/guidelines.xhtml

**eTable 1.** Modality of the one-, two-, and five year follow-ups

| | Year one n (%) | Year two n (%) | Year five n (%) |
|---|---|---|---|
| Clinical visit | 4636 (78·1) | 3543 (60·0) | 2651 (44·7) |
| Telephone consultation | 729 (12·3) | 1172 (19·7) | 2170 (36·6) |
| E-mail/letter | 217 (3·7%) | 458 (7·7) | 1087 (18·3) |
| Unspecified | 14 (0·2) | 23 (0·4) | 28 (0·5) |
| Missing | 340 (5·7) | 740 (12·5) | 0 (0·0) |

For peer review only



For peer review only - http://bmjopen.bmj.com/site/about/guidelines.xhtml

## eTable 2. Comparison of baseline characteristics between included participants and those lost to follow-up at year five

| | Included | | Lost to follow-up | | |
|---|---|---|---|---|---|
| | n | mean (sd) | n | mean (sd) | P |
| Age at surgery | 5936 | 39.4 (9.0) | 2375 | 37.6 (9.1) | <0.0001 |
| Sex, % female | 5936 | 79.10% | 2375 | 73.6% | <0.0001 |
| Height, cm | 5936 | 168.8 (8.9) | 2375 | 169.8 (9.3) | <0.0001 |
| Weight, kg | 5936 | 122.8 (20.0) | 2375 | 126.3 (20.7) | <0.0001 |
| BMI at surgery, $kg/m^2$ | 5936 | 42.9 (5.1) | 2375 | 43.6 (5.2) | <0.0001 |
| | | | | | |
| Glucose, mmol/l | 2861 | 5.9 (1.9) | 1312 | 5.8 (1.8) | 0.223 |
| HbA1c, mmol/mol | 4168 | 40.6 (11.4) | 1846 | 40.8 (11.7) | 0.528 |
| Pharmacological diabetes treatment | 5936 | 11.4% | 2375 | 9.6% | 0.022 |
| Diabetes type 2[A] | 5936 | 15.1% | 2375 | 14.6% | 0.543 |
| | | | | | |
| High-density lipoprotein, mmol/l | 4188 | 1.2 (0.4) | 1845 | 1.1 (0.5) | 0.017 |
| Low-density lipoprotein, mmol/l | 4110 | 3.1 (0.9) | 1798 | 3.1 (1.0) | 0.756 |
| Triglycerides, mmol/l | 4314 | 1.7 (1.4) | 1883 | 1.8 (2.2) | 0.087 |
| Pharmacological dyslipidemia treatment | 5936 | 7% | 2375 | 6.5% | 0.465 |
| Dyslipidemia[B] | 5936 | 67.5% | 2375 | 74.1% | <0.0001 |
| | | | | | |
| Systolic BP, mmHg | 2960 | 133 (16) | 1348 | 135 (17) | 0.015 |
| Diastolic BP, mmHg | 2960 | 83 (10) | 1347 | 83 (10) | 0.164 |
| Pharmacological hypertension treatment | 5936 | 19.5% | 2375 | 18.1% | 0.142 |
| Hypertension[C] | 5936 | 28.4% | 2375 | 29.3% | 0.365 |

BMI – Body mass index

[A] Pharmacologically treated T2D | fasting glucose >7.0mmol/l | HbA1c >48mmol/mol
[B] Pharmacologically treated dyslipidemia | LDL >4.1 | TG > 2.0 | HDL <1.0mmol/L for males and <1.3mmol/L for females
[C] Pharmacologically treated blood pressure | systolic- >140mm/Hg or diastolic blood pressure >90mm/Hg

For peer review only

**eTable 3. Percentage of the 5936 participants with available data on pharmacological treatment and on biochemistry and blood pressure at baseline, one, two and five years after surgery.**

| | Baseline | Year one | Year two | Year five |
|---|---|---|---|---|
| Pharmacological treatment | | | | |
| Type 2 diabetes | 100% | 94% | 88% | 100% |
| Dyslipidemia | 100% | 94% | 88% | 100% |
| Hypertension | 100% | 94% | 88% | 100% |
| | | | | |
| Biochemistry | | | | |
| Low-density lipoprotein | 69% | 61% | 54% | 55% |
| High-density lipoprotein | 71% | 62% | 54% | 55% |
| Triglycerides | 73% | 63% | 54% | 55% |
| Fasting glucose | 48% | 49% | 51% | 56% |
| HbA1c | 70% | 66% | 58% | 57% |
| Blood pressure | 50% | 37% | 35% | 34% |

For peer review only


For peer review only - http://bmjopen.bmj.com/site/about/guidelines.xhtml

**eTable 4. Comparison of baseline characteristics between surgical treatment failure (STF) and non-STF**

| | STF | | Non-STF | | |
|---|---|---|---|---|---|
| | n | mean (sd) | n | mean (sd) | P |
| Age at surgery | 1371 | 40.5 (8.8) | 4565 | 39.1 (9.0) | <0.001 |
| Sex, % female | 1371 | 71.4% | 4565 | 81.4% | <0.001 |
| Height, cm | 1371 | 169.6 (9.4) | 4565 | 168.6 (8.7) | <0.001 |
| Weight, kg | 1371 | 128.2 (22.9) | 4565 | 121.2 (18.7) | <0.001 |
| BMI at surgery, kg/m$^2$ | 1371 | 44.4 (6.1) | 4565 | 42.5 (4.6) | <0.001 |
| **Glucose Metabolism** | | | | | |
| Glucose, mmol/l | 686 | 6.2 (2.3) | 2175 | 5.8 (1.8) | <0.001 |
| HbA1c, mmol/mol | 1020 | 42.6 (13.3) | 3148 | 40.0 (10.7) | <0.001 |
| Pharmacological Diabetes treatment | 1371 | 17.4% | 4565 | 9.6% | <0.001 |
| Diabetes type 2[A] | 1371 | 22.0% | 4565 | 13.0% | <0.001 |
| **Lipids** | | | | | |
| High-density lipoprotein, mmol/l | 1023 | 1.1 (0.5) | 3165 | 1.2 (0.4) | 0.605 |
| Low-density lipoprotein, mmol/l | 1004 | 3.1 (0.9) | 3106 | 3.1 (0.9) | 0.201 |
| Triglycerides, mmol/l | 1052 | 1.7 (1.2) | 3262 | 1.7 (1.4) | 0.285 |
| Pharmacological dyslipidemia treatment | 1371 | 9.6% | 4565 | 6.2% | <0.001 |
| Dyslipidemia[B] | 1371 | 72.3% | 4565 | 66.0% | <0.001 |
| **Physiology** | | | | | |
| Systolic BP, mmHg | 687 | 135 (16) | 2274 | 133 (16) | 0.001 |
| Diastolic BP, mmHg | 686 | 83 (11) | 2274 | 83 (10) | 0.509 |
| Pharmacological hypertension treatment | 1371 | 24.8% | 4565 | 17.9% | <0.001 |
| Hypertension[C] | 1371 | 34.2% | 4565 | 26.6% | <0.001 |

[A] Pharmacologically treated T2D | fasting glucose >7.0mmol/l | HbA1c >48mmol/mol
[B] Pharmacologically treated dyslipidemia | LDL >4.1 | TG > 2.0 | HDL <1.0mmol/L for males and <1.3mmol/L for females
[C] Pharmacologically treated blood pressure | systolic- >140mm/Hg or diastolic blood pressure >90mm/Hg

For peer review only

**eTable 5. Odds of cardiometabolic disease at year five, separate models for each definitions of surgical treatment failure. Adjusted for sex and baseline; age, BMI and corresponding cardiometabolic disease.**

| | Beta | Standard error | Odds ratio | 95% Confidence interval Lower | Upper | p |
|---|---|---|---|---|---|---|
| **Type 2 diabetes** | | | | | | |
| Non surgical treatment failure (ref) | | | | | | |
| Total weight loss <20% | 0.818 | 0.142 | 2.266 | 1.715 | 2.995 | <0.001 |
| Excess BMI loss <50% | 0.760 | 0.144 | 2.138 | 1.611 | 2.837 | <0.001 |
| BMI >35 or >40* | 0.893 | 0.184 | 2.441 | 1.703 | 3.499 | <0.001 |
| Compiled** | 0.743 | 0.137 | 2.102 | 1.608 | 2.749 | <0.001 |
| **Dyslipidemia** | | | | | | |
| Non surgical treatment failure (ref) | | | | | | |
| Total weight loss <20% | 0.946 | 0.084 | 2.574 | 2.185 | 3.033 | <0.001 |
| Excess BMI loss <50% | 0.935 | 0.083 | 2.548 | 2.164 | 3.000 | <0.001 |
| BMI >35 or >40* | 0.863 | 0.100 | 2.370 | 1.949 | 2.883 | <0.001 |
| Compiled** | 0.916 | 0.079 | 2.500 | 2.143 | 2.918 | <0.001 |
| **Hypertension** | | | | | | |
| Non surgical treatment failure (ref) | | | | | | |
| Total weight loss <20% | 0.687 | 0.098 | 1.988 | 1.642 | 2.407 | <0.001 |
| Excess BMI loss <50% | 0.652 | 0.095 | 1.920 | 1.593 | 2.315 | <0.001 |
| BMI >35 or >40* | 0.569 | 0.114 | 1.767 | 1.413 | 2.210 | <0.001 |
| Compiled** | 0.616 | 0.089 | 1.851 | 1.554 | 2.206 | <0.001 |

*For subjects with presurgery BMI of <50 and >50, respectively.
**Defined as meeting any of the definitions, <20%TWL | <50%EBMIL | BMI >35 or >40.

For peer review only

**eTable 6. Odds of Cardiometabolic disease at year five for subjects reaching one, two or three of the three definitions (exclusively, subjects may only be in one group). Adjusted for sex and baseline; age, BMI and corresponding cardiometabolic disease.**

| | Beta | Standard error | Odds ratio | 95% Confidence Interval Lower | Upper | p |
|---|---|---|---|---|---|---|
| **Type 2 diabetes** | | | | | | |
| Non surgical treatment failure (ref) | | | | | | |
| Surgical failure 1/3 | 0.409 | 0.258 | 1.506 | 0.908 | 2.496 | 0.113 |
| Surgical failure 2/3 | 0.592 | 0.186 | 1.808 | 1.255 | 2.606 | 0.001 |
| Surgical failure 3/3 | 1.119 | 0.198 | 3.061 | 2.078 | 4.509 | <0.001 |
| **Dyslipidemia** | | | | | | |
| Non surgical treatment failure (ref) | | | | | | |
| Surgical failure 1/3 | 0.605 | 0.147 | 1.832 | 1.373 | 2.445 | <0.001 |
| Surgical failure 2/3 | 0.961 | 0.111 | 2.614 | 2.103 | 3.250 | <0.001 |
| Surgical failure 3/3 | 1.050 | 0.111 | 2.857 | 2.299 | 3.550 | <0.001 |
| **Hypertension** | | | | | | |
| Non surgical treatment failure (ref) | | | | | | |
| Surgical failure 1/3 | 0.415 | 0.165 | 1.515 | 1.097 | 2.091 | 0.012 |
| Surgical failure 2/3 | 0.556 | 0.130 | 1.744 | 1.352 | 2.248 | <0.001 |
| Surgical failure 3/3 | 0.798 | 0.129 | 2.221 | 1.724 | 2.862 | <0.001 |

For peer review only



For peer review only - http://bmjopen.bmj.com/site/about/guidelines.xhtml

STROBE Statement—checklist of items that should be included in reports of observational studies

| | Item No | Recommendation | Page No |
|---|---|---|---|
| **Title and abstract** | 1 | (*a*) Indicate the study's design with a commonly used term in the title or the abstract | 1 |
| | | (*b*) Provide in the abstract an informative and balanced summary of what was done and what was found | 3-4 |
| **Introduction** | | | |
| Background/rationale | 2 | Explain the scientific background and rationale for the investigation being reported | 5-6 |
| Objectives | 3 | State specific objectives, including any prespecified hypotheses | 6 |
| **Methods** | | | |
| Study design | 4 | Present key elements of study design early in the paper | 6-7 |
| Setting | 5 | Describe the setting, locations, and relevant dates, including periods of recruitment, exposure, follow-up, and data collection | 6-7 |
| Participants | 6 | (*a*) *Cohort study*—Give the eligibility criteria, and the sources and methods of selection of participants. Describe methods of follow-up<br>*Case-control study*—Give the eligibility criteria, and the sources and methods of case ascertainment and control selection. Give the rationale for the choice of cases and controls<br>*Cross-sectional study*—Give the eligibility criteria, and the sources and methods of selection of participants | 7 |
| | | (*b*) *Cohort study*—For matched studies, give matching criteria and number of exposed and unexposed<br>*Case-control study*—For matched studies, give matching criteria and the number of controls per case | |
| Variables | 7 | Clearly define all outcomes, exposures, predictors, potential confounders, and effect modifiers. Give diagnostic criteria, if applicable | 8-10 |
| Data sources/ measurement | 8* | For each variable of interest, give sources of data and details of methods of assessment (measurement). Describe comparability of assessment methods if there is more than one group | *8-10* |
| Bias | 9 | Describe any efforts to address potential sources of bias | 7 |
| Study size | 10 | Explain how the study size was arrived at | 7 |
| Quantitative variables | 11 | Explain how quantitative variables were handled in the analyses. If applicable, describe which groupings were chosen and why | 10-11 |
| Statistical methods | 12 | (*a*) Describe all statistical methods, including those used to control for confounding | 10-11 |
| | | (*b*) Describe any methods used to examine subgroups and interactions | 10-11 |
| | | (*c*) Explain how missing data were addressed | 7,18 |
| | | (*d*) *Cohort study*—If applicable, explain how loss to follow-up was addressed<br>*Case-control study*—If applicable, explain how matching of cases and controls was addressed<br>*Cross-sectional study*—If applicable, describe analytical methods taking account of sampling strategy | 7 |
| | | (*e*) Describe any sensitivity analyses | 10-11 |

For peer review only

Continued on next page



For peer review only - http://bmjopen.bmj.com/site/about/guidelines.xhtml

**Results**

| | | | |
|---|---|---|---|
| Participants | 13* | (a) Report numbers of individuals at each stage of study—eg numbers potentially eligible, examined for eligibility, confirmed eligible, included in the study, completing follow-up, and analysed | 6-7 |
| | | (b) Give reasons for non-participation at each stage | |
| | | (c) Consider use of a flow diagram | Figure 1 |
| Descriptive data | 14* | (a) Give characteristics of study participants (eg demographic, clinical, social) and information on exposures and potential confounders | table 1 |
| | | (b) Indicate number of participants with missing data for each variable of interest | Throughout including tables |
| | | (c) *Cohort study*—Summarise follow-up time (eg, average and total amount) | 6-7 |
| Outcome data | 15* | *Cohort study*—Report numbers of outcome events or summary measures over time | *6-10* |
| | | *Case-control study*—Report numbers in each exposure category, or summary measures of exposure | |
| | | *Cross-sectional study*—Report numbers of outcome events or summary measures | |
| Main results | 16 | (*a*) Give unadjusted estimates and, if applicable, confounder-adjusted estimates and their precision (eg, 95% confidence interval). Make clear which confounders were adjusted for and why they were included | 13 |
| | | (*b*) Report category boundaries when continuous variables were categorized | |
| | | (*c*) If relevant, consider translating estimates of relative risk into absolute risk for a meaningful time period | |
| Other analyses | 17 | Report other analyses done—eg analyses of subgroups and interactions, and sensitivity analyses | Appendix |
| **Discussion** | | | |
| Key results | 18 | Summarise key results with reference to study objectives | 15-16 |
| Limitations | 19 | Discuss limitations of the study, taking into account sources of potential bias or imprecision. Discuss both direction and magnitude of any potential bias | 18 |
| Interpretation | 20 | Give a cautious overall interpretation of results considering objectives, limitations, multiplicity of analyses, results from similar studies, and other relevant evidence | 15-18 |
| Generalisability | 21 | Discuss the generalisability (external validity) of the study results | 18 |
| **Other information** | | | |
| Funding | 22 | Give the source of funding and the role of the funders for the present study and, if applicable, for the original study on which the present article is based | 20 |

*Give information separately for cases and controls in case-control studies and, if applicable, for exposed and unexposed groups in cohort and cross-sectional studies.

**Note:** An Explanation and Elaboration article discusses each checklist item and gives methodological background and published examples of transparent reporting. The STROBE checklist is best used in conjunction with this article (freely available on the Web sites of PLoS Medicine at http://www.plosmedicine.org/, Annals of Internal Medicine at http://www.annals.org/, and Epidemiology at http://www.epidem.com/). Information on the STROBE Initiative is available at www.strobe-statement.org.

For peer review only
